# Structural basis of porcine reproductive and respiratory syndrome virus 2 neutralization by a GP4-targeting monoclonal antibody

**DOI:** 10.1099/jgv.0.002284

**Published:** 2026-06-25

**Authors:** Barbora Veselkova, Chandamita Saikia, Sebastian Affeldt, Sandra Barth, Manoj Kumar Rajak, Frithjof Besa, Daria Bezbakh, Guido Hansen, Till Rümenapf, Matthias Ballmaier, Oliver Dittrich-Breiholz, Holly Bamber, Kumar Nagarathinam, Alexander W. Tarr, Benjamin Lamp, Thomas Krey

**Affiliations:** 1 Institute of Virology, Hannover Medical School, Hannover, Germany; 2 Center of Structural and Cell Biology in Medicine, Institute of Biochemistry, University of Lübeck, Lübeck, Germany; 3 Institute of Virology, Faculty of Veterinary Medicine, Biomedical Research Center, Justus-Liebig-University, Schubertstraße 81, 35392 Gießen, Germany; 4 Infectiology and Virology, Center of Pathobiology, University of Veterinary Medicine Vienna, 1210 Vienna, Austria; 5 Central Research Facility Cell Sorting, Hannover Medical School, 30625 Hannover, Germany; 6 Research Core Unit Genomics, Hannover Medical School, 30625 Hannover, Germany; 7 Wolfson Centre for Global Virus Research, University of Nottingham, Nottingham, UK; 8 School of Life Sciences, Faculty of Medicine and Health Sciences, University of Nottingham, Nottingham, UK; 9 Partner Site Hamburg-Lübeck-Borstel-Riems, German Center for Infection Research (DZIF), Hamburg, Germany; 10 Cluster of Excellence RESIST (EXC 2155), Hannover Medical School, Hannover, Germany; 11 Centre for Structural Systems Biology (CSSB), Hamburg, Germany

**Keywords:** epitope structure, monoclonal neutralizing antibody, porcine reproductive and respiratory syndrome virus (PRRSV), virus neutralization

## Abstract

Porcine reproductive and respiratory syndrome virus (PRRSV) is a genetically diverse RNA virus that causes reproductive failure and respiratory disease in pigs, leading to major economic losses worldwide. Although inactivated and live-attenuated vaccines are available, they provide only partial protection, allowing endemic circulation, persistent transmission and recurrent outbreaks. The limited efficacy of current vaccines reflects the incomplete understanding of immune correlates of protection. Here, we describe a murine monoclonal antibody (IgG #18) with potent, strain-specific neutralizing activity against PRRSV-2. Epitope mapping localized its binding site to the N-terminal region of the minor glycoprotein GP4. Functional and biochemical analyses demonstrated high-affinity binding and effective neutralization, while crystallographic studies revealed its atomic structure in complex with an epitope peptide. To our knowledge, this structure provides the first structural snapshot of a neutralization epitope within the PRRSV glycoproteins, providing insights into the conformation of this key component of the PRRSV envelope complex. This structure also explains the restricted specificity of IgG #18, highlighting the challenge of epitope variability across PRRSV species. Our findings advance understanding of antibody-mediated neutralization and provide an initial framework for the use of structural information in the design of next-generation immunogens aimed at eliciting protective responses against genetically diverse PRRSV strains.

## Data Availability

The atomic coordinates and structure factors for the crystal structure were deposited in the Protein Data Bank (http://www.pdb.org/) under the accession code 29TJ. The scRNAseq data were deposited at the Sequence Read Archive under the accession code PRJNA1402897. All other raw data generated in this study are provided in the supplementary information or the manuscript file.

## Introduction

Porcine reproductive and respiratory syndrome virus (PRRSV) causes the porcine reproductive and respiratory syndrome (PRRS), which is extremely variable in its clinical picture. The outcome of PRRSV infection depends on both the virulence of the PRRSV strain and the breed and immune status of the herd [1]. Low-virulence strains of PRRSV cause widespread infections in the domestic swine population inducing mostly subclinical disease and only mild symptoms in single animals [2]. In contrast, highly virulent strains induce severe disease in susceptible herds resulting in abortion storms and even epidemics with mass mortality [3,7]. While respiratory illness and secondary bacterial infections are dominant in post-weaners and fattening pigs, reproductive failure is the leading symptom in gestating sows. The economic impact of PRRSV to pork producers in the USA alone, caused by the reduction or loss of pregnancies, death in young piglets and decreased growth rates in all PRRSV-infected pigs, is estimated to reach 1.2 billion USD annually [8], increasing nearly twofold over the last decade [9].

Despite extensive research since PRRSV first appeared more than 30 years ago, there is still no effective prophylaxis available that safely protects herds from infections. Several modified live vaccines (MLVs) against both PRRSV-1 and PRRSV-2 have been licensed in various countries depending on circulating viral genotypes; they are applied as prophylaxis in non-infected and metaphylaxis in affected herds. This leads to a simultaneous circulation of field and vaccine strain causing safety concerns about frequent recombination with field strains [10,13]. In addition, a potential reversion of the MLV strains to virulence is possible (reviewed in [14]), and available inactivated vaccines offer poor efficacies that are possibly caused by insufficient induction of neutralizing antibodies (nAbs) [15,16].

PRRSV is an enveloped, positive-sense single-stranded RNA virus belonging to the genus *Betaarterivirus* of the family *Arteriviridae* within the order *Nidovirales* [17]. The virus is characterized by a high genetic variability illustrated by a low nucleotide sequence identity of only 60% between the Lelystad and VR-2332 strains (prototypes of *Betaarterivirus europensis* and *americense* species, respectively) [18]. These two separate species can be distinguished based on serological differences despite large genetic and phenotypic homologies, such as genome organization, encoded proteins and particle morphology [17]. The induced disease patterns in the respiratory and reproductive tracts are similar between the two species, and immunological cross-protection has been observed [19].

PRRSV infection elicits a complex immune response involving both CD8+ cytotoxic T cells and nAbs; however, the role of these two defence mechanisms for virus clearance remains unclear. Recently, accumulating evidence has highlighted the importance of nAbs in the antiviral defence. Reduced viraemia and milder outcome of PRRSV re-infections have been attributed primarily to the humoral immune response [20]. Similarly, the appearance of nAbs in primary infections is directly correlated with virus clearance from the bloodstream and most tissues. Eventually, administration of single doses of a PRRSV-specific mouse monoclonal antibody (PN9cx3) markedly alleviated the clinical outcome of PRRSV infection [21]. It remains, however, elusive to date, which glycoprotein nAbs should target to protect from or minimize the clinical symptoms caused by PRRSV infection.

The humoral immune response in PRRSV infection is characterized by a delayed appearance of nAbs only 3 to 6 weeks post-infection [20], contributing to an insufficient control of viral replication and allowing the establishment of persistent infections. An extensive glycan shielding together with the antigenic variability observed in the PRRSV glycoproteins further limits the efficacy of the induced humoral immune response [22]. While nAbs can, in most cases, effectively neutralize homologous strains, cross-protection against heterologous variants is often limited. These features highlight the challenge of defining structurally conserved neutralization epitopes within PRRSV glycoproteins.

The PRRSV virion contains several structural proteins that interact with the host immune system, including the glycoproteins GP2a, GP3, GP4 and GP5; the membrane protein (M); and the envelope protein (E). A number of structural proteins are targeted by nAbs [23,28], among them the major glycoprotein GP5 and the minor glycoproteins GP2a, GP3 and GP4. Some of them are shielded by extensive glycosylation, which reduces their accessibility to antibodies [22]. Several linear neutralization epitopes have been identified within these glycoproteins (reviewed in [29]). Most of these epitopes are variable, but a few conserved neutralization epitopes have been identified, including one in GP4 [30]. Of note, the contribution of individual epitopes to a protective immune response in pigs remains elusive to date [31]. Identification of neutralization epitopes together with recent advances in structural biology offers new opportunities for rational vaccine design, such as the design of epitope-focused vaccines that selectively present conserved neutralization epitopes while minimizing immune diversion to decoy epitopes, as described for several important viral pathogens [32,34]. Currently, there is no structural information on neutralization epitopes within the PRRSV glycoproteins reported.

Here, we describe the isolation of a novel monoclonal neutralizing antibody from mice (IgG #18) resulting from immunization with virions representing PRRSV-2 followed by a boost with a homologous recombinant single-chain GP2-GP4 produced in *Drosophila* S2 cells. Peptide phage display together with MBP (maltose-binding protein)-peptide ELISA identified an epitope located in the N-terminal region of GP4 that reacted with IgG #18. The X-ray structure of a single-chain variable fragment (scFv) in complex with an epitope peptide revealed structural insights into the interaction of this minor PRRSV glycoprotein with the humoral immune response and highlighted the dominant contact residues within this novel epitope. Our findings provide a first structural understanding of neutralizing determinants within the PRRSV glycoproteins. This structural knowledge may help inform the rational design of novel immunogens and provides a proof-of-concept for structure-guided vaccine design strategies in PRRSV vaccinology.

## Methods

### Cell culture

*Drosophila* Schneider 2 (S2) cells were purchased from Invitrogen and cultured in Schneider’s *Drosophila* medium at 28 °C in tissue culture flask in a non-humidified incubator. Stable cell lines were transferred to serum-free Insect Xpress media (Lonza, Basel, Switzerland) and with larger volumes to polycarbonate Erlenmeyer flasks (Corning), which were used for protein production at 70 r.p.m. ExPi293F cells (Thermo Fisher Scientific) were cultured in Gibco Expi293 Expression Medium and a CO_2_ incubator (125 r.p.m., 37 °C, 80% humidity, 8% CO_2_). MARC-145 (RRID: CVCL_4540) were cultivated in Dulbecco’s modified Eagle’s medium supplemented with 10% FCS (Gold Bio and Sell, Feucht, Germany) at 37 °C with 5% CO_2_.

### Recombinant protein expression and purification

For sc-GP2GP4 and sc-GP2GP4-mNeonGreen protein production, a codon-optimized gene coding for PRRSV-2 VR-2332 GP2 ectodomain (residues 41–208) was linked to the VR-2332 GP4 ectodomain (residues 20–161) via a soluble 19 amino acid glycine–serine linker (GGGGSGGGGSGGGGSGGGG) and cloned into a *Drosophila* S2 expression vector featuring an N-terminal BiP-signal sequence and a C-terminal enterokinase (EK)-cleavage site prior to a double Strep tag (DST) [35]. The expression vector for sc-GP2GP4-mNeonGreen construct contained an additional sequence for mNeonGreen fluorophore preceding the DST. After signal peptide removal, an additional arginine and serine remained at the N-terminus of sc-GP2GP4 as remnant of a restriction nuclease cleavage site. For scFv production, codon-optimized synthetic genes (Twist Bioscience, San Francisco, USA) encoding heavy and light chains of the variable regions were cloned into a *Drosophila* S2 scFv expression vector containing an N-terminal BiP-signal peptide and an EK-cleavable C-terminal DST [36].

**Fig. 1.**
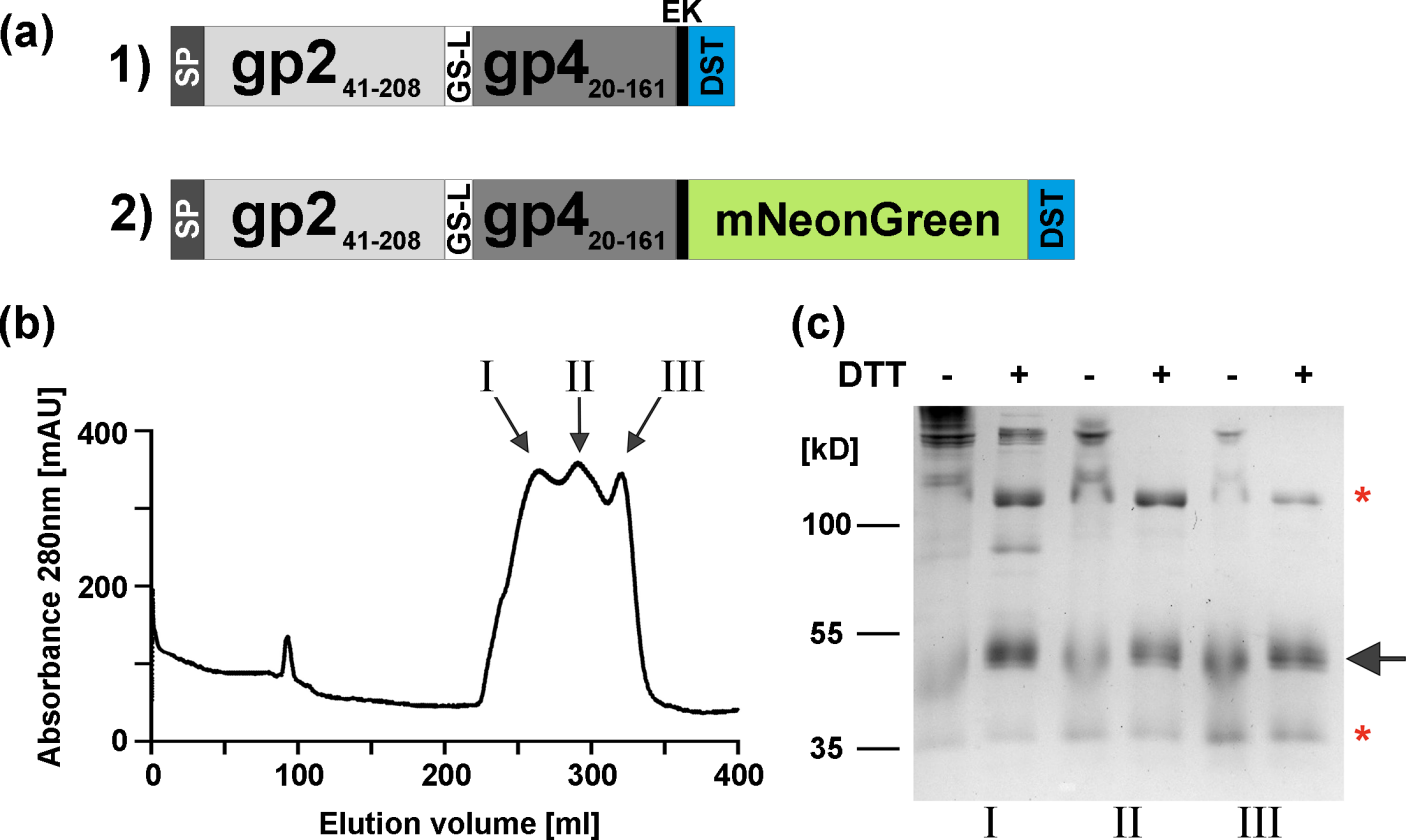
Expression of a soluble single-chain GP2GP4 (sc-GP2GP4). (a) Schematic drawing of the two sc-GP2GP4 expression constructs used in this study. Numbers indicate amino acid positions within the PRRSV glycoproteins. SP, signal peptide; GS-L, glycine–serine linker; EK, enterokinase cleavage site; DST, double Strep tag. (b, c) Elution profile of sc-GP2GP4 analysed on two Superdex 200 26/600 pg size exclusion columns connected in tandem. Roman numerals highlight the three eluting peaks; corresponding elution fractions were analysed by SDS-PAGE under reducing/non-reducing conditions followed by Coomassie staining. Two red asterisks indicate the two *D. melanogaster* proteins that co-purify with sc-GP2GP4. The arrow highlights sc-GP2GP4.

Stable *Drosophila* S2 cell lines were generated via transfection with the respective expression plasmids using Effectene (Qiagen) as described previously [37]. For large-scale protein production, stable cell lines were induced with 4 µM CdCl_2_ at a density of ~3×10^6^ cells ml^−1^ for 6 days. Cells were pelleted, and sc-GP2GP4 and sc-GP2GP4-mNeonGreen were purified from the cell supernatant by affinity chromatography using a self-packed Tricorn column (Cytiva) with Strep-Tactin^®^ XT Superflow resin (IBA, Göttingen, Germany) followed by size-exclusion chromatography at a low flow rate using two sequentially connected HiLoad 26/600 Superdex 200 pg columns (Cytiva) equilibrated in 10 mM Tris pH 8, 150 mM NaCl to increase the separation capacity. Fractions corresponding to the second peak containing the purest sc-GP2GP4 protein were pooled and concentrated. Of note, these fractions also contained two *Drosophila melanogaster* contaminants (identified by N-terminal sequencing using Edman degradation) migrating at ~35 kDa and 100 kDa (Fig. 1). In a similar manner, scFvs were purified from the cell culture supernatant using prepacked StrepTrap XT 1 ml columns (Cytiva) followed by SEC using a Superdex 200 Increase 10/300 GL (Cytiva) equilibrated in 10 mM Tris pH 8, 150 mM NaCl. Monomeric scFv peak fractions were used in subsequent experiments.

For production of mAbs, synthetic genes for heavy- and light-chain variable regions of antibodies were cloned into two separate pcDNA3.1 expression vectors under the control of a CMV promoter. All mAbs were expressed in Expi293F cells as IgG1. For this purpose, Expi293F cells were transfected using ExpiFectamine 293 transfection reagent (Gibco) following the manufacturer’s recommendation with minor changes. Briefly, a plasmid-DNA cocktail containing the heavy- and light-chain expression plasmids, and plasmids encoding the large T antigen of the SV40 virus, as well as the cell cycle inhibitors p21 and p27, was prepared in 5 ml Opti-MEM (Gibco) at a concentration of 1 µg per ml of final culture volume. The ExpiFectamine 293 transfection reagent was diluted in 5 ml Opti-MEM and mixed with the plasmid DNA cocktail after a 5 min incubation at room temperature. The mixture was then incubated for 30 min at room temperature, followed by dropwise addition of the mixture to the cells. Enhancers were added after 16 h. Cells were pelleted 6 days post-transfection, and IgG was purified by affinity chromatography from the supernatant using a HiTrap Protein G HP 1 ml column (Cytiva) followed by SEC using a Superdex 200 increase 10/300 column (Cytiva) equilibrated with PBS.

### Purification and inactivation of PRRSV particles

PRRSV type 2 strain VR2332 (GenBank: EF536003.1) was cultured in MARC-145 cells [38]. The virus-containing supernatant was harvested after cytopathic effects had affected more than 50% of the cells, clarified by centrifugation (4,500 ***g***, 15 min, 4 °C) and concentrated via ultracentrifugation through a 20% sucrose cushion (30,000 r.p.m., 3 h, 4 °C). This virus pellet was re-suspended in PBS overnight, loaded on top of a CsCl-PBS gradient of 1.0–1.4 g ml^−1^ and focused by ultracentrifugation (35,000 r.p.m., 12 h, 4 °C). A visible band at 1.18–1.21 g ml^−1^ (refractive index 1.35–1.345) was collected, diluted in PBS and again pelleted by ultracentrifugation (30,000 r.p.m., 1 h, 4 °C). Purified virions were re-suspended in PBS and inactivated with *β*-propiolactone at a final concentration of 0.05% for 1 h at 37 °C. The effectiveness of the purification was confirmed by infectivity tests of individual fractions, antigen ELISA against the N-protein of PRRSV and Western blot analyses.

### Immunization

Three BALB/c mice were immunized (Davids Biotechnologie GmbH, Regensburg, Germany) five times at 14-day intervals with a dose containing the equivalent amount of 10^9^ p.f.u. each (p.f.u. were determined prior to virus inactivation). The reactivity of all immunized mice was confirmed via ELISA and WB against viral proteins and the recombinant scGP2GP4 antigen preparation (Fig. S1, available in the online Supplementary Material). Finally, the mice were boosted with the recombinant sc-GP2GP4 for three consecutive days to direct reactive B cells into the spleen. Following euthanasia, spleen cells and peripheral blood mononuclear cells (PBMCs) were harvested, and final serum samples were collected as positive controls.

### Single-cell sorting of murine PBMCs

To isolate memory B cells recognizing PRRSV GP2 or GP4 from splenocytes, we used a two-step protocol: B cells were enriched from splenocytes of two immunized mice by magnetic cell separation using CD19 mouse microbeads (Miltenyi Biotec). Subsequently, the Fc receptors were blocked with anti-mouse CD16/CD32 antibody (clone 93), and antigen-specific cells were enriched by flow cytometric cell sorting using a panel of antibodies (fluorophores and antibody clones noted in brackets) targeting mouse CD4 (eFluor 450, clone RM4-5), CD8 (eFluor 450, clone 53–6.7), F4/80 (eFluor 450, clone BM8), Gr-1 (eFluor 450, clone RB6-8C5), B220 (APC, clone RA3-6B2), CD38 (Alexa Fluor 700, clone 90) and the Strep-tag (Alexa Fluor 488, clone C23.21) for detection of sc-GP2GP4 (100 µg ml^−1^). The antibody targeting the Strep-tag was purchased from Merck in an unlabeled form and labelled in-house. All other antibodies except anti-mouse IgG1 (BD Biosciences) were purchased from eBioscience. All staining procedures were performed in 50 µl per 10^6^ cells. Dead cells were excluded by 4′,6-diamidino-2-phenylindole (DAPI) staining. DAPI-, CD4-, CD8-, F4/80-, Gr-1-, CD19+, B220+, CD38+ and sc-GP2GP4+ cells were sorted using a FACSAria III Fusion (BD Biosciences) and directly processed with the Chromium Next GEM Single Cell V(D)J Reagent Kits v1.1. Sequencing of the scBCR libraries was performed on a MiSeq System (Illumina). The flow cytometry data were analysed using the FlowJo software, while the sequencing data were analysed with the Loupe V(D)J Browser, and productive sequences were reannotated with IMGT/HighV-Quest.

### Evaluation of control sera

Positive and negative control sera from pigs were tested using a commercially available ELISA kit (PRRS X3, 99-40959; IDEXX GmbH, Kornwestheim, Germany) in accordance with the manufacturer’s recommendations. Results were read immediately after the stop solution was added using an ELISA reader at 650 nm.

For Western blot evaluation of control sera, purified PRRSV type 2 strain VR2332 virions were analysed by SDS-PAGE and blotted onto a nitrocellulose membrane. Both the negative control serum (S2019_35) and the positive control serum (S2018_19) were diluted 1 : 1000 in PBS with 0.05% (vol/vol) Tween-20, and the reactivity was detected with goat anti-swine conjugate [Goat IgG anti-Swine IgG (H+L)-HRPO, Dianova, #ROC-614–1302] diluted 1 : 10,000 in PBS with 0.05% (vol/vol) Tween-20. ECL-Prime substrate was applied as a chemiluminescence reagent, and photon emission was recorded using an imaging system. A size standard is shown on the left for comparison (PageRuler pre-stained protein ladder, 10 to 180 kDa).

### Virus neutralization assay

To determine the neutralization potency of individual antibody fragments or intact antibodies, 100 µg of purified scFvs or IgGs was mixed with 100 TCID_50_ of the respective PRRSV strain in 200 µl serum-free medium. This mixture was incubated for 3 h at 37 °C and subsequently added to a 6-well dish seeded with 10^5^ MARC-145 cells the day before. Cells were incubated for 1 h at 37 °C with the neutralization mixture, washed twice with PBS and cultured in fresh medium for 24–48 h. Following fixation with 4% PFA and permeabilization with Triton X-100, infection was analysed via immunofluorescence staining using the N-protein-specific monoclonal *α*-PRRSV-N antibody 810. IC_50_ values were calculated using the GraphPad Prism 10 nonlinear regression feature with the log(agonist) versus response – variable slope (four parameters) equation.

### Indirect immunofluorescence assays

Indirect immunofluorescence assays were performed as previously described [39]. In brief, 1×10^6^ MARC-145 cells were seeded onto 6-well plates and infected with PRRSV-2 (strain VR2332) or PRRSV-1 (strain Lelystad, Ref-SKU: 026 V-04389 EVAg) or left untreated as controls (mock infection). After 24 h of incubation, the cells were fixed with 4% paraformaldehyde for 20 min at 4 °C, permeabilized with 0.5% (vol/vol) Triton X-100 (Merck, Darmstadt, Germany) in PBS and stained with the monoclonal antibody 810 anti-PRRSV-N or IgG #18 anti-PRRSV GP2-GP4. Goat anti-mouse IgG conjugated with Cy3 (Dianova, Hamburg, Germany) was used for detection. Cell nuclei were counterstained with DAPI (Thermo Fisher Scientific; Waltham, MA, USA) at a concentration of 1 µg ml^−1^ for 5 min at room temperature. Signals were evaluated using a sensitive fluorescence microscope (IX70; Olympus; Tokyo, Japan), and photomicrographs were captured with a monochromatic camera (DFC3000G; Leica; Wetzlar, Germany). All images presented in a single figure were acquired from the same experiment, following identical staining procedures and identical microscope settings.

### Western blot analysis

Infected and mock-infected cells were harvested at 24 h post-infection. Cell lysates and protein preparations were incubated at 95 °C for 5 min in SDS-PAGE loading buffer, separated in 7.5% (wt/vol) polyacrylamide tricine gels and transferred onto nitrocellulose membranes (Pall, Pensacola, Florida, USA). The membranes were blocked with 5% (wt/vol) skim milk (Carl Roth, Karlsruhe, Germany) in PBS with 0.05% (vol/vol) Tween-20 (Invitrogen, Karlsruhe, Germany). Murine sera and mAbs were used for antigen detection as indicated. Reactivity was visualized with the help of peroxidase-coupled secondary antibodies against murine IgGs (Dianova). ECL-Prime substrate was applied as a chemiluminescence reagent (GE HealthCare, Chicago, IL, USA), and photon emission was recorded with an imaging system (Odyssey M; LICOR, Lincoln, NE, USA).

### ELISA on sera from immunized mice

The proteins of CsCl gradient-purified PRRSV virions were dissolved by the addition of 1% Triton X-100 (Carl Roth). The sc-GP2GP4 antigen was dissolved in ELISA coating buffer (0.1 sodium carbonate, pH 9.5) and diluted to a final concentration of 10 µg ml^−1^. Viral proteins were diluted to a final concentration corresponding to 1×10^8^ p.f.u. ml^−1^ (infectious titre was determined before adding the detergent). ELISA 96-well plates (Maxisorb; Nunc, Thermo Fisher) were coated with 100 µl of antigen solution overnight and blocked using a commercial blocking reagent (ROTI-Block, Carl Roth) for 1 h at RT. The microwells were washed twice with 300 µl PBSt per well with thorough aspiration of microwell contents between washes. The buffer was allowed to sit in the wells for about 20 s before aspiration. Then, the serum samples were diluted and pipetted in the microwells in triplicates, including controls. After an incubation period of 1 h at room temperature (RT), the microwells were washed as described above. After an additional wash step, the wells were emptied and tapped on absorbent paper towel to remove excess PBSt. Fifty microlitres of diluted HRP-goat anti-mouse conjugate were added to all wells (1 mg ml^−1^ diluted 1/5,000 in PBSt). The microwells were covered with an adhesive film and incubated at RT for 1 h. Thereafter, microwells were washed four times with PBSt and a last time with PBS. Immediately after washing, 100 µl of 3,3′,5,5′-tetramethylbenzidine (TMB) substrate solution was pipetted to all wells. The microwell plate was incubated at RT for 15 min, before the substrate reaction was stopped by quickly pipetting 100 µl of ELISA-stop solution into each well. Results were read immediately after the stop solution was added using an ELISA reader at 450 nm as the primary and 630 nm as the reference wavelength.

### Expression and purification of MBP-peptide

The peptides mentioned in this paper were individually fused to the N-terminus of MBP by expression in the context of the plasmid pMAL-pIIIL (New England Biolabs), with MBP alone serving as control. All proteins were expressed in BL21 GOLD (DE3) and purified from the periplasm using an initial amylose affinity chromatography step followed by size exclusion using a Superdex 200 10/300 Increase column (Cytiva) equilibrated in PBS [40]. The fractions corresponding to monomeric protein were pooled and concentrated using 10 kDa centricon (Amicon, Millipore) to ~1 mg ml^−1^ and stored at −80 ℃ for ELISA.

### MBP-ELISA

Nunc MaxiSorp flat-bottom 96-well immunoassay plates (Invitrogen) were coated with 50 µl of MBP-peptides in varying concentrations, with 50 ng EK-cleaved sc-GP2GP4 antigen in native form, or with 50 µl of MBP-mimotope at 3 µg ml^−1^ (QPLSLLGEWARS; identified in the peptide phage display) serving as positive controls at 4 ℃ for 16 h. Plates were washed six times with PBS-Tween [1x PBS, 0.1% (v/v) Tween 20], blocked with 5% skimmed milk (prepared in PBS-Tween) for 1 h at 20 ℃ and washed thrice with PBS-Tween. Then, 75 ng scFv #18 in 5% milk was added per well and incubated for 1 h at 20 ℃ followed by four washes with PBS-Tween and addition of horseradish peroxidase-conjugated anti-mouse antibody in 5% skimmed milk for 45 min at 20 ℃. After four washes with PBS-Tween, the reaction was developed by adding TMB substrate (Biolegend) for 10 min at room temperature. The reaction was stopped by the addition of 1 M H_3_PO_4_, and absorbance was measured at 450 nm with 630 nm as reference using the ELx808 absorbance plate reader (BioTek). Data were analysed using GraphPad Prism 9. All ELISA assays were performed as three biological replicates with technical triplicates.

### Peptides and complex formation

The Strep-Tag of scFv #18 was cleaved using EKMax EK (Invitrogen), following the manufacturer’s recommendation, and subsequently purified using a reverse StrepTactin affinity chromatography followed by size-exclusion chromatography on Superdex 200 columns (HiLoad 26/600 or Increase 10/300) equilibrated in 10 mM Tris pH 8, 150 mM NaCl. The purified cleaved scFv #18 was concentrated to 20 mg ml^−1^. A synthetic peptide comprising residues 59 to 69 (ASEAIRKIPQC) of PRRSV-2 VR-2332 GP4 was synthesized by ProteoGenix SAS (>98% purity) and dissolved in DMSO at 100 mg ml^−1^. A complex was formed by mixing a final concentration of 4.8 mg ml^−1^ GP4 peptide with 13.2 mg ml^−1^ scFv (corresponding to a 1 : 10 molar ratio of scFv #18 : peptide) and incubating for 1 h at 277 K.

### Crystallization of scFv #18 in complex with its epitope peptide

Complex crystals were grown at 293 K by using the sitting-drop vapour diffusion method with drops containing 150 nl protein complex solution (18 mg ml^−1^ in 10 mM Tris pH 8.0, 150 mM NaCl) mixed with 150 nl reservoir solution. This reservoir solution contained 20% w/v polyethylene glycol monomethyl ether 5000 and 200 mM lithium acetate. Diffraction-quality crystals appeared after several weeks and were flash-frozen in mother liquor with 22% glycerol. Space groups and cell dimensions of the crystals, the number of molecules/complexes per asymmetric unit, resolution limits, data collection details and refinement statistics are summarized in Table S1.

Data were collected on the beamline PX-I at the Swiss Light Source (scFv alone) or on Proxima-1 at Synchrotron Soleil, processed, scaled and reduced by using XDS [41], Pointless [42] and programs from the CCP4 suite [43]. The crystal structure was determined by the molecular replacement method using Phaser [44]. We used separate variable and constant regions of a hypothetical scFv assembled from the best sequence match in the Protein Data Bank (PDB), the light chain (LC) reported under PDB accession number 5KVD and the heavy chain (HC) reported under PDB accession number 3WIH, as a search model for the scFv alone and the refined scFv #18 structure as a search model for the complex structure. Model building was performed by using Coot [45], and refinement was done by using AutoBuster [46]. Validation was carried out using MolProbity [47].

### Crystal structure analysis

Buried solvent-accessible surface areas for the interfaces and for individual residues within the peptides, as well as interactions, were calculated by using the PISA server [48]. Shape complementarity was calculated by using programs of the CCP4 suite [43]. Figures were prepared with PyMOL (The PyMOL Molecular Graphics System, Version 2.5.2, Schrödinger, LLC).

### Surface plasmon resonance analysis

Surface plasmon resonance (SPR) was performed to measure the binding affinity of the scFv (#18) to sc-GP2GP4 and a synthetic epitope peptide (AC11, ASEAIRKIPQC). For this purpose, Strep-Tactin^®^ XT (IBA GmbH) was amine-coupled to a carboxyl-activated CM5 sensor chip using the Biacore 3000 from GE Healthcare, following the protocol from the Twin-Strep Tag Capture Kit for SPR. Strep-Tactin^®^ XT was immobilized, resulting in a density of ~3,700 resonance units (RUs).

For kinetic measurements, Twin-Strep-tagged scFv #18 (27.6 kDa) in 1x HBS-EP buffer (10 mM HEPES, 0.15 M NaCl, 0.05% Tween-20, pH 7.4) was injected over flowcell 2 (FC2) surface for 30 s at a flow rate of 10 µl min^−1^. This achieved an immobilization density (Rimmo) of 200 to 400 RUs, followed by a 2 min stabilization period. For analysis of the interaction with peptides, an immobilization density (Rimmo) of 1,400 to 1,700 RUs was achieved. A synthetic peptide comprising the epitope II from the hepatitis C virus glycoprotein E2 (H77, NTGWLAGLFYQHK) and an unrelated glycoprotein served as negative controls, respectively.

sc-GP2GP4 (37 kDa) and the control glycoprotein (50 kDa) prepared in 1x HBS-EP buffer were injected over both FC2 and FC1 (with FC1 used for background correction) in a series of twofold dilutions ranging from 12.5 to 400 nM at a flow rate of 30 µl min^−1^ for 3 min at 25 °C. Peptides AC11 (1.2 kDa) and H77 (1.4 kDa) prepared in 1x modified HBS-EP buffer were injected over both FC4 and FC3 (with FC3 used for background correction) in a series of twofold dilutions ranging from 0.15 to 25 nM at a flow rate of 30 µl min^−1^ for 4 min at 25 °C. Experiments were performed three times. Dissociation was monitored for 4 min following injection. To regenerate the sensor surface, a 15 s wash with 3 M guanidine hydrochloride was performed. Sensorgram data were processed using BIAevaluation 3.0 software, with double-referencing applied using the reference channel and the zero-concentration response units. The association rate (*k_on_*), dissociation rate (*k_off_*) and equilibrium dissociation constant (*K_d_*) were calculated by globally fitting the experimental curves to a 1 : 1 Langmuir model, accounting for mass transfer effects.

### Enrichment of random peptide phage display libraries using IgG #18

A commercially available 12-mer, M13 gene 3-based random peptide phage display library (PhD-12, New England Biolabs, Hitchin, Hertfordshire, UK) was enriched for IgG #18-specific peptides by three rounds of affinity selection according to the manufacturer’s instructions. Briefly, IgG #18 (5 µg ml^−1^) was coated on a Nunc Maxisorp 96-well plate overnight in carbonate–bicarbonate buffer (Sigma) at 4 °C. Following blocking with 3% BSA for 2 h, a 10 µl preparation of phage library diluted in 90 µl TBS/0.05% Tween 20 (TBST) was added for 1 h. The well was washed ten times with TBST, and bound phage were eluted by treating with 90 µl Tris/glycine buffer (pH 2.8) for 10 min, followed by neutralization with 10 µl Tris/HCl buffer. Enriched phage were propagated by infecting a log-phase culture of *E. coli* strain ER2738 overnight, followed by double PEG 8000 precipitation and titration using a plaque assay. Enrichment was performed reiteratively three times before sequencing using Illumina Next-Generation Sequencing (NGS), alignment of sequences using Bowtie 2 and generation of a consensus sequence from the random peptide inserts. The enriched phage pool was titrated, and 20 individual clones were sequenced. These representative sequences were prepared as individual phage clone concentrates, and binding to the IgG #18 was assessed by ELISA [49]. An anti-tetanus toxin mAb [50] was used as an IgG1 isotype control.

### Generation of sequence conservation logos

Amino acid sequences representing the identified GP4 regions for PRRSV-1 and PRRSV-2 strains were retrieved from GenBank (https://www.ncbi.nlm.nih.gov/genbank/), and sequence conservation was created using Skylign (www.skylign.org).

## Results

### Production of a single-chain soluble GP2-GP4 (sc-GP2GP4)

To obtain a native-like secreted GP2-GP4 for structural and functional studies, we designed a single-chain GP2-GP4 construct (sc-GP2GP4) based on the PRRSV-2 sequence (ATCC VR-2332), as reported for other viral glycoprotein heterodimers [51,52]. In sc-GP2GP4, the transmembrane domain (TMD) of GP2 is replaced by a 19-amino-acid-long, flexible (GlySer) linker region, and the TMD of GP4 is replaced by a DST allowing for efficient affinity chromatography (Fig. 1a, b). In parallel, an analogous construct containing a C-terminal mNeonGreen fluorophore (sc-GP2GP4-mNeonGreen) was generated for use as bait to isolate glycoprotein-specific memory B cells, and stable *Drosophila* S2 cell lines were generated for both constructs. Induction of the metallothionein promoter resulted in the accumulation of secreted sc-GP2GP4 in the cell culture medium. We purified the protein from the supernatant as described in Methods, and SDS-PAGE analysis of the purified sc-GP2GP4 revealed two bands of unknown origin (Fig. 1c, red asterisks) migrating at ~35 and 100 kDa.

These two proteins were identified as *D. melanogaster* proteins, as confirmed by N-terminal sequencing, and could not be removed by additional chromatography steps, giving rise to a partially purified sc-GP2GP4/sc-GP2GP4-mNeonGreen.

### Isolation of nAbs targeting minor PRRSV glycoproteins

**Fig. 2.**
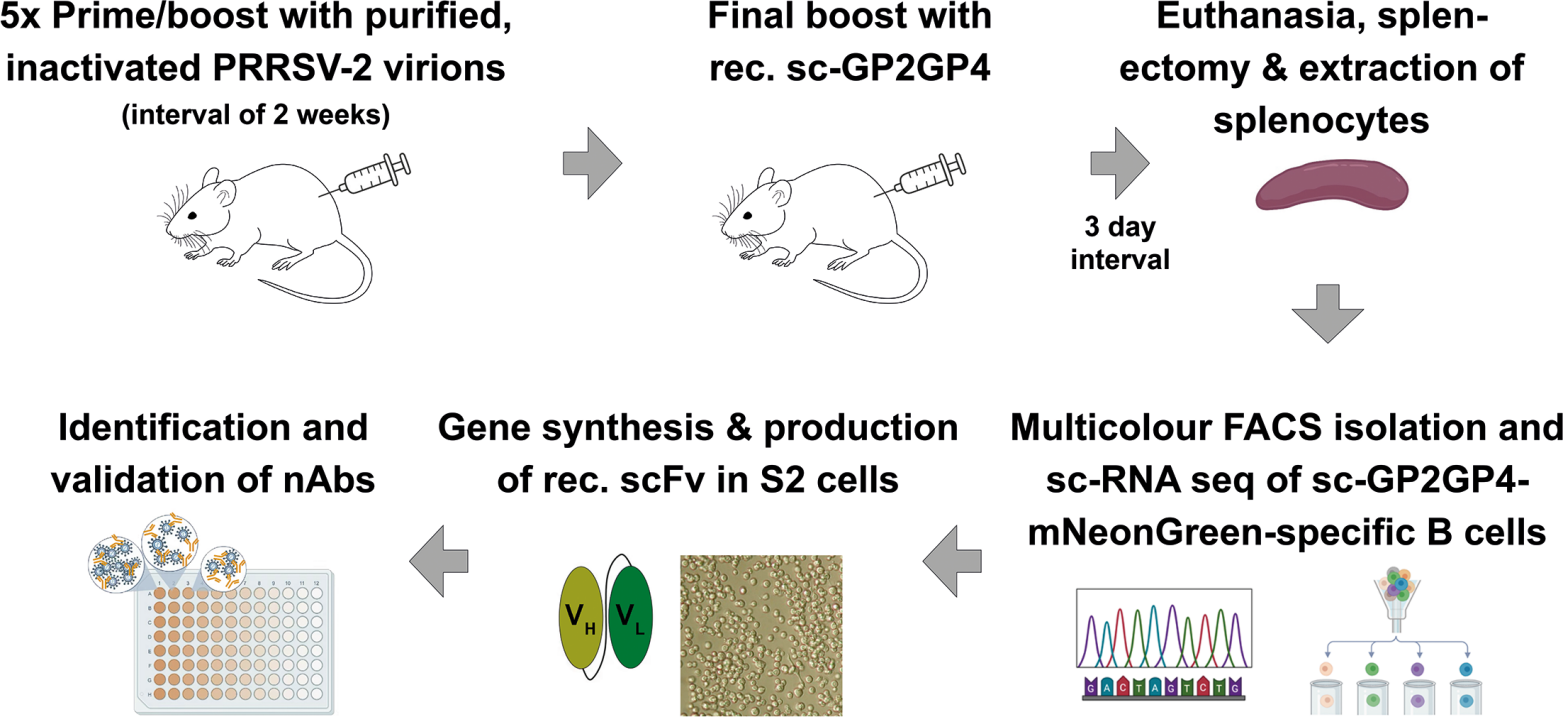
Immunization strategy and workflow for antibody isolation and characterization. Mice were immunized five times with purified, inactivated PRRSV-2 virions at 2-week intervals, followed by a final boost with recombinant sc-GP2GP4. Splenocytes were harvested 3 days later, and sc-GP2GP4-mNeonGreen-specific B cells were isolated by multicolour FACS and subjected to single-cell RNA sequencing. Recovered immunoglobulin genes were synthesized, expressed as recombinant scFv in *Drosophila* S2 cells and screened for neutralizing activity.

To identify neutralizing antibodies targeting the minor PRRSV glycoproteins GP2 and GP4, we immunized mice with PRRSV virions inactivated with *β*-propiolactone and purified as described in Methods. Sera reactivity with PRRSV and/or sc-GP2GP4 antigen was confirmed by ELISA (native state) and Western blot (denatured state) (Fig. S1). Using an established pipeline (Fig. 2), single PRRSV-reactive memory B cells were isolated from the pooled PBMCs from all immunized mice using magnetic-activated cell sorting followed by flow cytometry (see Methods; Fig. S2). Live sc-GP2GP4-mNeonGreen-binding CD19+, B220+ and CD38+ cells were subjected to single cell sequencing, yielding a total of 91 productive antibody sequences, including multiple IgM sequences and 19 IgG sequences. We cloned 35 heavy- and light-chain paired sequences (16 IgM and 19 IgG) into the context of intact scFvs and produced them in *Drosophila* S2 cells as described previously (Fig. 2) [36].

**Fig. 3.**
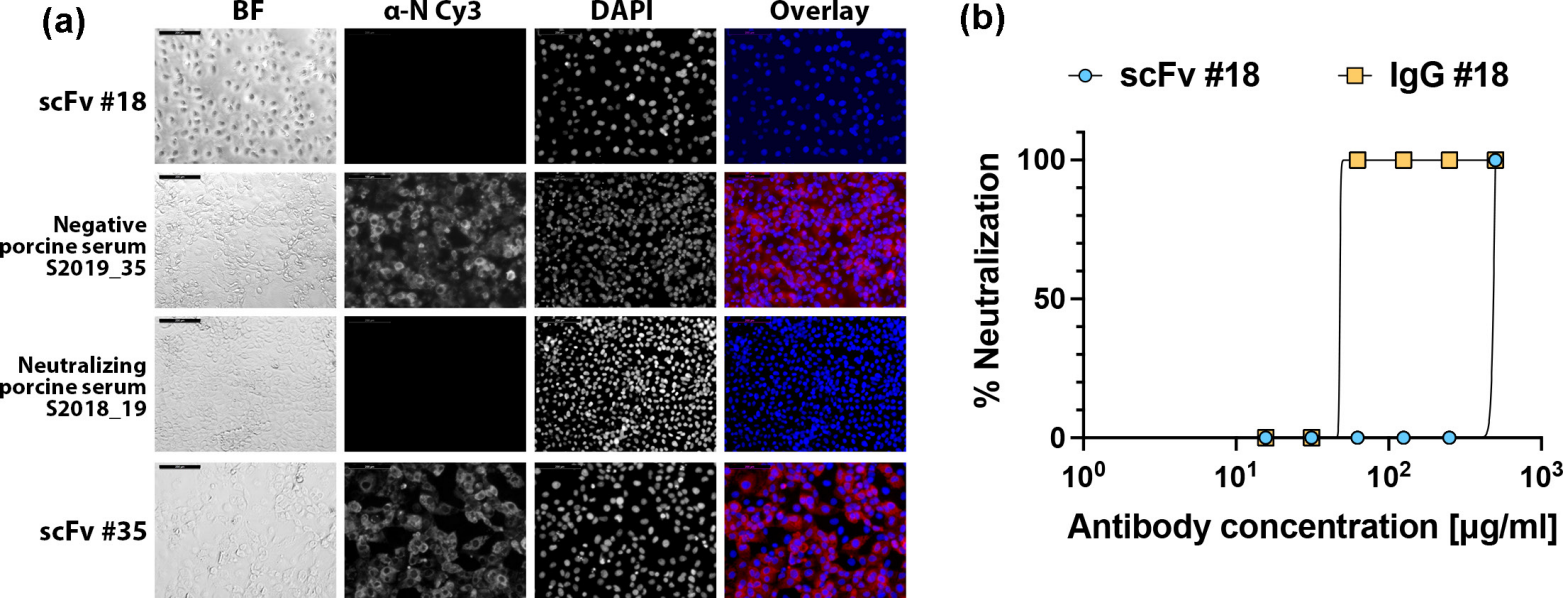
Neutralizing activity of scFv #18 and IgG #18. (a) PRRSV-2 VR-2332 was incubated with scFv #18 or scFv #35 (500 µg ml^−1^), as well as negative and neutralizing control sera (1:10 dilution in PBS) as described in Methods. Indirect immunofluorescence analysis of indicator cells using the *α*-PRRSV-N monoclonal antibody clone 810 (Cy3) revealed complete virus neutralization by scFv #18, but not by scFv #35. (b) 500 μg ml^−1^ scFv #18 was required to fully neutralize the virus. In contrast, IgG #18 showed stronger neutralization potency and fully blocked infection of the indicator cells at concentrations down to 62.5 µg ml^−1^, resulting in an IC_50_ of 498.8 µg ml^−1^ and 47.87 µg ml^−1^ for the scFv and IgG #18, respectively. All titrations were performed in triplicate of two biological replicates. The scale bar represents 200 μm.

We tested these scFvs for their ability to neutralize PRRSV type 2 VR-2332 on MARC-145 cells. Only scFv #18 neutralized VR-2332 at a level similar to the previously evaluated positive porcine control serum S2018_19, which specifically recognized PRRSV proteins in WB and a commercial ELISA (Fig. S3); the 50% inhibitory concentration (IC_50_) of scFv #18 was determined to be 498.8 µg ml^−1^ (Fig. 3). Subsequently, we assembled an intact murine IgG1 molecule based on the heavy- and light-chain variable regions of scFv #18 and expressed this in HEK293 cells. The neutralization potency of this IgG #18, with an IC_50_ of 47.87 µg ml^−1^, was markedly better than that observed for the scFv (Fig. 3b). Such a differential neutralization potency between full-length IgG antibodies and their corresponding antibody fragments has been reported previously for antiviral nAbs [53,54].

Evaluation of the neutralization breadth of IgG #18 on MARC-145 cells using three additional PRRSV-1 and PRRSV-2 strains (PRRSV-1 strain 613 [6], PRRSV-1 strain Flex [55] and PRRSV-2 strain pFL12 [56]) revealed a neutralizing activity of the antibody only for PRRSV-2 VR-2332, suggesting a strain-specific reactivity (Fig. S4).

### Characterization of IgG #18

Immunofluorescence analysis of cells infected with PRRSV type 2 VR-2332 using IgG #18 revealed a specific immunofluorescence staining. No signal was detected in uninfected MARC-145 cells used as control (Fig. S5), highlighting the specificity of the reaction. The staining in infected cells was cytoplasmic, excluding the nucleus, with circumnuclear enhancement, suggesting a localization of the GP2GP4 to membranes of the endoplasmic reticulum. Of note, no signal was detected in MARC-145 cells infected with PRRSV type 1 (strain Lelystad; Fig. S5), indicating that the epitope targeted by IgG #18 is not conserved between the strains of PRRSV-1 and PRRSV-2 used in these assays. Western blot analysis of lysates from VR-2332-infected MARC-145 cells revealed that IgG #18 recognized a protein migrating at ~38 kDa under non-reducing conditions, causing a faint band along with indistinct high-molecular-weight signals. Upon sample reduction, a distinct, strong band at ~38 kDa was observed, suggesting that IgG #18 requires denaturation and reduction of the target glycoprotein for optimal antigen interaction (Fig. S6).

### Identification of the IgG #18 epitope

**Fig. 4.**
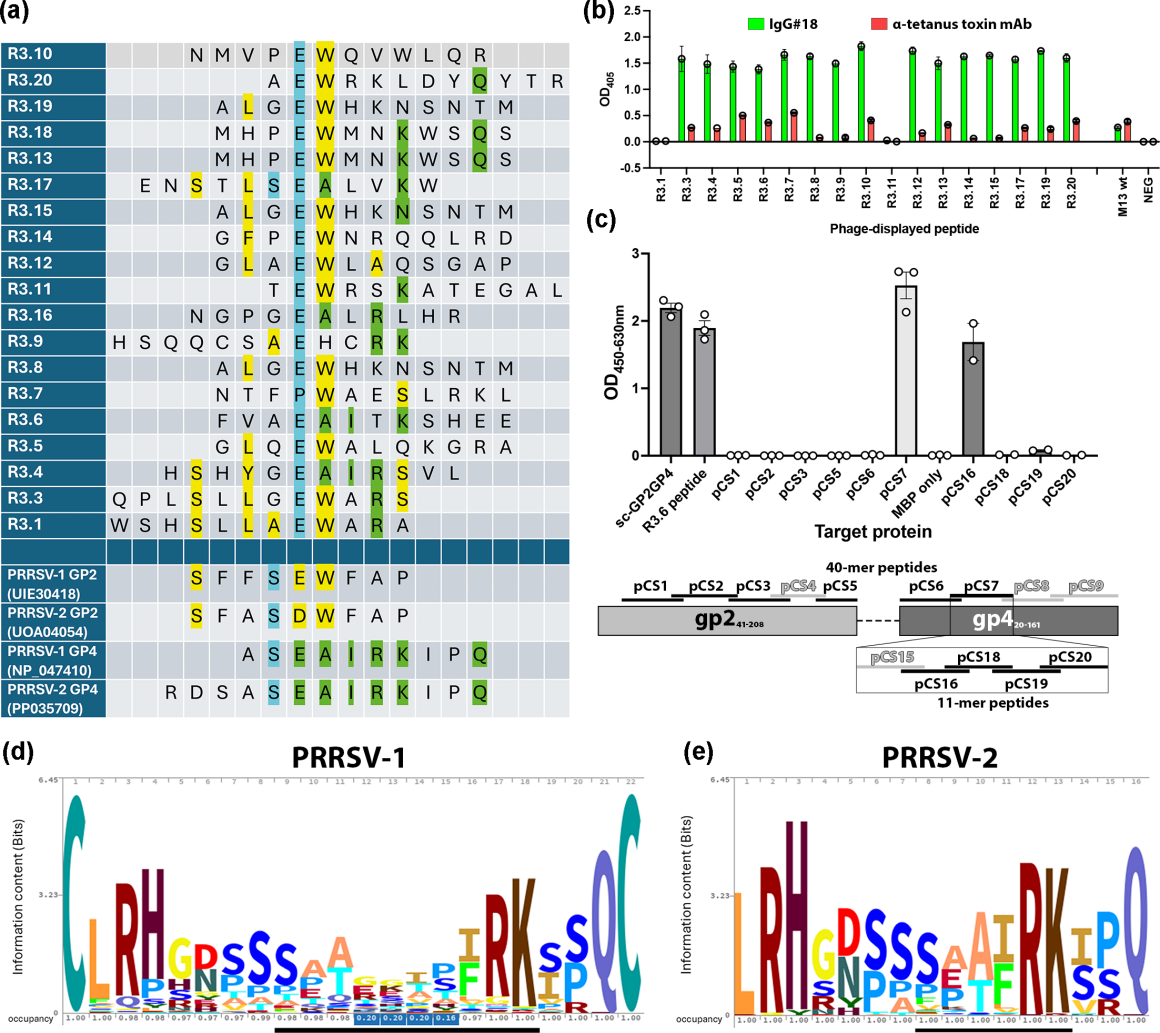
Epitope mapping of IgG #18. (a) Enrichment of a phage-displayed random peptide library (RPDL) identified 19 individual peptide sequences that were highly represented in the enriched library. Alignment and analysis using blast revealed potential epitope ‘hits’ in both GP2 and GP4. Amino acids aligning with a region in GP2 are highlighted in yellow, with matches to GP4 highlighted in green. The core glutamic acid present in all peptides that matched to both GP2 and GP4 is highlighted in turquoise, with residues matching GP2 highlighted in yellow and matches with GP4 highlighted in green. (b) Binding specificity for 17 individual phage clone preparations was confirmed by ELISA for binding to IgG #18 (green) using an *α*-tetanus toxin mAb as a control (red). An effective binding signal (OD405) was obtained by subtracting the optical density value of a control well from the value measured for each sample. (c) Binding of IgG #18 to MBP-peptide constructs representing PRRSV-2 VR-2332 GP2 and GP4 sequences. Individual peptides are shown in the bottom panel as black bars for peptides that have been experimentally tested and light grey bars for peptides that have been omitted. (d, e) Variability of the identified epitope region in PRRSV-1 (d) and PRRSV-2 (e) GP4 region. Weblogos generated with Skylign illustrate sequence variation in this region for PRRSV-1 and PRRSV-2 and highlight the extensive variability across PRRSV-1 strains and the general conservation of the SEAIRK motif in PRRSV-2 isolates. Below each Weblogo, the total amino acid frequencies are shown. An insertion in ~20% of the PRRSV-1 sequences is highlighted by blue boxes, and a black line indicates the region of the SEAIRK motif highlighted in panel (a).

We next set out to determine the epitope recognized by IgG #18. We initially enriched a random peptide phage display library using IgG #18 as described previously [57]. After three rounds of affinity selection against IgG #18, the enriched libraries were subjected to Illumina NGS (Genewiz), allowing for the identification of prominent key residues that contributed to interaction between IgG #18 and the PRRSV minor glycoproteins. Affinity selection identified a broad consensus motif, which, when compared to the protein sequence of GP2 and GP4, revealed two potential linear amino acid sequence targets for IgG #18, one in GP2 and one in GP4 (Fig. 4a).

Specific binding was identified for peptide sequences matching each of these two glycoprotein regions in both GP2 and GP4 (Fig. 4a, b). This result highlights two regions as possible epitopes of IgG #18, one present in GP2 and one in GP4. To extend the peptide phage display analysis and to identify the main interacting residues, we generated a library of MBP fusion proteins, each one containing a peptide corresponding to a part of the amino acid sequence of PRRSV-2 GP2 and GP4 present in sc-GP2GP4 fused to MBP via a short linker peptide. These MBP-peptide constructs were individually expressed, purified to homogeneity and tested in an ELISA to assess binding by IgG #18. In the initial screening using fusion proteins with large (~40 amino acids) peptides (pCS1–pCS9), only construct pCS7 was recognized by IgG #18, suggesting that the antibody specifically binds to an epitope in the N-terminal half of GP4. In a second step, we generated MBP-fusion constructs that contained ~11 amino acids spanning this peptide region (pCS15–pCS20). ELISA screening unambiguously identified a segment corresponding to GP4 residues 59–69 (ASEAIRKIPQC; GenBank accession ABB29768.1) as the epitope recognized by IgG #18 (Fig. 4c). Phylogenetic analysis of this segment revealed a pronounced variability (Fig. S7).

**Fig. 5.**
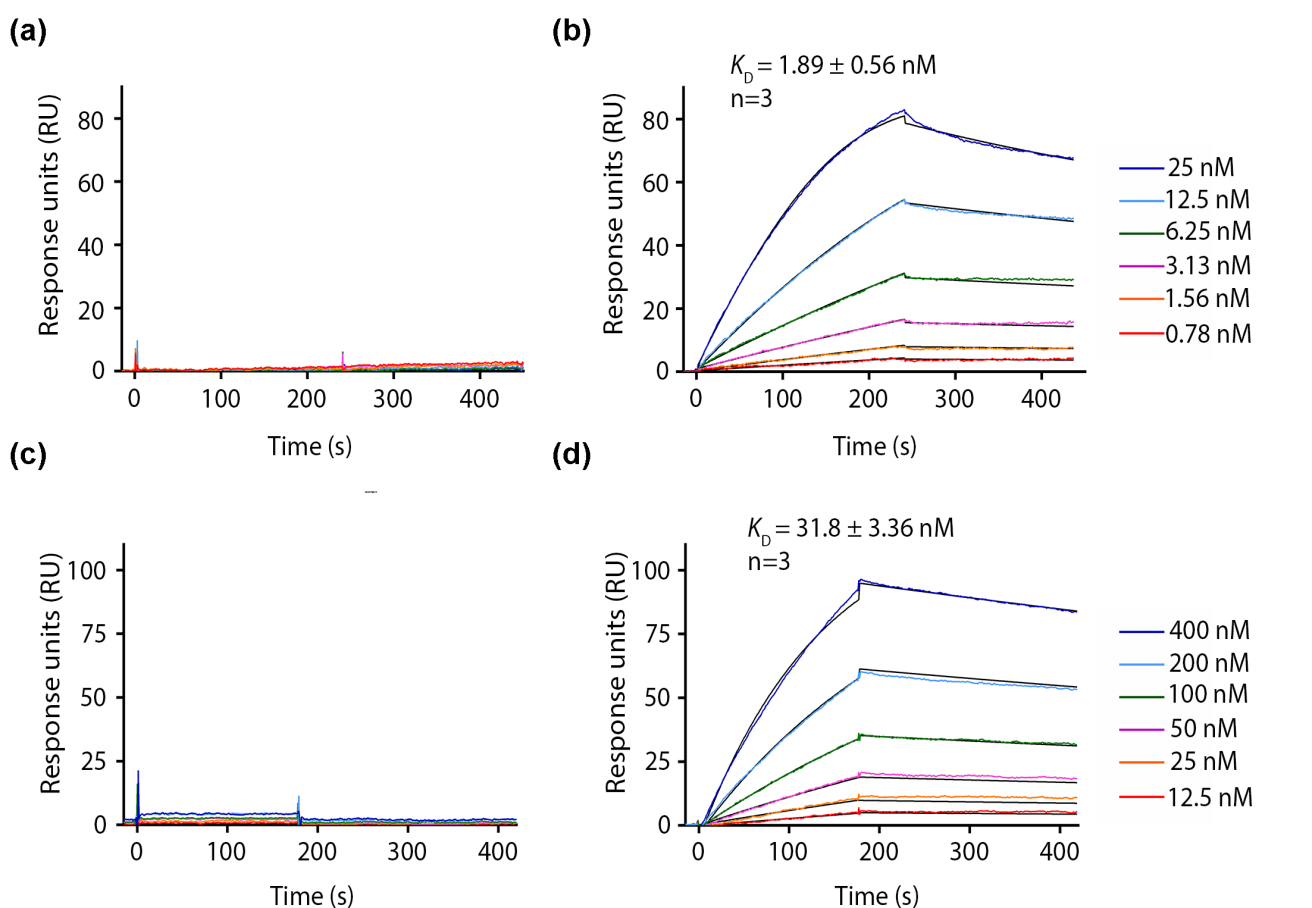
SPR analysis of scFv #18 binding to its epitope. (a, b) Real-time SPR analysis of the interaction between synthetic peptides or recombinant glycoproteins and scFv #18. A control peptide comprising epitope II of the hepatitis C virus strain H77 (a, NTGWLAGLFYQHK), the GP4 epitope peptide for scFv #18 (b, ASEAIRKIPQC; GP4 peptide), an unrelated glycoprotein (c) and the recombinant sc-GP2GP4 (d) were injected over a surface of immobilized scFv #18 captured on a Streptactin XT surface (see Methods) at a flow rate of 30 µl ml^−1^, and the binding response in RUs was recorded as a function of time. To account for the small signal, three independent experiments were performed, and the mean values with standard errors are presented in Table S2.

To further characterize the interaction between IgG #18 and its cognate antigen, we determined the equilibrium dissociation constants (*K_d_*), as well as association (*k_on_*) and dissociation (*k_off_*) rates for the interaction. Because a bivalent IgG molecule can exhibit higher apparent binding affinity due to avidity effects, we tested the binding of the corresponding monovalent scFv #18 to different antigens by SPR (Fig. 5). SPR analysis was performed using the recombinant sc-GP2GP4 in parallel with the GP4 epitope peptide of the PRRSV-2 VR-2332 sequence (GP4 peptide) to allow for a comparative analysis of kinetic binding parameters. Determined kinetic binding parameters are given in Table S2 and reveal high affinity interactions as expected for a potent neutralizing antibody. The recombinant sc-GP2GP4 bound to the immobilized scFv #18 with nanomolar affinity (*K_d_* of ~31.8 nM), in line with affinities of reported antibody–antigen interactions targeting linear epitopes [58]. Unexpectedly, the GP4 peptide bound to the scFv with ~16-fold higher affinity (*K_d_* of ~1.89 nM). While the dissociation constant is only slightly lower for sc-GP2GP4, an ~40× higher association constant is observed for peptide binding. Such a difference in kinetic binding parameters could result from either a non-native conformation or from limited accessibility of the epitope in the context of the recombinant sc-GP2GP4, e.g. due to glycan shielding or shielding by proteins from *D. melanogaster* that co-purified with the recombinant sc-GP2GP4.

### Structure of scFv #18 in complex with the GP4 peptide

**Fig. 6.**
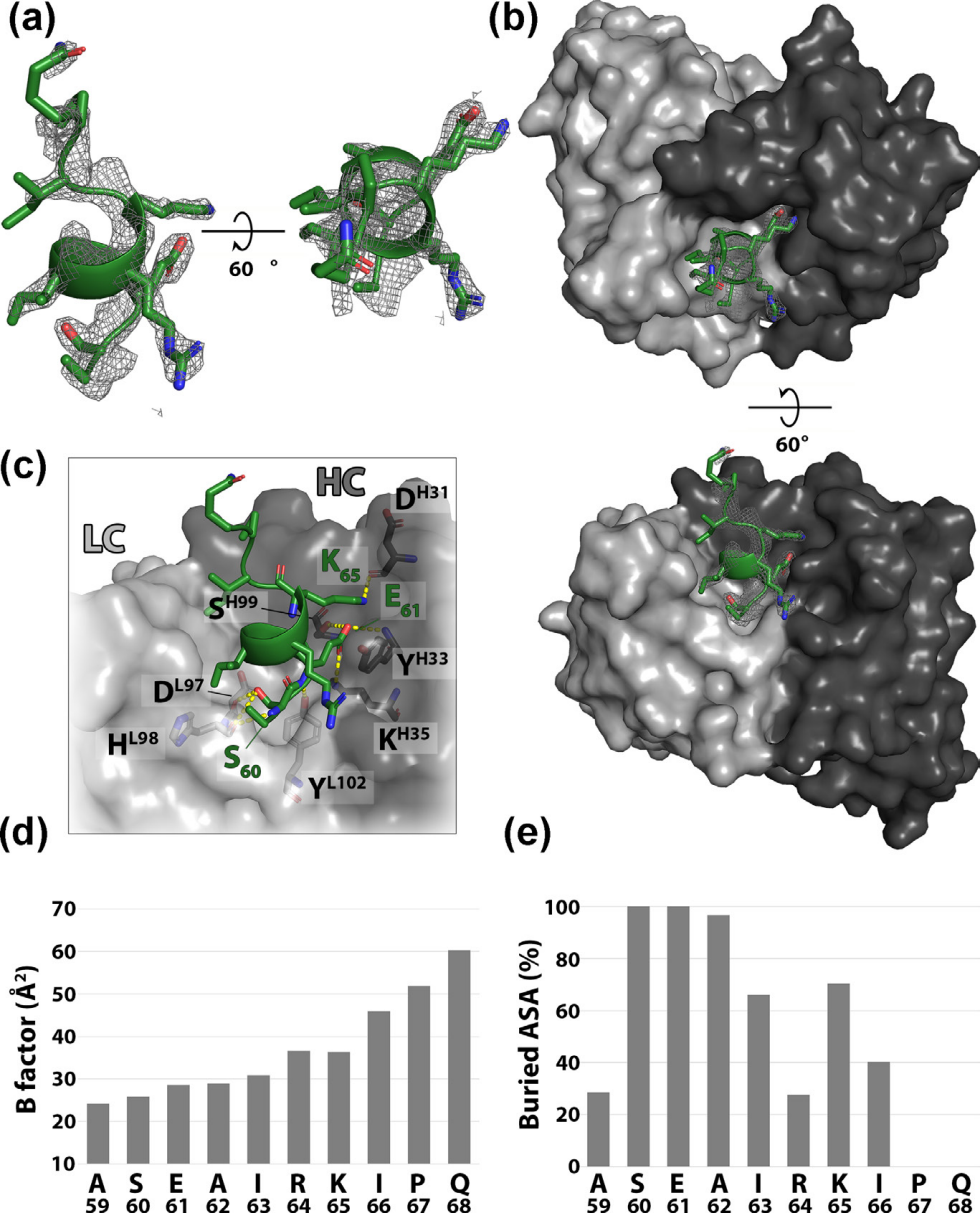
Crystal structure of scFv #18 in complex with a GP4 epitope peptide. (a, b) The structure of scFv #18 in complex with the GP4 peptide was determined and refined to 2.3 Å resolution and features unambiguous density for GP4 residues 59–68. The scFv #18 is shown as a molecular surface with the heavy and light chain coloured in dark and light grey, respectively. The peptide is shown as cartoon and coloured by atom type (green, red and blue for carbon, oxygen and nitrogen atoms, respectively). Electron density of a 2Fo-Fc map (grey mesh) is contoured at 2 σ, illustrating the quality of fit for the peptide. (c) Interactions between the GP4 peptide and the paratope are dominated by peptide residues S60, E61 and K65. Residues are labelled in green and black for the peptide and antibody, respectively. Yellow dashes indicate hydrogen bonds or the salt bridge. (d) Average temperature factors of the peptide plotted per residue, suggesting that the core interaction is located rather N-terminal. (e) Percentages of accessible surface area (ASA) buried in the complex, calculated using PISA represented per residue.

To gain insights into the structure of IgG #18, we performed crystallization experiments of scFv #18 alone. We obtained diffraction-quality crystals and determined their structure by the molecular replacement method using the variable and constant regions of unrelated Fab fragments as separate search models (see Methods). In parallel, to understand the interaction with its cognate antigen and its mode of antigen recognition, we co-crystallized a complex containing scFv #18 and the GP4 peptide and obtained diffraction-quality crystals. The structure of the scFv #18-peptide complex was determined by the molecular replacement method (Fig. 6) using the crystal structure of unliganded scFv #18 as search model. Details of the crystallization and structure determination are described in the Methods, and the crystallographic statistics are listed in Table S1. The final electron density map revealed unambiguous density for a single scFv-peptide complex per asymmetric unit, allowing us to build an atomic model for GP4 residues 59 to 68 (Fig. 6a–c). Of note, no clear density was observed for the C-terminal cysteine residue, which, in the context of an intact GP4, likely forms a disulphide bridge.

The GP4 peptide forms one *α*-helical turn spanning GP4 residues E^61^–R^64^ (Fig. 6a–c). The peptide interaction with the paratope buries an area of ~548 Å^2^ on the peptide and ~402 Å^2^ on the scFv, with a total buried surface area of 950 Å^2^ and a shape complementarity index of 0.795, similar to other antibody–antigen complexes [59,60]. The crystallographic temperature factor (B-factor) per residue analysis indicates a stable and strong interaction with the paratope, with a higher degree of disorder towards the peptide C-terminus (Fig. 6d). B-factors for I^66^–Q^68^ were at least ~10 Å^2^ higher than for N-terminal peptide residues (Fig. 6d). In line with the temperature factor distribution, an analysis of the buried surface area per residue also shows an interaction that is mainly mediated by the N-terminal part of the peptide, including the short *α*-helix described above (Fig. 6e). These analyses strongly suggest a highly ordered and stable antibody/antigen complex interface.

The interaction of GP4 with IgG #18 is dominated by the sidechains of E^61^ and K^65^, which deeply protrude into a cavity shaped by the antibody’s HC. The three major interacting GP4 residues S^60^, E^61^ and K^65^ interact with both the LC (S^60^) and the HC (E^61^ and K^65^). A hydrogen-bonding network with seven hydrogen bonds stabilizes the interaction between these three residues and the antibody residues D^H31^ and Y^H33^ in the CDRH1, S^H99^ in the CDRH3 and residues D^L97^, H^L98^ and Y^L102^ in the CDRL3 (Fig. 6c). Additionally, the interaction network is complemented by a salt bridge between E^61^ and K^H35^. Of note, five out of seven hydrogen bonds involve backbone atoms in the paratope, while specific binding appears to be primarily mediated by protrusion of E^61^ and K^65^ into the binding pocket formed by the antibody’s CDRH1 and CDRH3.

**Fig. 7.**
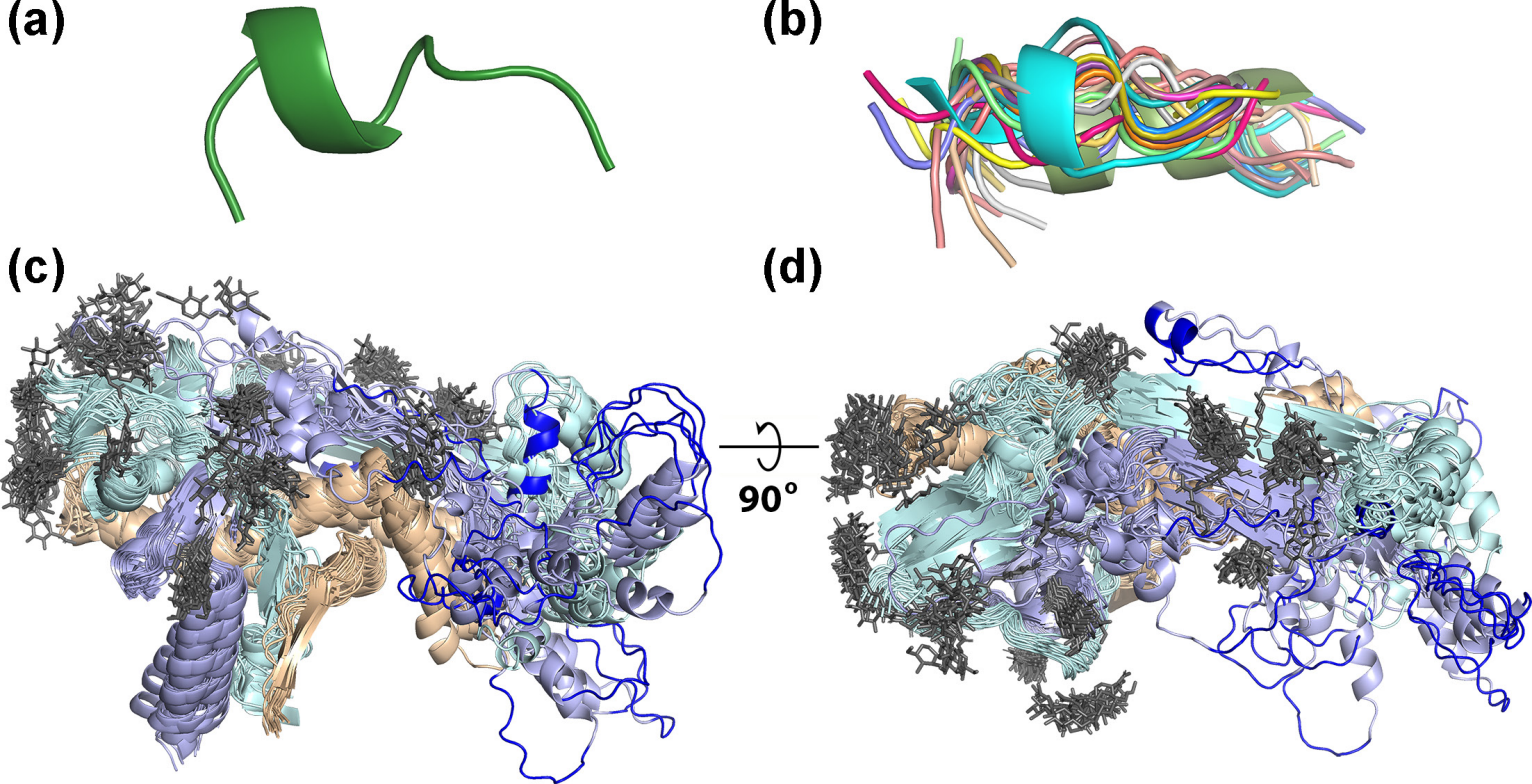
Conformation of the scFv #18 epitope in the context of the predicted structure of the minor glycoprotein trimer. (a) Cartoon view of the scFv #18 epitope peptide from the crystal structure. (b) Four independent AlphaFold 3 runs gave rise to 20 models of the PRRSV-2 VR-2332 minor glycoproteins. The amino acid segments corresponding to the peptide shown in (a) were extracted from the 20 models and structurally superposed, illustrating a poor quality of the prediction within this segment and a lack in superposition between most of the predicted conformations and the crystal structure. (c, d) Cartoon view of an ensemble of 20 predicted models with GP2, GP3 and GP4 coloured in wheat, light cyan and light blue, respectively. The segment corresponding to the scFv #18 epitope peptide is coloured in dark blue, and the N-terminal 55 residues of GP2 are not shown.

It is important to note that the PRRSV minor glycoproteins are believed to form a heterotrimer with ~436 residues in their ectodomains, corresponding to a glycoprotein spike of ~5–10 nm on the viral surface [61]. To interpret our structure in the native context, we predicted the structure of an intact trimer containing the ectodomains of GP2 (aa 1–180), GP3 (aa 1–150) and GP4 (aa 1–129) using AlphaFold 3 [62]. Four independent runs yielded low confidence scores (pTM and ipTM <0.5), suggesting a high uncertainty in global domain positioning, as well as unreliable or non-specific inter-chain interactions. Of note, both accuracy and confidence scores of AlphaFold predictions strongly depend on the depth and quality of a multiple sequence alignment (MSA), and viral proteins frequently have shallow MSAs and a limited number of homologues; therefore, reduced model confidence scores are not unexpected [63]. The predicted ectodomain trimer structures – in spite of the low confidence scores – displayed an unanticipated structural similarity in all 20 obtained models with an overall RMSD of 1–2 Å across >3,000 atoms (Fig. 7c, d), with the exception of the N-terminal 55 residues of GP2 displaying 2 helices that are predicted in multiple different conformations and are not shown in Fig. 7. All secondary-structure elements and the entire quaternary architecture of the heterotrimer are consistent across all 20 models.

Superposition of the predicted GP4 residues 59–69, corresponding to the crystallized scFv #18 epitope (Fig. 7b), revealed substantial conformational variability within this segment and no close structural match to the epitope conformation observed in the crystal structure (Fig. 7a). Although efficient neutralization by IgG #18 implies that an identical epitope conformation is accessible on the intact virion, our structure is limited to the antibody-bound epitope and does not resolve the broader conformational organization of GP4. The predicted pronounced heterogeneity of the scFv #18 epitope is consistent with a conformationally flexible or poorly constrained region, in line with the length polymorphism observed in this segment due to insertions in certain strains of PRRSV-1. In the AlphaFold 3 models, the scFv #18 epitope is positioned within an extended loop adjacent to a central three-stranded *β*-sheet composed of *β*-strands from both GP3 and GP4. The localization of the epitope within such an extended loop region also supports the notion of structural flexibility.

## Discussion

Accumulating information on neutralizing antibodies targeting the PRRSV glycoproteins illustrates the growing interest into how this virus is targeted by the humoral immune system. Nevertheless, no structural insights into the minor glycoproteins GP2, GP3 and GP4 have been reported, likely due to difficulties in the production of natively folded glycoproteins and the lack of a high-resolution cryo EM reconstruction of intact virions. Here, we present the identification and immunological, biochemical and structural characterization of the murine monoclonal IgG #18 targeting a linear epitope within GP4 that is immunogenic in pigs [24] and is thus also relevant in an *in vivo* context.

In indirect immunofluorescence, IgG #18 recognized GP4 in the context of MARC-145 cells infected with the North American PRRSV-2 VR-2332 strain. However, IgG #18 was not reactive with the GP4 of PRRSV-1 (strain Lelystad), suggesting a limited conservation of the epitope between the two PRRSV species. The targeted epitope region within GP4 has been described as the most variable region within GP4 and displays significant variability between strains representing PRRSV-1 and PRRSV-2 and even within these two virus species (Figs 4d, e and S7; sequence information underlying this analysis is compiled in supplementary data). The corresponding amino acid segment induces neutralizing antibodies in pigs that potently neutralize homologous, but not heterologous, PRRSVs [24,28, 64], in line with our results describing the limited neutralization breadth observed for IgG #18.

Neutralizing antibody responses against highly variable RNA viruses such as the human immunodeficiency virus (HIV), influenza virus or hepatitis C virus often face similar challenges of epitope diversity and glycan shielding (reviewed in [65,66]). For these pathogens, structural studies of broadly neutralizing antibodies have provided critical blueprints for immunogen design by revealing conserved epitopes that can be targeted across diverse strains. While the epitope targeted by IgG #18 is not conserved, its structural characterization nevertheless provides a defined molecular snapshot of strain-specific neutralization and highlights how antigenic variability restricts epitope conservation, underscoring the need to identify conserved structural determinants beyond variable regions such as GP4. Thus, insights from other viral systems may inform strategies to elicit broader immunity against PRRSV.

The neutralization mechanism of IgG #18 remains elusive to date. Of note, both GP2 and GP4 have been demonstrated to directly interact with the cellular receptor CD163 [67]. The observed sequence variability in the IgG #18 epitope, however, strongly argues against a direct implication of this segment in receptor binding, as receptor-binding motifs are usually highly conserved and antibodies target these conserved residues to avoid immune evasion [68]. Combined with the fact that it is likely in close proximity to the receptor-binding region, this sequence variability in the IgG #18 epitope rather suggests an indirect neutralization mechanism, e.g. steric hindrance between the bound nAb and the receptor. Of note, PRRSV epitopes eliciting strong antibody responses in pigs tend to be poorly conserved across a broad range of virus isolates [69], whereas conserved epitopes frequently provoke little to no immune response. Such immunodominant and highly immunogenic, variable epitopes are often located adjacent to epitopes that are more conserved and have essential functions, e.g. in receptor binding. This strategy effectively helps the virus to evade antibodies targeting conserved viral targets. The localization of the IgG #18 epitope within a highly variable segment of GP4 highlights a possible immune evasion mechanism employed by PRRSV. By focusing antibody responses on variable, strain-restricted epitopes, the virus may divert immunity away from functionally conserved regions that could confer broader protection. This observation emphasizes the need for vaccine approaches that redirect immunodominance towards conserved elements of the viral envelope complex.

Beyond GP4, other components of the PRRSV envelope complex (e.g. GP2, GP3 and GP5) are likely to harbour neutralization epitopes. Applying the integrative approach used in this study – combining monoclonal antibody isolation, functional assays and structural biology – could provide a systematic map of protective antibody targets. Such efforts would build a more comprehensive structural framework for rational vaccine design against PRRSV.

The protective effect of current PRRSV vaccines is often incomplete, resulting in reduced viraemia and clinical signs rather than in a complete sterilizing immunity. Cross-reactivity remains a major challenge, with most vaccines inducing only weak protection against genetically divergent strains. In a number of viral pathogens, where conventional vaccine design failed to produce efficient vaccines (e.g. HIV and respiratory syncytial virus), an epitope-based approach has induced excellent titres of neutralizing antibodies, some of which were even demonstrated to be protective in *in vivo* studies [32,33]. Although the epitope identified in this study is not conserved across PRRSV strains, the structural information on a neutralization epitope in complex with a neutralizing antibody fragment presented here offers a valuable blueprint for applying AI-based computational methods to rational immunogen design [70,71]. Advances in protein structure prediction and machine learning–driven design now enable the engineering of immunogens that stabilize conserved epitopes or mask variable decoy regions. Integrating such computational approaches with structural virology may accelerate the development of next-generation PRRSV vaccines capable of eliciting broadly protective immune responses. Future studies should therefore aim to structurally define conserved neutralization epitopes and assess whether structure-guided immunogen design can enhance vaccine efficacy against genetically diverse PRRSV strains. A key priority in this context will be to characterize the antibody response elicited by PRRSV glycoproteins in the natural host, for example, by applying single-cell B cell profiling technologies to samples from immunized and/or infected swine. Such analyses will provide a deeper understanding of the epitope landscape and antibody specificities that are relevant *in vivo*, thereby offering crucial insights for the rational design of broadly protective PRRSV vaccines.

## Supplementary material

10.1099/jgv.0.002284 Supplementary Material 1.

## Abbreviations

DAPI: 4′,6-diamidino-2-phenylindole
DST: double Strep tag
EK: enterokinase
FC2: flowcell 2
HC: heavy chain
HIV: human immunodeficiency virus
IC_50_: 50% inhibitory concentration
LC: light chain
MBP: maltose-binding protein
MLVs: modified live vaccines
MSA: multiple sequence alignment
nAbs: neutralizing antibodies
NGS: Next-Generation Sequencing
PBMCs: peripheral blood mononuclear cells
PDB: Protein Data Bank
PRR: porcine reproductive and respiratory syndrome
PRRSV: porcine reproductive and respiratory syndrome virus
RUs: resonance units
scFv: single-chain variable fragment
SPR: surface plasmon resonance
TMB: 3,3′,5,5′-tetramethylbenzidine
TMD: transmembrane domain
