# Supplementary material for: Structural basis of porcine reproductive and respiratory syndrome virus 2 neutralization by a GP4-targeting monoclonal antibody
Source: J Gen Virol. 2026 Jun 25;107(6):002284. doi: 10.1099/jgv.0.002284 (PMC13312592; doi:10.1099/jgv.0.002284)
Supplement: Supplementary Material 1. [file jgv-107-02284-s001.pdf]

# Structural Basis of Porcine Reproductive and Respiratory Syndrome Virus 2 Neutralization by a GP4-targeting monoclonal antibody

Barbora Veselkova[1,2], Chandamita Saikia[2], Sebastian Affeldt[3], Sandra Barth[3], Manoj Kumar Rajak[1,2], Frithjof Besa[2], Daria Bezbakh[2], Guido Hansen[2], Till Rümenapf[4], Matthias Ballmaier[5], Oliver Dittrich-Breiholz[6], Holly Bamber[7,8], Kumar Nagarathinam[2], Alexander W. Tarr[7,8], Benjamin Lamp[3], Thomas Krey[1,2,9,10,11]

**Table of Contents:**

**Figure S1:** **Reactivity of immune sera with PRRSV-2 and/or sc-GP2GP4 antigen**

**Figure S2:** **FACS plots showing the gating strategy for the sorting of single PRRSV-specific memory B cells**

**Figure S3:** **Validation of positive and negative control sera**

**Figure S4:** **Specificity of IgG#18 neutralizing activity**

**Figure S5:** **Immunofluorescence analysis using IgG #18**

**Figure S6:** **Western blot analysis using IgG #18**

**Figure S7:** **Phylogenetic analysis of the IgG #18 epitope sequence**

**Table S1:** **Diffraction data collection and refinement statistics**

**Table S2:** **Kinetic parameters of scFv #18 binding to sc-GP2GP4 and the epitope peptide**

**Supplementary data: Epitope variability in GP4 of PRRSV-1 and PRRSV-2**

**Figure S1: Reactivity of immune sera with PRRSV-2 and/or sc-GP2GP4 antigen**

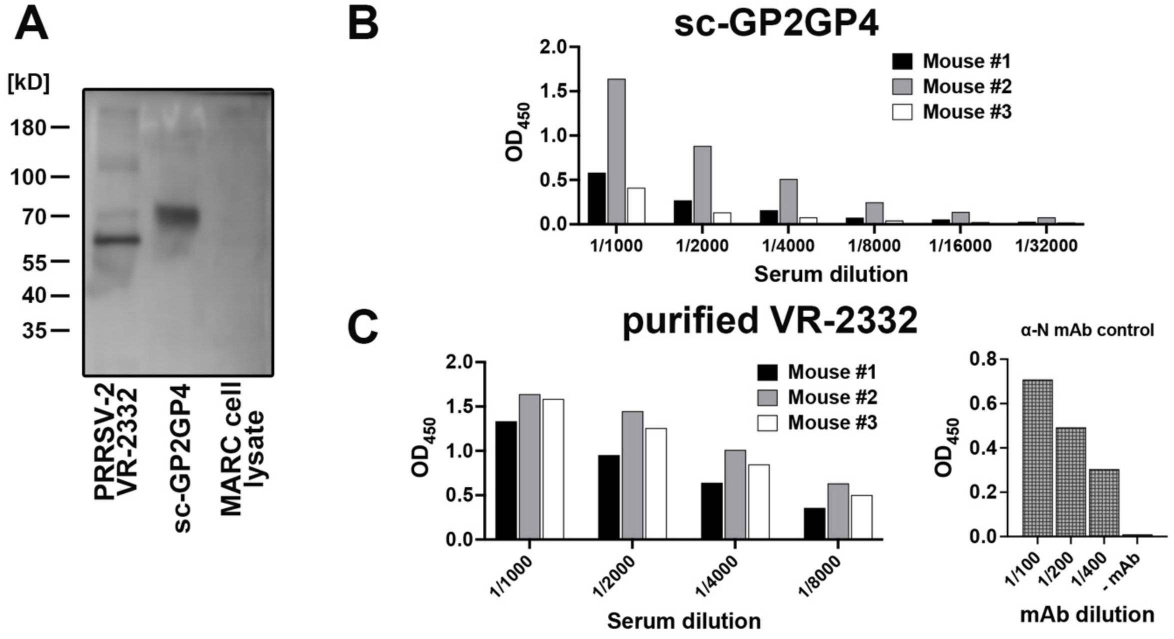


Figure S1: Immunization induced antibodies targeting the minor PRRSV glycoproteins GP2/GP4 in mice. Final bleed sera of three immunized mice were analyzed (A) by immunoblot against purified PRRSV-2 VR-2332, purified sc-GP2GP4 heterodimer, and lysate of non-infected MARC-145 cells used as control. Sera were also analyzed by ELISA against (B) purified sc-GP2GP4 heterodimer and (C) purified PRRSV-2 VR-2332. For the latter, a mAb targeting PRRSV N was used as positive control.

**Figure S2: FACS plots showing the gating strategy for the sorting of single PRRSV-specific memory B cells**

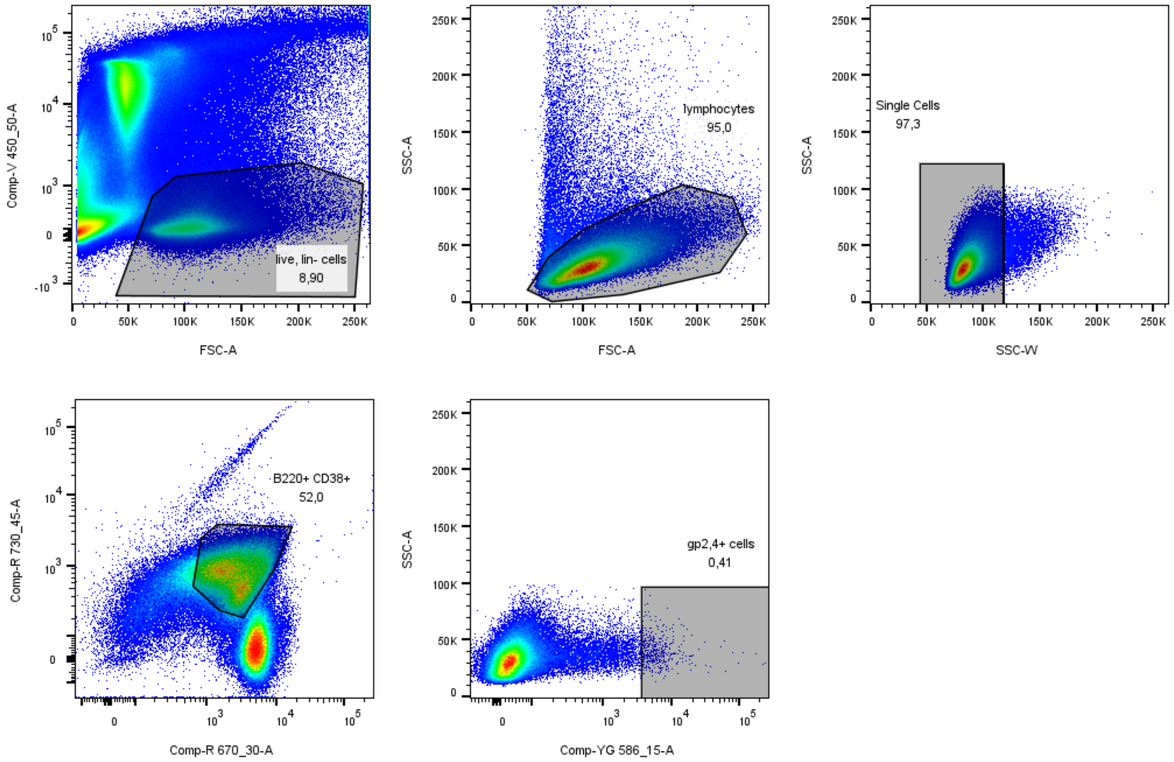


Figure S2: FACS plots showing the gating strategy for sorting single PRRSV-specific memory B cells.

**Figure S3: Validation of positive and negative control sera**

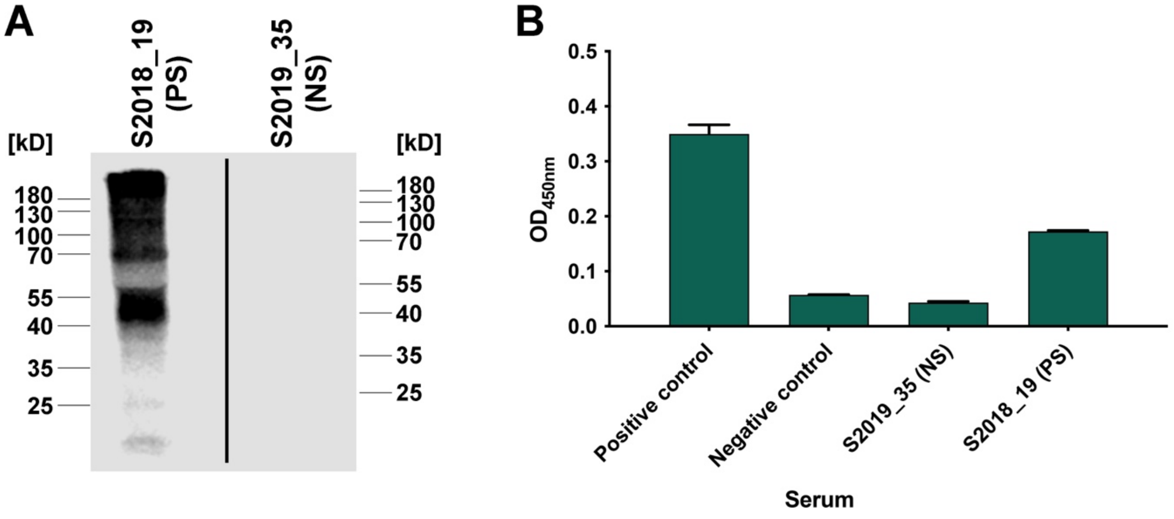


Figure S3: Characterization of the porcine control sera by (A) Western blot analysis and (B) ELISA. (A) Purified PRRSV type 2 strain VR2332 virions were analysed by SDS-PAGE under non-reducing conditions and blotted onto a nitrocellulose membrane. The negative porcine serum S2019_35 shows no specific reactivity with the virus proteins. In contrast, the neutralizing porcine serum S2018_19 produces many different bands, indicating strong reactivity against PRRSV proteins. Due to the type of analysis, a precise assignment of the reactivity to individual viral proteins remains impossible. (B) Seroreactivity was assessed using a commercial ELISA kit (IDEXX PRRS X3) according to the manufacturer's instructions. Individual sample values and kit controls are shown with error bars indicating mean ± SD. Serum S2018_19 exceeded the seropositivity cut-off (S/P ≥ 0.4), whereas serum S2019_35 remained below the threshold and was classified as negative.

**Figure S4: Specificity of IgG#18 neutralizing activity**

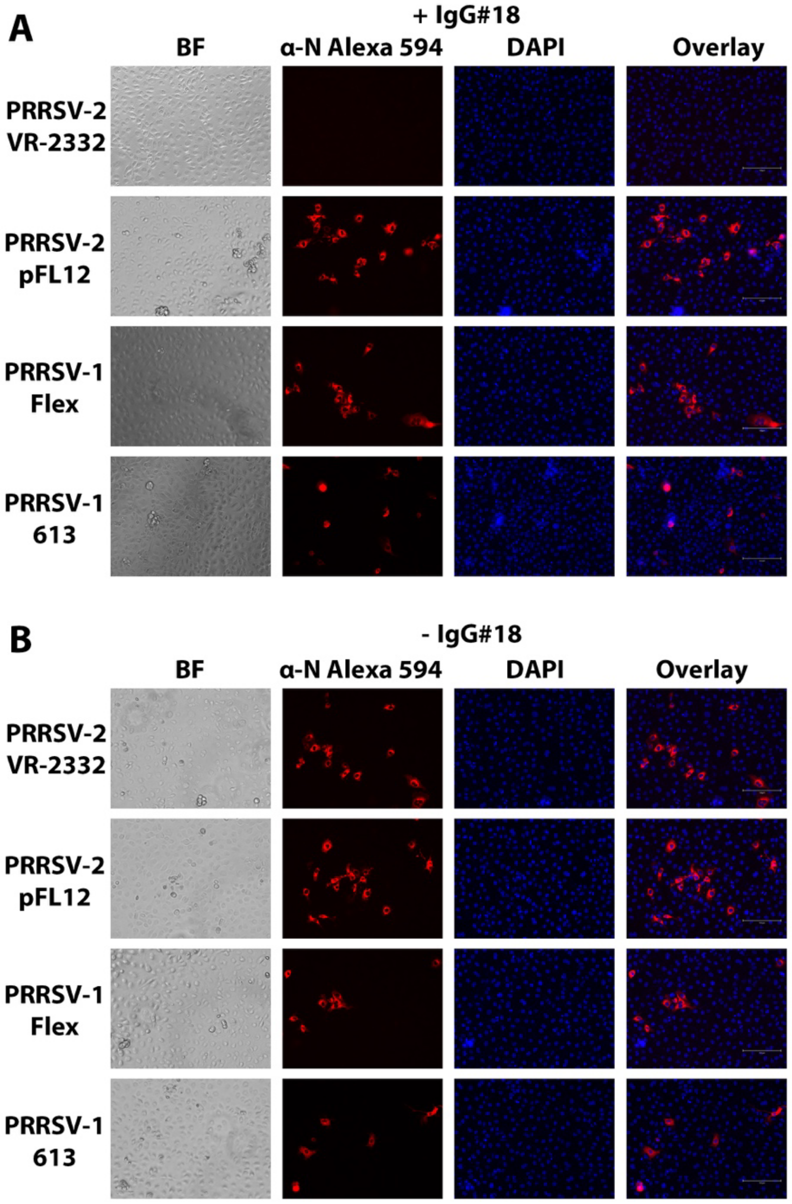


Figure S4: Breadth of the IgG#18 neutralizing activity. (A) PRRSV-2 VR-2332 was incubated in presence or absence of IgG#18 as described in Material and Methods. Indirect immunofluorescence analysis of indicator cells using the α-PRRSV-N monoclonal antibody clone 810 (Alexa 594) revealed complete virus neutralization for PRRSV-2 VR-2332, but no inhibition of entry of other PRRSV strains.

**Figure S5: Immunofluorescence analysis using IgG #18**

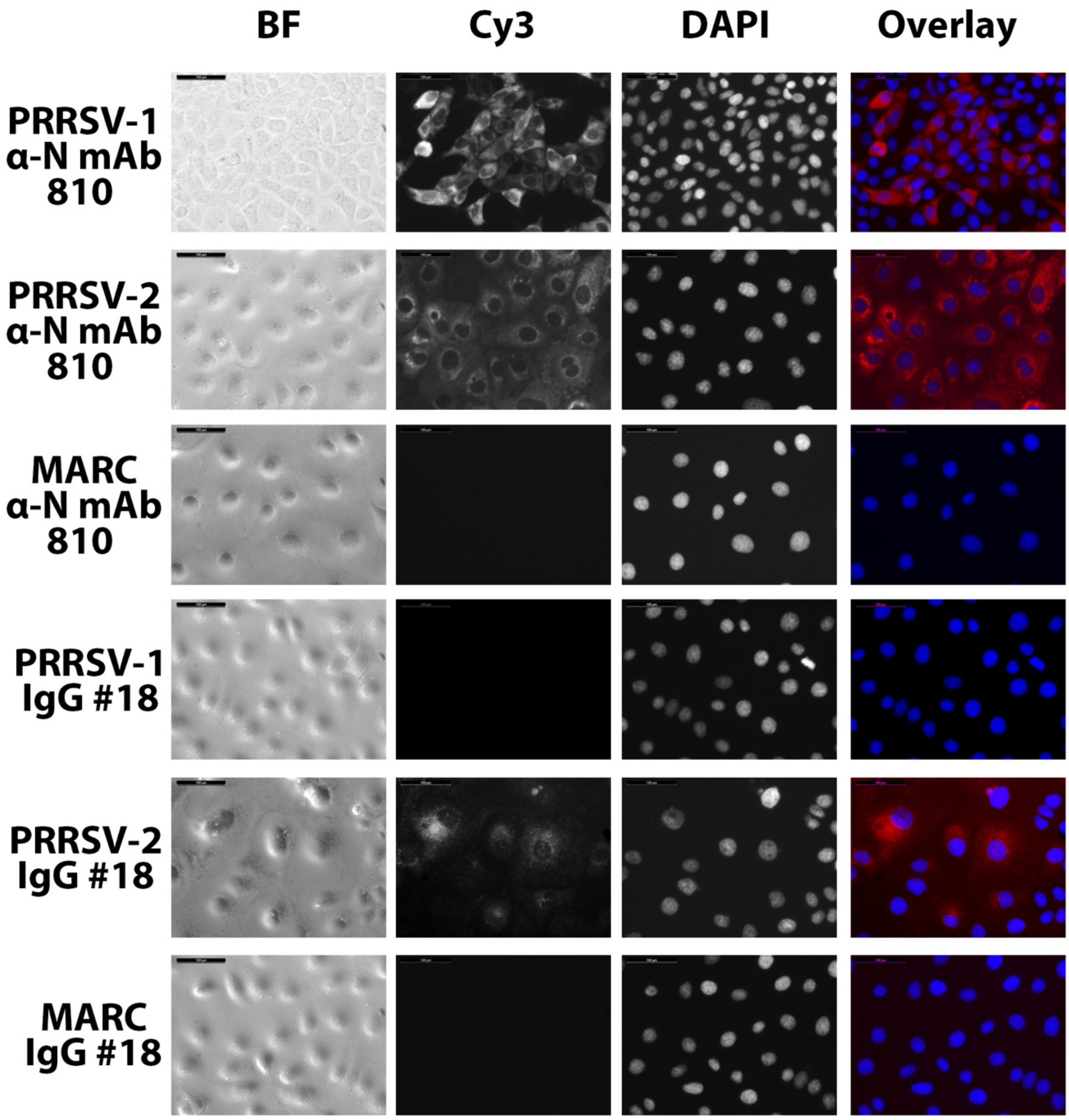


Figure S5: Indirect immunoflorescence analysis of non-infected MARC-145 cells serving as control and MARC-145 cells infected with either PRRSV-1 (strain Lelystad) or PRRSV-2 (strain VR2332) using the indicated primary antibodies (Cy3). Brightfield (BF) microscopy was used to locate individual cells and nuclei were labelled with DAPI.

**Figure S6: Western blot analysis using IgG #18**

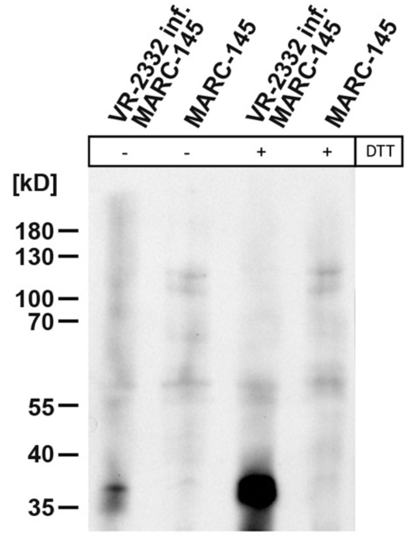


Figure S6: Western blot analysis of non-infected MARC-145 cells and MARC-145 cells infected with PRRSV-2 VR-2332 using IgG #18.

**Figure S7: Phylogenetic analysis of the IgG #18 epitope sequence**

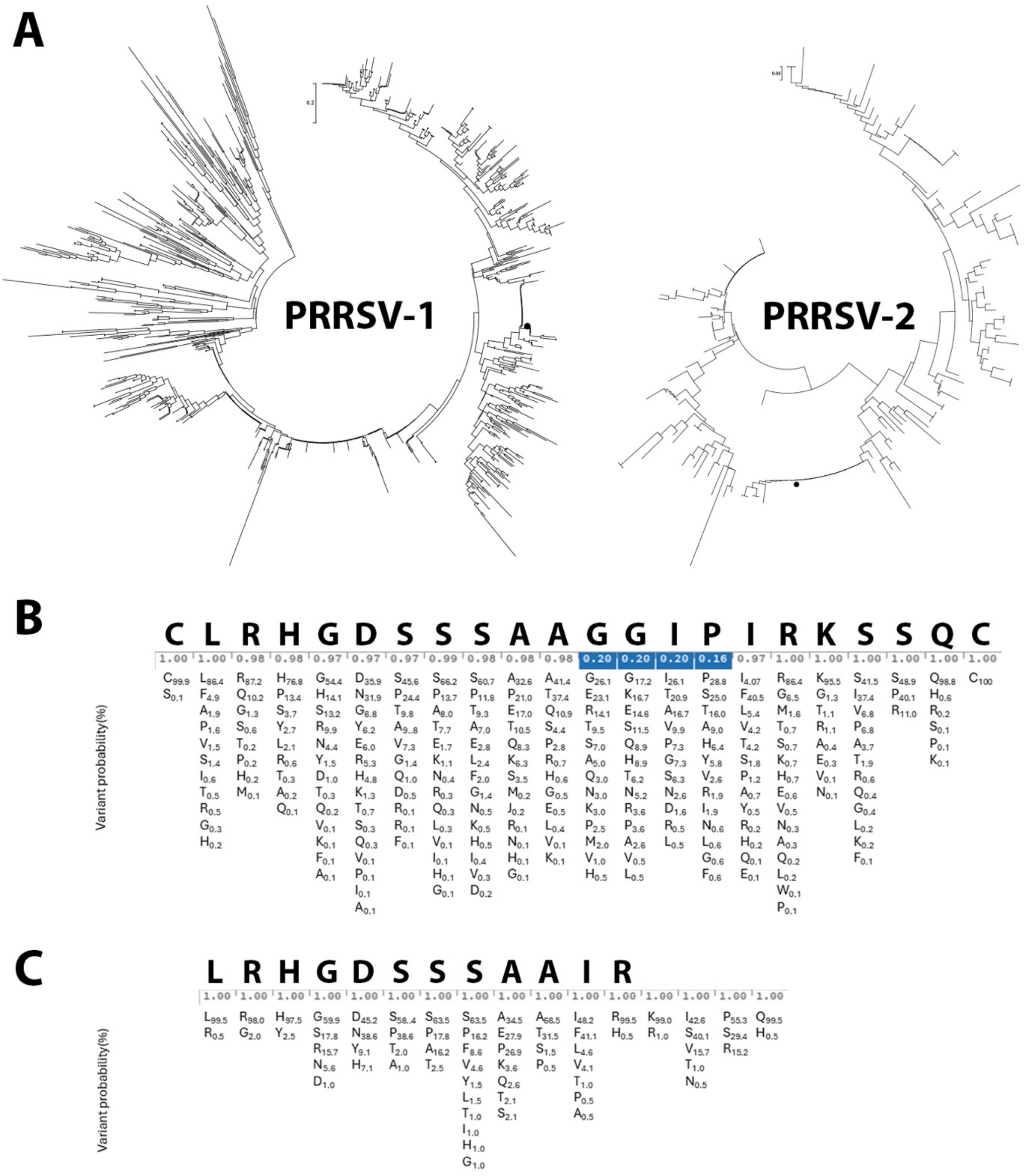


Figure S7: (A) Evolutionary history of the epitope region in PRRSV-1 (left) and PRRSV-2 (right) was inferred using the Neighbor-Joining method. The optimal trees with the sum of branch length = 67.74588606 and 9.22418043 for PRRSV-1 and PRRSV-2, respectively, are shown. Trees are drawn to scale, with branch lengths in the same units as those of the evolutionary distances used to infer the phylogenetic tree. The evolutionary distances were computed using the JTT matrix-based method and are in the units of the number of amino acid substitutions per site. The analysis of PRRSV-1 involved 972 amino acid sequences (see supplementary data). All positions with less than 95% site coverage were eliminated. That is, fewer than 5% alignment gaps,

missing data, and ambiguous bases were allowed at any position. There were a total of 18 positions in the final dataset. The analysis of PRRSV-2 involved 197 amino acid sequences (see supplementary data), which mostly represent field strains observed between 2004 and 2024, with more than 70% of them sampled from 2016 onwards. Only ~17% of these sequences represent cultured viruses, approximately 83% are derived from field isolates. All positions with less than 95% site coverage were eliminated. That is, fewer than 5% alignment gaps, missing data, and ambiguous bases were allowed at any position. (B+C) Sequence variability in the GP4 epitope of PRRSV-1 (B) and PRRSV-2 (C). Below the consensus sequence, the total amino acid frequencies and individual residue distributions are shown. An insertion in ~20% of the PRRSV-1 sequences is highlighted by blue boxes.

**Table S1: Diffraction data collection and refinement statistics**

| | scFv #18 + epitope peptide |
|---|---|
| **Data collection** | |
| Space group | P $2_1$ $2_1$ 2 |
| Resolution (Å) | 43.48 -2.30 (2.45 – 2.30) |
| Cell dimensions | |
| *a, b, c* (Å) | 72.24 108.78 50.15 |
| *α, β, γ* (°) | 90.0 90.0 90.0 |
| PDB code | XXXX |
| Complexes in AU | 1 |
| $R_{meas}$ | 0.239 (2.135) |
| < I/***σ*** (I) > | 8.22 (1.11) |
| $CC_{1/2}$ | 0.997 (0.75) |
| Completeness (%) | 100 (100) |
| Redundancy | 13.29 (13.09) |
| | |
| **Refinement** | |
| Resolution (Å) | 45.55 -2.30 (2.35 – 2.30) |
| No. reflections | 14639 (407) |
| $R_{work}$ / $R_{free}$ | 0.204 / 0.246 (0.258 / 0.242) |
| No. atoms | |
| Protein | 1926 |
| ligands | 17 |
| solvent | 175 |
| | |
| Average B-factor (Å$^2$) | 40.21 |
| R.m.s deviations | |
| Bond lengths (Å) | 0.007 |
| Bond angles (°) | 0.97 |
| Peptide Omega torsion angles (°) | 3.27 |
| Ramachandran plot[§] | |
| Favored (%) | 96.31 |
| Allowed (%) | 3.28 |
| Outliers (%) | 0.41 |
| Rotamer outliers (%) | 0.94 |
| Clashscore | 5.24 |

A single crystal was used to collect each of the diffraction data sets.
[a] Values for highest resolution shell in parentheses.
[§] Ramachandran statistics were calculated with MolProbity.

**Table S2: Kinetic parameters of scFv #18 binding to sc-GP2GP4 and the epitope peptide**

| | $k_{on}$ ($M^{-1}$ x $s^{-1}$) ± SE | $k_{off}$ ($s^{-1}$) ± SE | $K_d$ (nM) (± SE) |
|---|---|---|---|
| Epitope peptide over scFv #18 [a] | 8.42 (±1.22) x $10^5$ | 1.64 (±0.73) x $10^{-3}$ | 1.89 (±0.56) |
| sc-GP2GP4 over scFv #18 [a] | 1.99 (±0.42) x $10^4$ | 6.3 (±1.2) x $10^{-4}$ | 31.8 (±3.36) |

[a] kinetic binding parameters were determined in three independent experiments and are shown as mean values with standard errors

## Supplementary data: Epitope variability in GP4 of PRRSV-1 and PRRSV-2

## PRRSV-1

```
>AAG02137.1_glycosylated_envelope_protein_GP4_Porcine_reproductive_and_respiratory_syndrome_virus
CLRHRDSASEA----IRKIPQC
>AAX18329.1_GP4_envelope_protein_Porcine_reproductive_and_respiratory_syndrome_virus
CLRHRDSASEA----IRKIPQC
>AEI72615.1_glycosylated_minor_envelope_protein_GP4_Porcine_reproductive_and_respiratory_syndrome_virus_TJM
CLRHGDSSSPT----IRKSSQC
>ABL60909.1_GP4_Porcine_reproductive_and_respiratory_syndrome_virus_HUB2
CLRHGDSSSPT----IRKSSQC
>A0MD33.1_Glycoprotein_4_PRRSV_SD_01-08
CSRPHEASATQGQVPSRKSSQC
>ABO68992.1_envelope_glycoprotein_GP4_Porcine_reproductive_and_respiratory_syndrome_virus_HNly
CLRHGDSSSPT----IRKSSQC
>YP_009505552.1_GP4_envelope_protein_Porcine_reproductive_and_respiratory_syndrome_virus_2_ATCC_VR-2332
CLRHRDSASEA----IRKIPQC
>ABO68987.1_envelope_glycoprotein_GP4_Porcine_reproductive_and_respiratory_syndrome_virus_HNld
CLRHGDSSSPT----IRKISQC
>AAQ02973.1_GP4_glycoprotein_Porcine_reproductive_and_respiratory_syndrome_virus
CLRHGDSSSQT----IRKSSQC
>ACP43582.1_glycosylated_membrane_protein_Porcine_reproductive_and_respiratory_syndrome_virus_SX2009
CLRHGDSSSPT----IRKSSQC
>AAK44215.1_GP4_envelope_protein_Porcine_reproductive_and_respiratory_syndrome_virus_CH-1a
CLRHGDSSSPT----IRKSSQC
>ASL68486.1_GP4_envelope_protein_Porcine_reproductive_and_respiratory_syndrome_virus
CLRHRDSASEA----IRKIPQC
>WDS50280.1_glycoprotein_GP4_Porcine_reproductive_and_respiratory_syndrome_virus
CLRHRDSASEA----IRKIPQC
>AFH96245.1_envelope_glycoprotein_GP4_Porcine_reproductive_and_respiratory_syndrome_virus_437-05
CLRPHGVSAAQEEIPFGKSSQC
>QGY98672.1_glycosylated_protein_4_Porcine_reproductive_and_respiratory_syndrome_virus
CLRHRDSAPEA----IRKIPQC
>AAM18562.1_glycosylated_membrane_protein_Porcine_reproductive_and_respiratory_syndrome_virus
CLRHGDSSFPT----IRKSSQC
>AFH96238.1_envelope_glycoprotein_GP4_Porcine_reproductive_and_respiratory_syndrome_virus_H2
CFRPHGVSAAQEKIPFGKSSQC
>UNS08002.1_GP4_Porcine_reproductive_and_respiratory_syndrome_virus_PRRSV2/CN/FJGD01/2021
CLRHNYPTSAA----FRKIPQC
>QNL10135.1_GP4_Porcine_reproductive_and_respiratory_syndrome_virus_2061291
CLRHRDSASEA----IRKIPQC
>AAD27662.1_GP4_envelope_protein_Porcine_reproductive_and_respiratory_syndrome_virus_PrimePac_PRRS_vaccine
CLRHGDPSSAA----IRKSSQC
>ACZ67533.1_glycosylated_membrane_protein_GP4_Porcine_reproductive_and_respiratory_syndrome_virus_GD
CLRHGDSSSPT----IRKISQC
>ARH13095.1_GP4_Porcine_reproductive_and_respiratory_syndrome_virus
CLRHSNPTPAA----FRKIPQC
>WAA68443.1_GP4_protein_Porcine_reproductive_and_respiratory_syndrome_virus_GD-H1
CLRHNYSSSAA----FRKIPQC
>ACZ54922.1_GP4_Porcine_reproductive_and_respiratory_syndrome_virus_08HuN
CLRHGDSSSPT----IRKSSQC
>ABE73141.1_GP4_envelope_protein_Porcine_reproductive_and_respiratory_syndrome_virus
CLRHRDSASEA----IRKIPQC
>XGU09610.1_GP4_Porcine_reproductive_and_respiratory_syndrome_virus
CLRHGDSPSPT----IRKSSQC
>ACV91353.1_GP4_Porcine_reproductive_and_respiratory_syndrome_virus_JXM20
CLRHGDSSSPT----IRKSSQC
>AFP43977.1_glycosylated_protein_4_Porcine_reproductive_and_respiratory_syndrome_virus_NADC30
CLRHSNPSPAA----FRKIPQC
>ABJ51872.1_glycoprotein_4_Porcine_reproductive_and_respiratory_syndrome_virus
CLRHGDSSSQT----IRKSSQC
>AAG49623.1_envelope_protein_GP4_Porcine_reproductive_and_respiratory_syndrome_virus
CLRHRDSASEA----IRKIPQC
>QCC26668.1_GP4_envelope_protein_Porcine_reproductive_and_respiratory_syndrome_virus_SWU/YB2/2018
CLRHSNPSSAA----IRKVRQC
>ACI01447.1_GP4_Porcine_reproductive_and_respiratory_syndrome_virus_94V360
CPRPHGASTAQA----RKPSQC
>ACN93840.1_GP4_Porcine_reproductive_and_respiratory_syndrome_virus_JXA1_P45
CLRHGDSSSPT----IRKSSQC
>ABB18261.1_GP4_Porcine_reproductive_and_respiratory_syndrome_virus
CLRHGHSSSEA----TRKSRQC
>AFK09151.1_GP4_Porcine_reproductive_and_respiratory_syndrome_virus_10-10GX-3
CLRHGDSSFPT----IRKSSQC
>AFS30917.1_GP4_Porcine_reproductive_and_respiratory_syndrome_virus_JX
CLRHGDSSSPT----IRKSSQC
>AAR19404.1_GP4_PRRSV_HN1
CLRHRGSAPEA----IRKIPQC
>AFH96247.1_envelope_glycoprotein_GP4_Porcine_reproductive_and_respiratory_syndrome_virus_472-05
CLSPT-----TQKGNFRKPSQC
>AAQ02919.1_GP4_glycoprotein_Porcine_reproductive_and_respiratory_syndrome_virus
CLRHRNSASEA----IRKVPQC
>XTP63242.1_GP4_Porcine_reproductive_and_respiratory_syndrome_virus_HLJZD25-1810
CGQSYGSR--------RKPSQC
>XYA24623.1_GP4_Porcine_reproductive_and_respiratory_syndrome_virus_SDVD-JNJX
CLRHGNPTSAT----FRKIPQC
>XTP63252.1_GP4_Porcine_reproductive_and_respiratory_syndrome_virus_HNLCL7-1804
```

CAQPREVSTTSPKT-FGRSSQC
>XTP63302.1_GP4_Porcine_reproductive_and_respiratory_syndrome_virus_SDHSW160-2201
CPRPRGAAAART----QGPSQC
>XTP63262.1_GP4_Porcine_reproductive_and_respiratory_syndrome_virus_HNLCL75-1812
CPRSHGTSTTPGTA-LGKSPQC
>AAP57405.1_GP4_PRRSV_HB-2(sh)/2002
CLRYGDSPPPT----VRKSSQC
>XTP63235.1_GP4_Porcine_reproductive_and_respiratory_syndrome_virus_HLJWK335-2005
CSRTRGVSTASSEV-FRKSSQC
>XTP63292.1_GP4_Porcine_reproductive_and_respiratory_syndrome_virus_GDXNF85-1803
CAQSHGAS--------SKPSQC
>XTP63308.1_GP4_Porcine_reproductive_and_respiratory_syndrome_virus_WK925
CTHLREAPTPPRGI-FRTPSQC
>XTP63272.1_GP4_Porcine_reproductive_and_respiratory_syndrome_virus_PY61
CHRPYEASTAQGSSSFPNPSQC
>XTP63172.1_GP4_Porcine_reproductive_and_respiratory_syndrome_virus_TZJ2780
CRRTHETSTAGGGDSFAKPSQC
>XTP63282.1_GP4_Porcine_reproductive_and_respiratory_syndrome_virus_GDXNF41-1801
CGQSYGPR--------HKSSQC
>XTP63212.1_GP4_Porcine_reproductive_and_respiratory_syndrome_virus_TJWK169-1804
CVQPHEASTTPPRT-FGTSSQC
>XTP63162.1_GP4_Porcine_reproductive_and_respiratory_syndrome_virus_LNDB50-1806
CIR-----LREASATRRTSPQC
>XTP63202.1_GP4_Porcine_reproductive_and_respiratory_syndrome_virus_GDXNF73-1802
CVQSHEAS--------FKPSQC
>XTP63222.1_GP4_Porcine_reproductive_and_respiratory_syndrome_virus_XJTZJ158-2001
CAQPREVSTASPKT-FERSSQC
>XTP63192.1_GP4_Porcine_reproductive_and_respiratory_syndrome_virus_ZZH817
CLQSRRASATQGQTPIRKASQC
>XTP63182.1_GP4_Porcine_reproductive_and_respiratory_syndrome_virus_TZJ2781
CPR-----PHEGSAGPRGPPQC
>XTP63108.1_GP4_Porcine_reproductive_and_respiratory_syndrome_virus_HLJWK14-1611
CPRAHEVPTASSES-LRKSSQC
>XTP63128.1_GP4_Porcine_reproductive_and_respiratory_syndrome_virus_IMWK141-1801
CARPRTASTTHSTI-LGTSSQC
>WYW90990.1_GP4_Porcine_reproductive_and_respiratory_syndrome_virus
CLRHSYPSSAA----FRKIPQC
>XTP63118.1_GP4_Porcine_reproductive_and_respiratory_syndrome_virus_HNLCL53-1812
CAQSREASTAPSGT-FGTPSQC
>XTP63098.1_GP4_Porcine_reproductive_and_respiratory_syndrome_virus_HLJWG9-1612
CAQSREASTTPHGT-FGTPSQC
>XXQ18084.1_GP4_Porcine_reproductive_and_respiratory_syndrome_virus
CLRHGNPPSAT----IRKIPQC
>XYA23761.1_GP4_Porcine_reproductive_and_respiratory_syndrome_virus_PRRSV01-2024
CLRHGNSTSAT----FRKIPQC
>QDH09207.1_glycoprotein_4_Porcine_reproductive_and_respiratory_syndrome_virus
CLRPHGVSAAQEKIPFGKSSQC
>QXF30726.1_GP4_Porcine_reproductive_and_respiratory_syndrome_virus
CLRPHGVSAAQEKIPFGKSSQC
>XSG70310.1_GP4_Porcine_reproductive_and_respiratory_syndrome_virus
CLRHGDPSGKT----FRKIPQC
>ACV89498.1_GP4_Porcine_reproductive_and_respiratory_syndrome_virus_SHE
CLRPHGVSTAQENIPFGKPSQC
>XUM38540.1_GP4_Porcine_reproductive_and_respiratory_syndrome_virus
CLRHGNPPSAT----IRKIPQC
>XSG70329.1_GP4_Porcine_reproductive_and_respiratory_syndrome_virus
CLRHGNTTSAA----FSKIRQC
>XTO96116.1_GP4_Porcine_reproductive_and_respiratory_syndrome_virus_PRRSGARD_vaccine
CLRHGHSSSEA----TRKSRQC
>XSG70383.1_GP4_Porcine_reproductive_and_respiratory_syndrome_virus
CLRHGNTTSET----FRKIRQC
>XSG70398.1_GP4_Porcine_reproductive_and_respiratory_syndrome_virus
CLRHGNTASEA----FRKIRQC
>XSG70373.1_GP4_Porcine_reproductive_and_respiratory_syndrome_virus
CLRHGNTTSEA----FRKIRQC
>XSG70324.1_GP4_Porcine_reproductive_and_respiratory_syndrome_virus
CLRHGNTSSEA----FRKIRSC
>XSG70388.1_GP4_Porcine_reproductive_and_respiratory_syndrome_virus
CLRHGNTTNKA----FRKFRQC
>XSG70339.1_GP4_Porcine_reproductive_and_respiratory_syndrome_virus
CLRHGDPSGKT----FRKIPHC
>XSG70358.1_GP4_Porcine_reproductive_and_respiratory_syndrome_virus
CFGHGNTSSET----SRKIRQC
>XSG70334.1_GP4_Porcine_reproductive_and_respiratory_syndrome_virus
CLRHGNTTSAA----FSKIRQC
>ANP23745.1_GP4_Porcine_reproductive_and_respiratory_syndrome_virus_14-64
CLRHRDSASKA----IRKIPQC
>XSG70368.1_GP4_Porcine_reproductive_and_respiratory_syndrome_virus
CLRHGNTTSKA----FSKVRQC
>XSG70363.1_GP4_Porcine_reproductive_and_respiratory_syndrome_virus
CLRHGDPSGKT----FRKIPQC
>XSG70343.1_GP4_Porcine_reproductive_and_respiratory_syndrome_virus
CFRHGNPSSAT----FRKIPQC
>XSG70348.1_GP4_Porcine_reproductive_and_respiratory_syndrome_virus
CLRHGNTTTET----FRKIRQC
>XSG70378.1_GP4_Porcine_reproductive_and_respiratory_syndrome_virus
CSGHGNTTSQT----FRKIRQC
>XSG70393.1_GP4_Porcine_reproductive_and_respiratory_syndrome_virus
CLRHDNPSSAT----FRKIPQC
>XSG70353.1_GP4_Porcine_reproductive_and_respiratory_syndrome_virus

CLRHGNTSPKT----FRKIRQC
>XOD12153.1_glycoprotein_4_Betaarterivirus_americense
CLRYGNPSSAG----LRKIPRC
>XRS45987.1_GP4_Porcine_reproductive_and_respiratory_syndrome_virus_GDJS2024
CLRHNYPTSAA----FRKIPQC
>XRL74629.1_GP4_Porcine_reproductive_and_respiratory_syndrome_virus
CPRPHEGPIGS-----RGPSQC
>XQZ12458.1_GP4_Porcine_reproductive_and_respiratory_syndrome_virus
CLRHSYPSSAT----FRKVPQC
>XQZ12474.1_GP4_Porcine_reproductive_and_respiratory_syndrome_virus
CLRHSNPSPAT----IRKVPQC
>XQZ12466.1_GP4_Porcine_reproductive_and_respiratory_syndrome_virus
CLRHGNSSPKT----FRKIPQC
>AFJ92663.1_GP4_Porcine_reproductive_and_respiratory_syndrome_virus_NVDC-JS2-2011
CLRHGDSSSPT----IRKSSQC
>XQK38486.1_GP4_Porcine_reproductive_and_respiratory_syndrome_virus
CLRHGHTSPAA----FRKIRQC
>XQK38494.1_GP4_Porcine_reproductive_and_respiratory_syndrome_virus
CLRHNYPTSAA----FRKIPQC
>AHG56687.1_ORF4_Porcine_reproductive_and_respiratory_syndrome_virus
CLRPHGVSAAQEEIPFGKSSQC
>XKR82588.1_GP4_Porcine_reproductive_and_respiratory_syndrome_virus_PRRSV1/CN/FJEU02/2023
CTRLHGGL--------RKPSQC
>XOL43270.1_GP4_Porcine_reproductive_and_respiratory_syndrome_virus
CLRHGDSSSPT----IRKSSQC
>XIE10273.1_GP4_Porcine_reproductive_and_respiratory_syndrome_virus
CLRHNNPPSAT----FRKIPQC
>XII92264.1_GP4_Porcine_reproductive_and_respiratory_syndrome_virus_HLJTZJ155-2001
CGQSYGSH--------RKPSQC
>XMF59031.1_GP4_Porcine_reproductive_and_respiratory_syndrome_virus
CLRHSYPSSAA----FRKVPQC
>XIE10263.1_GP4_Porcine_reproductive_and_respiratory_syndrome_virus
CLRHDYSTSAT----FRKIPQC
>XII92255.1_GP4_Porcine_reproductive_and_respiratory_syndrome_virus_GDXNF161-1806
CTRPHETI--------GKPSQC
>XHY98762.1_GP4_Porcine_reproductive_and_respiratory_syndrome_virus
CLRHGDSSSPT----IRKSSQC
>XII92244.1_GP4_Porcine_reproductive_and_respiratory_syndrome_virus_GDXNF94-1804
CTQLYRTP--------RKPSQC
>XHB18298.1_GP4_Porcine_reproductive_and_respiratory_syndrome_virus
CLRHNYSPFAA----SRKIPQC
>XDB37635.1_ORF4_porcine_reproductive_and_respiratory_syndrome_virus_1_GD2023
CSRPSGVSTTQS----QRPSQC
>XFF06255.1_GP4_Porcine_reproductive_and_respiratory_syndrome_virus_L1C.5
CLRHNYPSSAA----FRKTPQC
>WLQ22742.1_GP4_Porcine_reproductive_and_respiratory_syndrome_virus_136-2022
CLPSRSASTTPTNLSFGKTSQC
>XGZ25579.1_GP4_Porcine_reproductive_and_respiratory_syndrome_virus
CLRHSNPSSAA----FRKVPQC
>XBO77379.1_GP4_Porcine_reproductive_and_respiratory_syndrome_virus_HLJDZD55
CLRHGNSSTAA----FRKIPQC
>XDD73014.1_GP4_Porcine_reproductive_and_respiratory_syndrome_virus
CLRHSSPSPAA----FRKIPQC
>WDV74448.1_GP4_Porcine_reproductive_and_respiratory_syndrome_virus
CLRHGDSSSPT----IRKSSQC
>WXB55247.1_GP4_Porcine_reproductive_and_respiratory_syndrome_virus
CLRHGNTPPAV----FRKIPQC
>XCX12193.1_GP4_Porcine_reproductive_and_respiratory_syndrome_virus_LY24
CLRHSNPPSAA----FRKIPQC
>WVH71839.1_GP4_Porcine_reproductive_and_respiratory_syndrome_virus
CLRHRDSASEA----IRKIPQC
>WVH45080.1_GP4_Porcine_reproductive_and_respiratory_syndrome_virus
CLRHNYPSSAA----FRKIPQC
>WPS90252.1_GP4_Porcine_reproductive_and_respiratory_syndrome_virus
CLRHNYPPSAA----FRKIPQC
>WUV41042.1_GP4_Porcine_reproductive_and_respiratory_syndrome_virus_NC18-9-7
CLRHNNPSSAA----FRKIPQC
>WVH71851.1_GP4_Porcine_reproductive_and_respiratory_syndrome_virus
CLRHGDSSSPT----IRKSSQC
>WVH45089.1_GP4_Porcine_reproductive_and_respiratory_syndrome_virus
CLRHNYPPSAA----FRKIPQC
>WPS90271.1_GP4_Porcine_reproductive_and_respiratory_syndrome_virus
CLRYNYSSSAA----FRKIPQC
>WPL77704.1_GP4_Porcine_reproductive_and_respiratory_syndrome_virus_sg_2107
CLRHGNPSLAA----FRKVPQC
>WPS90244.1_GP4_Porcine_reproductive_and_respiratory_syndrome_virus
CLRHNYSSSAA----FRKIPQC
>WPS90262.1_GP4_Porcine_reproductive_and_respiratory_syndrome_virus
CLRHNHSSSAA----FRKISQC
>WPL77689.1_GP4_Porcine_reproductive_and_respiratory_syndrome_virus_qy_2104
CLRHGNPPSAA----FRKIPQC
>WXB55255.1_GP4_Porcine_reproductive_and_respiratory_syndrome_virus
CLRHSNPSPAT----FRKIPQC
>ACF93751.1_GP4_Porcine_reproductive_and_respiratory_syndrome_virus
CLRHGDSSSPT----IRKSSQC
>WPL77673.1_GP4_Porcine_reproductive_and_respiratory_syndrome_virus_qy_2008
CLRHGDSSSPT----IRKSSQC
>WAX25703.1_GP4_Porcine_reproductive_and_respiratory_syndrome_virus_GDhy-1809
CLRHGNPPSAA----FRKIPQC
>ABL60917.1_GP4_Porcine_reproductive_and_respiratory_syndrome_virus_HEB1

CLRHGDSSSPT----IRKSSQC
>WPF72577.1_GP4_Porcine_reproductive_and_respiratory_syndrome_virus
CLGHNNPSSAA----FRKIPQC
>WOP33769.1_GP4_Porcine_reproductive_and_respiratory_syndrome_virus_BDSP-1
CLRHGNSTSAA----FRKVPQC
>WMZ00164.1_GP4_Porcine_reproductive_and_respiratory_syndrome_virus
CARSHGAS--------RKPSQC
>WPF72071.1_GP4_Porcine_reproductive_and_respiratory_syndrome_virus
CLRHGNPSPAA----FRKIPQC
>WNY15488.1_GP4_Porcine_reproductive_and_respiratory_syndrome_virus_SXht2012
CLRHSNPSPAA----FRKIPQC
>WNN28500.1_GP4_Porcine_reproductive_and_respiratory_syndrome_virus
CLRHSYSSSAA----FRKIPQC
>ABL60901.1_GP4_Porcine_reproductive_and_respiratory_syndrome_virus_JXA1
CLRHGDSSSPT----IRKISQC
>WNI04636.1_GP4_Porcine_reproductive_and_respiratory_syndrome_virus_SCABTC-202306
CLRHRDSASEA----IRKIPQC
>WNI04656.1_GP4_Porcine_reproductive_and_respiratory_syndrome_virus_SCABTC-202308
CLRHGNPSSAA----FRKVPQC
>WML15809.1_GP4_Porcine_reproductive_and_respiratory_syndrome_virus_SF7
CLRHSNPSPTA----FRKIPQC
>WNI04626.1_GP4_Porcine_reproductive_and_respiratory_syndrome_virus_SCABTC-202305
CLRHGNSSSAA----FRKVPQC
>WMM65941.1_GP4_Porcine_reproductive_and_respiratory_syndrome_virus
CLRHSNPSSAA----FRKVPQC
>WML15800.1_GP4_Porcine_reproductive_and_respiratory_syndrome_virus_SF5
CLRHSNPSPTA----FRKIPQC
>AGC08745.1_GP4_Porcine_reproductive_and_respiratory_syndrome_virus_JXA1-P100
CLRHGDSSSPT----IRKSSQC
>WML15818.1_GP4_Porcine_reproductive_and_respiratory_syndrome_virus_ZH12
CLRHGNPSPEA----FRKIPQC
>WMI30714.1_GP4_Porcine_reproductive_and_respiratory_syndrome_virus
CLRHSNPPPTA----FRKIPQC
>WLK78553.1_GP4_Porcine_reproductive_and_respiratory_syndrome_virus
CLRHSYSPSAA----IRKIPQC
>WLK78543.1_GP4_Porcine_reproductive_and_respiratory_syndrome_virus
CLRHGDSSSPT----IRKISQC
>WLK25999.1_GP4_Porcine_reproductive_and_respiratory_syndrome_virus_HM1805
CLRHSNPSLAA----FRKIPQC
>WLK78523.1_GP4_Porcine_reproductive_and_respiratory_syndrome_virus
CLRHRDSASEA----IRKIPQC
>QGY98072.1_glycosylated_protein_4_Porcine_reproductive_and_respiratory_syndrome_virus
CLRHGDSSFPT----IRKSSQC
>WLK26019.1_GP4_Porcine_reproductive_and_respiratory_syndrome_virus_HM1809
CLRHGNPSSAT----FRKIPQC
>WLD30081.1_GP4_Porcine_reproductive_and_respiratory_syndrome_virus
CLRHNHPTSAA----FRKIPQC
>WKW84039.1_GP4_Porcine_reproductive_and_respiratory_syndrome_virus
CLRHNHPTSAA----FRKIPQC
>WLD30091.1_GP4_Porcine_reproductive_and_respiratory_syndrome_virus
CFRHNYPASAA----FRKIPQC
>WJE88833.1_GP4_Porcine_reproductive_and_respiratory_syndrome_virus_PRRSV2/CN/G8/2018
CLRHRDSASEA----IRKIPQC
>WHT50432.1_GP4_Porcine_reproductive_and_respiratory_syndrome_virus
CLQLRQSPDARAPV-HGKPSQC
>WGO81935.1_GP4_Porcine_reproductive_and_respiratory_syndrome_virus
CLRHGDSTSTA----FRKIPQC
>AHL83466.1_GP4_Porcine_reproductive_and_respiratory_syndrome_virus_HeNan-A1
CLRHGDSSSPT----IRKIPQC
>UVH34533.1_GP4_Porcine_reproductive_and_respiratory_syndrome_virus
CLRHSNQSPAA----FRKIPQC
>WGO81915.1_GP4_Porcine_reproductive_and_respiratory_syndrome_virus
CLRHGNSTSTA----FRKIPQC
>WHT50422.1_GP4_Porcine_reproductive_and_respiratory_syndrome_virus
CLQLRQSPDARAPV-HGKPSQC
>WGO81925.1_GP4_Porcine_reproductive_and_respiratory_syndrome_virus
CLRHGNSAFAA----LRKIPQC
>UVH34523.1_GP4_Porcine_reproductive_and_respiratory_syndrome_virus
CLRHSNPLPAA----FRKVRQC
>UVH34543.1_GP4_Porcine_reproductive_and_respiratory_syndrome_virus
CLRHGDSSSPT----IRKSSQC
>UVH34552.1_GP4_Porcine_reproductive_and_respiratory_syndrome_virus
CLRHGDSSSPT----IRKSSQC
>UVH34474.1_GP4_Porcine_reproductive_and_respiratory_syndrome_virus
CLRHSNPSSAA----FRKIPQC
>UVH34444.1_GP4_Porcine_reproductive_and_respiratory_syndrome_virus
CLRHGNPSSAA----FRKVPQC
>UVH34514.1_GP4_Porcine_reproductive_and_respiratory_syndrome_virus
CLRHSNPSPAA----FRKIPQC
>UVH34454.1_GP4_Porcine_reproductive_and_respiratory_syndrome_virus
CLRHGNPPPAA----FRKIPQC
>UVH34394.1_GP4_Porcine_reproductive_and_respiratory_syndrome_virus
CLRHGDSSSPT----IRKSSQC
>UVH34494.1_GP4_Porcine_reproductive_and_respiratory_syndrome_virus
CLRHSNPSSAT----FRKIPQC
>UVH34434.1_GP4_Porcine_reproductive_and_respiratory_syndrome_virus
CLRHGDSSSPT----IRKIPHC
>UVH34424.1_GP4_Porcine_reproductive_and_respiratory_syndrome_virus
CLRHGDSSSPT----IRKSSQC
>UVH34484.1_GP4_Porcine_reproductive_and_respiratory_syndrome_virus

CLRHGNPPSSA----FRKIPQC
>UVH34404.1_GP4_Porcine_reproductive_and_respiratory_syndrome_virus
CLRHGNTASTA----FRKIRQC
>UVH34464.1_GP4_Porcine_reproductive_and_respiratory_syndrome_virus
CLRHSNPSSPA----LRKVSQC
>UVH34354.1_GP4_Porcine_reproductive_and_respiratory_syndrome_virus
CLRHSNPPLPA----FRKIPQC
>UVH34414.1_GP4_Porcine_reproductive_and_respiratory_syndrome_virus
CLRHRDSASEA----IRKIPQC
>UVH34344.1_GP4_Porcine_reproductive_and_respiratory_syndrome_virus
CLRHSYPSSAA----FRKVPQC
>UVH34304.1_GP4_Porcine_reproductive_and_respiratory_syndrome_virus
CLGHGNSSSKT----FRKIPQC
>UVH34384.1_GP4_Porcine_reproductive_and_respiratory_syndrome_virus
CLRHGNPSSPT----IRKSSQC
>UVH34294.1_GP4_Porcine_reproductive_and_respiratory_syndrome_virus
CLRHSNPSLAA----FRKIPQC
>UVH34374.1_GP4_Porcine_reproductive_and_respiratory_syndrome_virus
CLRHGDSSSKT----FRKIPQC
>UVH34324.1_GP4_Porcine_reproductive_and_respiratory_syndrome_virus
CLRHSNPSSTA----FRKVPQC
>UVH34334.1_GP4_Porcine_reproductive_and_respiratory_syndrome_virus
CLRHSNPSSAA----FRKVPQC
>UVH34314.1_GP4_Porcine_reproductive_and_respiratory_syndrome_virus
CLRHGDSSSPT----IRKSSQC
>WEU54112.1_GP4_Porcine_reproductive_and_respiratory_syndrome_virus_HBag-4
CLRHSNPSSAA----FRKVPQC
>UVH34284.1_GP4_Porcine_reproductive_and_respiratory_syndrome_virus
CLRHGDSSSPT----IRKSSQC
>UVH34274.1_GP4_Porcine_reproductive_and_respiratory_syndrome_virus
CLRHRDSASEA----IRKIPQC
>WDV74382.1_GP4_Porcine_reproductive_and_respiratory_syndrome_virus
CLRHGNPSPAA----FRKIPQC
>WDV74407.1_GP4_Porcine_reproductive_and_respiratory_syndrome_virus
CLRHGDSSSPT----IRKSSQC
>WDV74440.1_GP4_Porcine_reproductive_and_respiratory_syndrome_virus
CLRHGNSSPTA----FRKIPQC
>WDV74399.1_GP4_Porcine_reproductive_and_respiratory_syndrome_virus
CLRHGNPSPAA----FRKIPQC
>WDV74374.1_GP4_Porcine_reproductive_and_respiratory_syndrome_virus
CLRHGNPSPAA----FRKIPQC
>WCD58735.1_GP4_Porcine_reproductive_and_respiratory_syndrome_virus_GER18-258
CSQPREGPAASSNVPYAKQSQC
>WBG54426.1_GP4_Porcine_reproductive_and_respiratory_syndrome_virus
CLQAP-----------GTRSQC
>WAX25712.1_GP4_Porcine_reproductive_and_respiratory_syndrome_virus_GDqy-1909
CLRHGDPSSPT----IRKSSQC
>QEE94760.1_GP4_Porcine_reproductive_and_respiratory_syndrome_virus_PRRSV/pig/CHN/JK/201805
CLRHSDSSSST----IRKSSQC
>ANP23962.1_GP4_Porcine_reproductive_and_respiratory_syndrome_virus_ISU29
CLRHNNPSSAA----FRKIPQC
>QWE49767.1_GP4_synthetic_construct
CLRHSNPSSAA----FRKIPQC
>UXO31261.1_GP4_Porcine_reproductive_and_respiratory_syndrome_virus
CLRHGDSSSPT----IRKISQC
>WCD58743.1_GP4_Porcine_reproductive_and_respiratory_syndrome_virus
CLPSRNASTAQ----IRKISQC
>UUS63670.1_GP4_Porcine_reproductive_and_respiratory_syndrome_virus_2_NADC30-like
CLRHSNPSSAA----FRKISQC
>UHY43695.1_glycosylated_membrane_protein_4_Porcine_reproductive_and_respiratory_syndrome_virus
CLRHNYPTSAA----FRKIPQC
>UXX23812.1_GP4_Porcine_reproductive_and_respiratory_syndrome_virus
CLRHGNTTSKA----LRKIPHC
>QXF30756.1_GP4_Porcine_reproductive_and_respiratory_syndrome_virus
CLRPHGVSAAQKEIPFGKSSQC
>USK12060.1_GP4_Porcine_reproductive_and_respiratory_syndrome_virus
CLRPHGVSAAQAKISFGKSSQC
>UJD73078.1_GP4_Porcine_reproductive_and_respiratory_syndrome_virus_2
CLRHSNPSPAA----FRKVPQC
>QXF30734.1_GP4_Porcine_reproductive_and_respiratory_syndrome_virus
CLRPHGVSAAQEKIPFGKSSQC
>QXF30762.1_GP4_Porcine_reproductive_and_respiratory_syndrome_virus
CLRPHGVSAAQEKIPFGKSSQC
>UQE92913.1_GP4_Porcine_reproductive_and_respiratory_syndrome_virus
CLRHGDSSSPT----IRKISQC
>ANP23892.1_GP4_Porcine_reproductive_and_respiratory_syndrome_virus_ISU10
CLRHNNPSSAA----FRKIPQC
>UOF03347.1_GP4_Porcine_reproductive_and_respiratory_syndrome_virus_YL-2021
CLRHGYSSSPA----IRKSSQC
>UNS08186.1_GP4_Porcine_reproductive_and_respiratory_syndrome_virus_PRRSV2/CN/F3/2020
CLRHGDSSSPT----IRKSSQC
>URX64825.1_GP4_Porcine_reproductive_and_respiratory_syndrome_virus
CLRHSNPSPAA----FRKVPQC
>UNS08210.1_GP4_Porcine_reproductive_and_respiratory_syndrome_virus_PRRSV2/CN/J2/2019
CLRHGNPSPAA----FRKIPQC
>UNS08178.1_GP4_Porcine_reproductive_and_respiratory_syndrome_virus_PRRSV2/CN/F2/2019
CLRHGNSSSET----FRKIPQC
>QXF30742.1_GP4_Porcine_reproductive_and_respiratory_syndrome_virus
CLRPHGVSAAQEKIPFGKSSQC
>UNS08170.1_GP4_Porcine_reproductive_and_respiratory_syndrome_virus_PRRSV2/CN/L2/2019

CLRHGDSSSPT----IRKSSQC
>UNS08074.1_GP4_Porcine_reproductive_and_respiratory_syndrome_virus_PRRSV2/CN/N4/2019
CLRHSDTSSKT----FRKIPQC
>UNS08154.1_GP4_Porcine_reproductive_and_respiratory_syndrome_virus_PRRSV2/CN/C2/2019
CLRHGDSSSPT----IRKSSQC
>UNS08114.1_GP4_Porcine_reproductive_and_respiratory_syndrome_virus_PRRSV2/CN/N42/2017
CLRHSDTSSKT----FRKIPQC
>UNS08066.1_GP4_Porcine_reproductive_and_respiratory_syndrome_virus_PRRSV2/CN/L4/2020
CLRHGNTTSKA----LRKIPHC
>UNS08130.1_GP4_Porcine_reproductive_and_respiratory_syndrome_virus_PRRSV2/CN/F7/2017
CLRHGNTPSAA----FRKIRQC
>UNS08034.1_GP4_Porcine_reproductive_and_respiratory_syndrome_virus_PRRSV2/CN/G9/2018
CLRHGDSSSTT----FRKIPQC
>UNS08026.1_GP4_Porcine_reproductive_and_respiratory_syndrome_virus_PRRSV2/CN/G7/2018
CLRHSNPSFAA----FRKIPQC
>UNS08042.1_GP4_Porcine_reproductive_and_respiratory_syndrome_virus_PRRSV2/CN/H1/2018
CLRHGHSSSET----FRKISQC
>UNS08018.1_GP4_Porcine_reproductive_and_respiratory_syndrome_virus_PRRSV2/CN/F5/2018
CLRYDNPSSAA----FRKIPQC
>QWM97671.1_GP4_Porcine_reproductive_and_respiratory_syndrome_virus_PRRSV2/CN/N9185/2018
CLRHGNPSLAA----FRKIPQC
>UNS08050.1_GP4_Porcine_reproductive_and_respiratory_syndrome_virus_PRRSV2/CN/I9/2018
CLRHGNTASKA----FRKIPQC
>QWM97620.1_GP4_Porcine_reproductive_and_respiratory_syndrome_virus_PRRSV2/CN/X4831/2018
CLRHGNPSTAT----FRKIPQC
>UNS08010.1_GP4_Porcine_reproductive_and_respiratory_syndrome_virus_PRRSV2/CN/E9/2018
CLRHSNPSSAA----FRKISQC
>UNS08058.1_GP4_Porcine_reproductive_and_respiratory_syndrome_virus_PRRSV2/CN/L3/2021
CLRHGNTTSKA----LRKIPHC
>ARA72282.1_glycoprotein_4_Porcine_reproductive_and_respiratory_syndrome_virus_FJWQ16
CLRHSDTSSKT----FRKIPQC
>QWM97641.1_GP4_Porcine_reproductive_and_respiratory_syndrome_virus_PRRSV2/CN/X2998/2018
CLRHGNTPSAA----FRKIRQC
>UNH55715.1_GP4_Porcine_reproductive_and_respiratory_syndrome_virus
CLRHNNPPSAT----LRKIPQC
>ALD60222.1_glycosylated_membrane_protein_4_Porcine_reproductive_and_respiratory_syndrome_virus_NVDC-R38-
2014
CLRHGDSSSPT----IRKSSQC
>UNH55685.1_GP4_Porcine_reproductive_and_respiratory_syndrome_virus
CLRHNNPPSAT----FRKIPQC
>UNH55675.1_GP4_Porcine_reproductive_and_respiratory_syndrome_virus
CLRHNNPSSAA----FRKIPQC
>UNH55634.1_GP4_Porcine_reproductive_and_respiratory_syndrome_virus
CLRHGNPSPAA----FRKIPQC
>UNH55624.1_GP4_Porcine_reproductive_and_respiratory_syndrome_virus
CLRHSNPSSAA----FRKIPQC
>UNH55665.1_GP4_Porcine_reproductive_and_respiratory_syndrome_virus
CLRHNYPSSAA----FRKIPQC
>UNH55645.1_GP4_Porcine_reproductive_and_respiratory_syndrome_virus
CLRHSYPSSAA----FRKIPQC
>UNH55694.1_GP4_Porcine_reproductive_and_respiratory_syndrome_virus
CLRYNNPSSAA----LRKIPQC
>UNH55655.1_GP4_Porcine_reproductive_and_respiratory_syndrome_virus
CLRHSNPSPAA----FRKVPQC
>UNH55585.1_GP4_Porcine_reproductive_and_respiratory_syndrome_virus
CLRHNNPPLAT----FRKIPQC
>UNH55575.1_GP4_Porcine_reproductive_and_respiratory_syndrome_virus
CLRHDNPSSAT----FRKIPQC
>UNH55594.1_GP4_Porcine_reproductive_and_respiratory_syndrome_virus
CLRHNNPSSAA----LRKIPQC
>QCY58628.1_envelope_protein_Porcine_reproductive_and_respiratory_syndrome_virus
CLRHGDSSSPT----IRKSSQC
>UNA88864.1_GP4_Porcine_reproductive_and_respiratory_syndrome_virus
CLRPHGVSAAQEEIPFGKSSQC
>UIX56013.1_GP4_Porcine_reproductive_and_respiratory_syndrome_virus_2020-Acheng-1
CLRHNYPSSAA----FRKIPQC
>ABB18269.1_GP4_Porcine_reproductive_and_respiratory_syndrome_virus
CLRHGHSSSEA----TRKGRQC
>UIE30524.1_GP4_Porcine_reproductive_and_respiratory_syndrome_virus
CPQLRGASTTQESVASRKSSQC
>UJU86162.1_GP4_Porcine_reproductive_and_respiratory_syndrome_virus_JS2020
CLRHNYPTSAA----FRKIPQC
>UIE30540.1_GP4_Porcine_reproductive_and_respiratory_syndrome_virus
CSQPRRASTTPTAPSFRKSSQC
>QIE30681.1_GP4_Porcine_reproductive_and_respiratory_syndrome_virus_SD110-1608
CLRHGNSPSPA----IRKSSQC
>UIE30516.1_GP4_Porcine_reproductive_and_respiratory_syndrome_virus
CPQLRGASTTQESGASRKSPQC
>UIE30500.1_GP4_Porcine_reproductive_and_respiratory_syndrome_virus
CFQQSRVGGAQRQTRAKGPSQC
>UIE30508.1_GP4_Porcine_reproductive_and_respiratory_syndrome_virus
CPQLRGASTTQESGASRKSPQC
>UIE30492.1_GP4_Porcine_reproductive_and_respiratory_syndrome_virus
CHQPQRAPAAQGHI-FTTPSQC
>UIE30532.1_GP4_Porcine_reproductive_and_respiratory_syndrome_virus
CPQLRGASTTQESVASRKSSQC
>UIE30580.1_GP4_Porcine_reproductive_and_respiratory_syndrome_virus
CARPP----------TKSPQC
>UIE30556.1_GP4_Porcine_reproductive_and_respiratory_syndrome_virus
CV-----SATQRGALSSKSSQC

>UIE30564.1_GP4_Porcine_reproductive_and_respiratory_syndrome_virus
CV-----SATQRGVPPGKSSQC
>UIE30572.1_GP4_Porcine_reproductive_and_respiratory_syndrome_virus
CLQPHEASTTPGPGIVTKASQC
>UIE30484.1_GP4_Porcine_reproductive_and_respiratory_syndrome_virus
CSRPYEASATTN----ERPSQC
>UIE30428.1_GP4_Porcine_reproductive_and_respiratory_syndrome_virus
CLRPHGGAAAESPIYTRESSQC
>UIE30436.1_GP4_Porcine_reproductive_and_respiratory_syndrome_virus
CLRHYGVEAAQGQIHARGQSQC
>UIE30460.1_GP4_Porcine_reproductive_and_respiratory_syndrome_virus
CPQPHETSSAQGGAPLGKPSQC
>UIE30412.1_GP4_Porcine_reproductive_and_respiratory_syndrome_virus
CLQLHGSPATSRQNTTEKKSQC
>UIE30396.1_GP4_Porcine_reproductive_and_respiratory_syndrome_virus
CLRLRGTSTTQREAPPGKPSQC
>UIE30404.1_GP4_Porcine_reproductive_and_respiratory_syndrome_virus
CLQPHRGPTTSGQNITEKKPQC
>UIE30420.1_GP4_Porcine_reproductive_and_respiratory_syndrome_virus
CLRSGSTVPAA----ERKSSQC
>UIE30388.1_GP4_Porcine_reproductive_and_respiratory_syndrome_virus
CLQPHRASSAQEGISLRAPSQC
>UIE30468.1_GP4_Porcine_reproductive_and_respiratory_syndrome_virus
CLQLHQAKETLGRGAAVKAPQC
>UIE30476.1_GP4_Porcine_reproductive_and_respiratory_syndrome_virus
CSRPFEASAATN----KRPSQC
>UIE30364.1_GP4_Porcine_reproductive_and_respiratory_syndrome_virus
CLQLHRASSAQEGTSLRAPSQC
>UIE30452.1_GP4_Porcine_reproductive_and_respiratory_syndrome_virus
CLRHYRVEAAQGQIHARGQSQC
>UIE30332.1_GP4_Porcine_reproductive_and_respiratory_syndrome_virus
CFQSRGTSTTPAKAPLRKSSQC
>UIE30356.1_GP4_Porcine_reproductive_and_respiratory_syndrome_virus
CLQPHRASSAQEGTSLRAPSQC
>UIE30348.1_GP4_Porcine_reproductive_and_respiratory_syndrome_virus
CLQPHRASSAQEGTSLRTPSQC
>UIE30372.1_GP4_Porcine_reproductive_and_respiratory_syndrome_virus
CLQPHRASSAQEGTSLRAPSQC
>UIE30316.1_GP4_Porcine_reproductive_and_respiratory_syndrome_virus
CFQSRGASVTSTETSLRKSSQC
>UIE30324.1_GP4_Porcine_reproductive_and_respiratory_syndrome_virus
CFQSREASTTPAETSPRRSSQC
>UIE30292.1_GP4_Porcine_reproductive_and_respiratory_syndrome_virus
CLQPHRAEETSGRGTLMKASQC
>UIE30180.1_GP4_Porcine_reproductive_and_respiratory_syndrome_virus
CLQLHRAEETPGHG-IVKASQC
>UIE30284.1_GP4_Porcine_reproductive_and_respiratory_syndrome_virus
CLRPHGDTSAGNKIYTRGSSQC
>UIE30276.1_GP4_Porcine_reproductive_and_respiratory_syndrome_virus
CLRPQGGATAGSQVYTTGSSQC
>UIE30188.1_GP4_Porcine_reproductive_and_respiratory_syndrome_virus
CLQLHRAEETPGHG-IVKASQC
>UIE30220.1_GP4_Porcine_reproductive_and_respiratory_syndrome_virus
CLRPHGGAAAKSQIHTRESSQC
>UIE30244.1_GP4_Porcine_reproductive_and_respiratory_syndrome_virus
CLQPHRAPKTPGQSTVGKTSQC
>UIE30252.1_GP4_Porcine_reproductive_and_respiratory_syndrome_virus
CLQPHRAPKTPGQSTVGKTSQC
>UIE30196.1_GP4_Porcine_reproductive_and_respiratory_syndrome_virus
CLRPNRAEETSR----LKASQC
>UIE30228.1_GP4_Porcine_reproductive_and_respiratory_syndrome_virus
CLQPHRAPKTPRQSTVGKTSQC
>UIE30204.1_GP4_Porcine_reproductive_and_respiratory_syndrome_virus
CLQPDRARETPGSASVMKAPQC
>UIE30260.1_GP4_Porcine_reproductive_and_respiratory_syndrome_virus
CLHLHRTPTAQANSPFRKSSQC
>UIE30212.1_GP4_Porcine_reproductive_and_respiratory_syndrome_virus
CLQPDRAKETPGSASVMKAPQC
>UIE30084.1_GP4_Porcine_reproductive_and_respiratory_syndrome_virus
CLQSHKAEETLRHSTVLKASQC
>UIE30268.1_GP4_Porcine_reproductive_and_respiratory_syndrome_virus
CLQPHRASTTPGQSAVRKTSQC
>UIE30148.1_GP4_Porcine_reproductive_and_respiratory_syndrome_virus
CLQPHRAQETSGHI--VKASQC
>UIE30140.1_GP4_Porcine_reproductive_and_respiratory_syndrome_virus
CLQPHKAKEPSGPXTVTKASQC
>UIE30172.1_GP4_Porcine_reproductive_and_respiratory_syndrome_virus
CLQPPRAEETPGHGTVMKASQC
>UIE30132.1_GP4_Porcine_reproductive_and_respiratory_syndrome_virus
CLQPHKAKEPSKPDTVMKASQC
>UIE30156.1_GP4_Porcine_reproductive_and_respiratory_syndrome_virus
CLQPHRAEETSGHV--VKASQC
>UIE30116.1_GP4_Porcine_reproductive_and_respiratory_syndrome_virus
CLQPHKAKEPLEHDTVMKASQC
>UIE30124.1_GP4_Porcine_reproductive_and_respiratory_syndrome_virus
CLQPHKAKEPSRLNTVMKASQC
>UIE30092.1_GP4_Porcine_reproductive_and_respiratory_syndrome_virus
CLQPNRAKETLRGRAVMKASQC
>UIE30100.1_GP4_Porcine_reproductive_and_respiratory_syndrome_virus
CLQPHRAKETSGHGAVMKASQC

```
>UIE30108.1_GP4_Porcine_reproductive_and_respiratory_syndrome_virus
CLQPHRAKETSGHGAVMKASQC
>UIE30060.1_GP4_Porcine_reproductive_and_respiratory_syndrome_virus
CFQPHRAEETSRHV--MKASQC
>UIE30164.1_GP4_Porcine_reproductive_and_respiratory_syndrome_virus
CLQSHRAEKTSQHGTVMKASQC
>UIE30012.1_GP4_Porcine_reproductive_and_respiratory_syndrome_virus
CLRHHRVNAAQGQAYTRGPSQC
>UIE30052.1_GP4_Porcine_reproductive_and_respiratory_syndrome_virus
CLQSHKVEETAGHGTVMKASQC
>UIE30076.1_GP4_Porcine_reproductive_and_respiratory_syndrome_virus
CLQPHRAKEASEHSTVMKASQC
>UIE29980.1_GP4_Porcine_reproductive_and_respiratory_syndrome_virus
CLQSHRATAAQGTTVITKSSQC
>UIE29988.1_GP4_Porcine_reproductive_and_respiratory_syndrome_virus
CLRSHEAPAAQRGI-IRKSPQC
>UIE30028.1_GP4_Porcine_reproductive_and_respiratory_syndrome_virus
CLQPHKASAASGQVTVGKASQC
>UIE30068.1_GP4_Porcine_reproductive_and_respiratory_syndrome_virus
CFQPHRAEETSRHV--MKASQC
>UIE30004.1_GP4_Porcine_reproductive_and_respiratory_syndrome_virus
CPRYK-AEATRGRTHTRGPSQC
>UIE30036.1_GP4_Porcine_reproductive_and_respiratory_syndrome_virus
CLQPHEASASSKPATVRKASQC
>UIE30020.1_GP4_Porcine_reproductive_and_respiratory_syndrome_virus
CPRHG-VEATRRQVHTRGPSQC
>UIE30044.1_GP4_Porcine_reproductive_and_respiratory_syndrome_virus
CLQPHEVEEASGHGTVMKASQC
>UIE29964.1_GP4_Porcine_reproductive_and_respiratory_syndrome_virus
CLQPHRVAAEQGTTATRKSSQC
>UIE29996.1_GP4_Porcine_reproductive_and_respiratory_syndrome_virus
CLRPHRDPAAQGETVIRKLSQC
>UIE29908.1_GP4_Porcine_reproductive_and_respiratory_syndrome_virus
CL-----STTQREPTIRKSSQC
>UIE29948.1_GP4_Porcine_reproductive_and_respiratory_syndrome_virus
CLQLHRFASAQGTI-TRKSSQC
>UIE29940.1_GP4_Porcine_reproductive_and_respiratory_syndrome_virus
CL-----STTQQGPTIRKPSQC
>UIE29884.1_GP4_Porcine_reproductive_and_respiratory_syndrome_virus
CL-----STTQQGPTIRKSSQC
>UIE29892.1_GP4_Porcine_reproductive_and_respiratory_syndrome_virus
CL-----PTTQRGPAIRKSSQC
>UIE29916.1_GP4_Porcine_reproductive_and_respiratory_syndrome_virus
CL-----STTQREPTIRKSSQC
>UIE29956.1_GP4_Porcine_reproductive_and_respiratory_syndrome_virus
CLQPHRAPAAQGGTIIRKSSQC
>UIE29900.1_GP4_Porcine_reproductive_and_respiratory_syndrome_virus
CL-----PTTQRRPAIRKSSQC
>UIE29860.1_GP4_Porcine_reproductive_and_respiratory_syndrome_virus
CL-----QTAQRGSSFGKSSQC
>UIE29788.1_GP4_Porcine_reproductive_and_respiratory_syndrome_virus
CF-----STIQGGTHFRKSSQC
>UIE29972.1_GP4_Porcine_reproductive_and_respiratory_syndrome_virus
CLQSHRATAAQGTTVITKSSQC
>UIE29924.1_GP4_Porcine_reproductive_and_respiratory_syndrome_virus
CI-----STTQGGSTIRKSSQC
>UIE29868.1_GP4_Porcine_reproductive_and_respiratory_syndrome_virus
CL-----STTQRGSTIRKSSQC
>UIE29844.1_GP4_Porcine_reproductive_and_respiratory_syndrome_virus
CVRHHGVSTTQRSP-PEKSSQC
>UIE29828.1_GP4_Porcine_reproductive_and_respiratory_syndrome_virus
CVRHHGTSIAQMSP-LGKSSQC
>UIE29780.1_GP4_Porcine_reproductive_and_respiratory_syndrome_virus
CL-----STIQGRTYPRKASQC
>UIE29820.1_GP4_Porcine_reproductive_and_respiratory_syndrome_virus
CVRHHGASTAQMSP-PGRSSQC
>UIE29812.1_GP4_Porcine_reproductive_and_respiratory_syndrome_virus
CVRHHGASTAQMSP-PGRSSQC
>UIE29836.1_GP4_Porcine_reproductive_and_respiratory_syndrome_virus
CVRHHGASTTQVSP-FEKSSQC
>UIE29852.1_GP4_Porcine_reproductive_and_respiratory_syndrome_virus
CFQSHRASTAQGTTPLWRSSQC
>UIE29796.1_GP4_Porcine_reproductive_and_respiratory_syndrome_virus
CLRSYDASLTGASTPSRKLSQC
>UIE29772.1_GP4_Porcine_reproductive_and_respiratory_syndrome_virus
CL-----STTQRGTHVRKSSQC
>UIE29748.1_GP4_Porcine_reproductive_and_respiratory_syndrome_virus
CL-----STTQRGTHSRKSSQC
>UIE29804.1_GP4_Porcine_reproductive_and_respiratory_syndrome_virus
CVRHHGASTAQMSP-PGRSSQC
>UIE29708.1_GP4_Porcine_reproductive_and_respiratory_syndrome_virus
CL-----STTQGGTYFRKSSQC
>UIE29700.1_GP4_Porcine_reproductive_and_respiratory_syndrome_virus
CL-----STMQGGVYSRKSSQC
>UIE29740.1_GP4_Porcine_reproductive_and_respiratory_syndrome_virus
CL-----STTQRGTHFRKPSQC
>UIE29716.1_GP4_Porcine_reproductive_and_respiratory_syndrome_virus
CP-----STMQGGTYFRKSSQC
>QWK65112.1_GP4_Porcine_reproductive_and_respiratory_syndrome_virus
CLRHGNPSSAT----FRKVPQC
```

>QWK65096.1_GP4_Porcine_reproductive_and_respiratory_syndrome_virus
CLRHSNPSPAA----FRKIPQC
>UDF05893.1_GP4_Porcine_reproductive_and_respiratory_syndrome_virus_HBap4/2018
CLRHGDSSSPT----IRKSSQC
>UIE29764.1_GP4_Porcine_reproductive_and_respiratory_syndrome_virus
CL-----STTQRETRVRKSSQC
>QWM97627.1_GP4_Porcine_reproductive_and_respiratory_syndrome_virus_PRRSV2/CN/X9830/2018
CLRHGNTPSAA----FRKIRQC
>QWK65104.1_GP4_Porcine_reproductive_and_respiratory_syndrome_virus
CLRHSDPSLAA----FRKIPQC
>QJQ80106.1_GP4_Porcine_reproductive_and_respiratory_syndrome_virus_GDDX-2018
CLRHGDSSSPT----IRKSSQC
>AIE38324.1_GP4_Porcine_reproductive_and_respiratory_syndrome_virus
CLRHGDSSSPT----IRKSSQC
>QWM97679.1_GP4_Porcine_reproductive_and_respiratory_syndrome_virus_PRRSV2/CN/F1228/2017
CLRHGDSSSPT----IRKSSQC
>QWM97655.1_GP4_Porcine_reproductive_and_respiratory_syndrome_virus_PRRSV2/CN/X4839/2017
CLRHSNPSSAA----FRKIPQC
>QGP71536.1_GP4_Porcine_reproductive_and_respiratory_syndrome_virus
CL-----SAAQRSAPVIKSSQC
>QWM97703.1_GP4_Porcine_reproductive_and_respiratory_syndrome_virus_PRRSV2/CN/110713/2018
CLRHSNPSPAA----FRKIPQC
>QGP71526.1_GP4_Porcine_reproductive_and_respiratory_syndrome_virus
CSQPHERSGTRSSV-RGKPSQC
>QLQ34394.1_GP4_Porcine_reproductive_and_respiratory_syndrome_virus_FJDJQ-2018
CLRHSNPSPAA----FRKIPQC
>QGP71506.1_GP4_Porcine_reproductive_and_respiratory_syndrome_virus
CLQPHTGPAAQGTIPQAKRSQC
>QMX85157.1_GP4_Porcine_reproductive_and_respiratory_syndrome_virus
CSRPHEVSPSHAHAAFRKSSQC
>QMX85148.1_GP4_Porcine_reproductive_and_respiratory_syndrome_virus
CAQPREVSTTQTEI-FRKSSQC
>AGT59170.1_GP4_Porcine_reproductive_and_respiratory_syndrome_virus_GZ11-G1
CLRPYGVSATHENISFGKPSQC
>QGP71516.1_GP4_Porcine_reproductive_and_respiratory_syndrome_virus
CLRH-GASTTQNGPSLRKPSQC
>QPL11614.1_GP4_Porcine_reproductive_and_respiratory_syndrome_virus_PRRSV2/USA/Lab4
CLRYGHSPPEA----IRKSRQC
>QPN96135.1_GP4_Porcine_reproductive_and_respiratory_syndrome_virus
CLRHGNPSPAA----FRKVPQC
>QPL11623.1_GP4_Porcine_reproductive_and_respiratory_syndrome_virus_PRRSV2/USA/Lab2
CLRHSNPSPAA----FRKIPQC
>QPL11633.1_GP4_Porcine_reproductive_and_respiratory_syndrome_virus_PRRSV2/USA/NC134/2018
CLRHNNPSSAA----FRKIPQC
>AAR11530.1_GP4_Porcine_reproductive_and_respiratory_syndrome_virus_EuroPRRSV
CFRPHEVSATQREIPFRKSSQC
>QPL11599.1_GP4_Porcine_reproductive_and_respiratory_syndrome_virus_PRRSV2/USA/Lab7
CLRHRDSASEA----IRKIPQC
>QPK93585.1_GP4_Porcine_reproductive_and_respiratory_syndrome_virus
CLRYGNPSPAA----FRKIPQC
>QPK41167.1_GP4_Porcine_reproductive_and_respiratory_syndrome_virus
CLRHGNSSSAA----FRKVPQC
>QNL10575.1_GP4_Porcine_reproductive_and_respiratory_syndrome_virus_2153073_*1
CLRHGDSSSQT----IRKSSQC
>QNL10515.1_GP4_Porcine_reproductive_and_respiratory_syndrome_virus_1890826_*1
CLRHRDSASEA----IRKIPQC
>QNL10525.1_GP4_Porcine_reproductive_and_respiratory_syndrome_virus_1890826_*2
CLRHSNPSPAA----FRKIPQC
>QNL10535.1_GP4_Porcine_reproductive_and_respiratory_syndrome_virus_1952821_*1
CLRHRDPPSST----IRKSSQC
>QNL10545.1_GP4_Porcine_reproductive_and_respiratory_syndrome_virus_1952821_*2
CLRHRDSASET----IRKIPQC
>QNL10435.1_GP4_Porcine_reproductive_and_respiratory_syndrome_virus_2132295
CLRHGYSSSEA----FRKGRQC
>QNL10425.1_GP4_Porcine_reproductive_and_respiratory_syndrome_virus_2125706
CLRHGYSSSEA----IRKGRQC
>QNL10405.1_GP4_Porcine_reproductive_and_respiratory_syndrome_virus_2108698
CLRHGDSSSQA----IRKSSQC
>QNL10495.1_GP4_Porcine_reproductive_and_respiratory_syndrome_virus_2158115
CLRHGHSSSEA----PRKSRQC
>QNL10475.1_GP4_Porcine_reproductive_and_respiratory_syndrome_virus_2147100
CLRHGDSSSQT----IRKSSQC
>QNL10455.1_GP4_Porcine_reproductive_and_respiratory_syndrome_virus_2137961
CLRHGYSSSEA----IRKSRQC
>QNL10485.1_GP4_Porcine_reproductive_and_respiratory_syndrome_virus_2154774
CLRHGHSPSET----IRKSRQC
>QNL10415.1_GP4_Porcine_reproductive_and_respiratory_syndrome_virus_2109640
CVRHGHPPSEA----TRKVRQC
>QNL10365.1_GP4_Porcine_reproductive_and_respiratory_syndrome_virus_2101687
CVRHSHPPSEA----TRKVRQC
>QNL10345.1_GP4_Porcine_reproductive_and_respiratory_syndrome_virus_2092399
CLRHGHSPLEA----IRKSRQC
>QNL10465.1_GP4_Porcine_reproductive_and_respiratory_syndrome_virus_2146871
CLRHGYSSSEA----IRKSRQC
>QNL10395.1_GP4_Porcine_reproductive_and_respiratory_syndrome_virus_2108625
CLRHGYSTSEA----FRKSRQC
>QNL10355.1_GP4_Porcine_reproductive_and_respiratory_syndrome_virus_2101209
CLRHGYSPSAA----IRKSRQC
>QNL10325.1_GP4_Porcine_reproductive_and_respiratory_syndrome_virus_2089141
CLRRGHPPSEA----IRKSRQC

>QNL10305.1_GP4_Porcine_reproductive_and_respiratory_syndrome_virus_2087409
CLRHGHPPSEA----IRKSRQC
>QNL10385.1_GP4_Porcine_reproductive_and_respiratory_syndrome_virus_2104469
CLRHGNPSSPT----LRKSSQC
>QNL10315.1_GP4_Porcine_reproductive_and_respiratory_syndrome_virus_2087619
CLRHGHSSSEA----IRKSRQC
>QNL10245.1_GP4_Porcine_reproductive_and_respiratory_syndrome_virus_2075305
CLRHGNPPLAA----FRKIPQC
>QNL10215.1_GP4_Porcine_reproductive_and_respiratory_syndrome_virus_2072533
CLRHRDSASEA----IRKIPQC
>QNL10205.1_GP4_Porcine_reproductive_and_respiratory_syndrome_virus_2070378
CLRHDHSSSEA----TRKSRQC
>QNL10265.1_GP4_Porcine_reproductive_and_respiratory_syndrome_virus_2078481
CLRHRNPSSAA----FRKIPQC
>QNL10255.1_GP4_Porcine_reproductive_and_respiratory_syndrome_virus_2078279
CLRHGNPSSEA----TRKIRQC
>QNL10275.1_GP4_Porcine_reproductive_and_respiratory_syndrome_virus_2082089
CLRHGNPPLAT----FRKVPQC
>QNL10225.1_GP4_Porcine_reproductive_and_respiratory_syndrome_virus_2073509
CLRHGDSSSQT----IRKSSQC
>QNL10195.1_GP4_Porcine_reproductive_and_respiratory_syndrome_virus_2069767
CLRHRDSASEA----IRKIPQC
>QNL10125.1_GP4_Porcine_reproductive_and_respiratory_syndrome_virus_2059830
CLRHGDSSSQT----IRKSSQC
>QNL10175.1_GP4_Porcine_reproductive_and_respiratory_syndrome_virus_2067408
CLRHRDSASEA----IRKIPQC
>QNL10185.1_GP4_Porcine_reproductive_and_respiratory_syndrome_virus_2067423
CLRHGNSPSEA----TRKIRQC
>QNL10155.1_GP4_Porcine_reproductive_and_respiratory_syndrome_virus_2065929
CLGHGHPPSAA----IRKSRQC
>QNL10115.1_GP4_Porcine_reproductive_and_respiratory_syndrome_virus_2059368
CLRHGHSSPEA----IRKSRQC
>QNL10145.1_GP4_Porcine_reproductive_and_respiratory_syndrome_virus_2063747
CLRHGYSSSEA----IRKGRQC
>QNL10055.1_GP4_Porcine_reproductive_and_respiratory_syndrome_virus_2048380
CLRHGNSPSEA----TRKIRQC
>QNL10065.1_GP4_Porcine_reproductive_and_respiratory_syndrome_virus_2050445
CLRHGNPSSAT----LRKIPQC
>QNL10025.1_GP4_Porcine_reproductive_and_respiratory_syndrome_virus_2023323
CVGHGHSPPEA----IRKSRQC
>QNL10035.1_GP4_Porcine_reproductive_and_respiratory_syndrome_virus_2029734
CLRHGNPPPAA----LRKIPQC
>QNL10005.1_GP4_Porcine_reproductive_and_respiratory_syndrome_virus_2021685
CLRHGHSSSEA----PRKSRQC
>QNL10045.1_GP4_Porcine_reproductive_and_respiratory_syndrome_virus_2035290
CLRHGDSSSQT----IRKSSQC
>QNL09995.1_GP4_Porcine_reproductive_and_respiratory_syndrome_virus_2020590
CVRHGHSSPEA----THKSRQC
>QNL09955.1_GP4_Porcine_reproductive_and_respiratory_syndrome_virus_2016194
CLRHGNSPSEA----TRKIRQC
>QNL09935.1_GP4_Porcine_reproductive_and_respiratory_syndrome_virus_2012512
CLRHGHPPSEA----ARKVRQC
>QNL09835.1_GP4_Porcine_reproductive_and_respiratory_syndrome_virus_1978525
CLRHGDSSFPT----IRKSSQC
>QNL09965.1_GP4_Porcine_reproductive_and_respiratory_syndrome_virus_2017769
CLRYGHAPNEA----FRKIRQC
>QNL09945.1_GP4_Porcine_reproductive_and_respiratory_syndrome_virus_2013955
CLRHGNSPSEA----TRKIRQC
>QNL09805.1_GP4_Porcine_reproductive_and_respiratory_syndrome_virus_1966315
CLRHGHSPLEA----IRKSRQC
>QNL09855.1_GP4_Porcine_reproductive_and_respiratory_syndrome_virus_1982018
CLRHGYSSSEA----IRKSRQC
>QNL09845.1_GP4_Porcine_reproductive_and_respiratory_syndrome_virus_1980019
CLRHGNPPPAA----LRKIPQC
>QNL09795.1_GP4_Porcine_reproductive_and_respiratory_syndrome_virus_1961538
CLRHRDSASEA----IRKIPQC
>QNL09745.1_GP4_Porcine_reproductive_and_respiratory_syndrome_virus_1953024
CLRHGHSSSEA----TRKSRQC
>QNL09875.1_GP4_Porcine_reproductive_and_respiratory_syndrome_virus_1982353
CLRHGYSSTEA----TRKSRQC
>QNL09755.1_GP4_Porcine_reproductive_and_respiratory_syndrome_virus_1953769
CLRHGNPSPAA----FRKVPQC
>QNL09695.1_GP4_Porcine_reproductive_and_respiratory_syndrome_virus_1927781
CLRHGYSSFPT----IRKSSQC
>QNL09685.1_GP4_Porcine_reproductive_and_respiratory_syndrome_virus_1926452
CLRHGDPSSPT----LRKSSQC
>QNL09705.1_GP4_Porcine_reproductive_and_respiratory_syndrome_virus_1943176
CLRHRDPPSST----IRKSSQC
>QKK83011.1_GP4_Porcine_reproductive_and_respiratory_syndrome_virus
CLRHSNPSSTA----FRKIPQC
>QNL09675.1_GP4_Porcine_reproductive_and_respiratory_syndrome_virus_1425619
CLRHDYPPSEA----TRKSRQC
>QKK82997.1_GP4_Porcine_reproductive_and_respiratory_syndrome_virus
CLRHDNPSSAA----SRKIPQC
>QKX94660.1_GP4_Porcine_reproductive_and_respiratory_syndrome_virus
CLRHGDSPSPT----IRKSPQC
>QIW77235.1_GP4_Porcine_reproductive_and_respiratory_syndrome_virus
CLRHGNTTPET----FRKIRQC
>QKX94650.1_GP4_Porcine_reproductive_and_respiratory_syndrome_virus
CLRHSNTPLAA----LRKIPQC

```
>QKK83004.1_GP4_Porcine_reproductive_and_respiratory_syndrome_virus
CLRHGNSPLAA----TRKIPQC
>QIJ55870.1_GP4_Porcine_reproductive_and_respiratory_syndrome_virus_4306-2017-YL
CLRHGHQTPEA----FRKTRQC
>QIJ55845.1_GP4_Porcine_reproductive_and_respiratory_syndrome_virus_3391-2017-NT
CLRHGNTTSKA----FRKIRQC
>QIJ55885.1_GP4_Porcine_reproductive_and_respiratory_syndrome_virus_4510-2017-YL
CLRHGNTTSPT----FRKSRQC
>QIJ55850.1_GP4_Porcine_reproductive_and_respiratory_syndrome_virus_3433-2018-YL
CLRHGNATTEA----FRKIRQC
>QIJ55855.1_GP4_Porcine_reproductive_and_respiratory_syndrome_virus_3638-2017-YL
CLRHGDSSSKT----FRKIPQC
>QIJ55840.1_GP4_Porcine_reproductive_and_respiratory_syndrome_virus_3303-2017-NT
CLRHGNTTSET----FRKIRQC
>QIJ55880.1_GP4_Porcine_reproductive_and_respiratory_syndrome_virus_4436-2017-TC
CLRHGNPAPQT----FRKIRQC
>QIJ55860.1_GP4_Porcine_reproductive_and_respiratory_syndrome_virus_3908-2017-TY
CLRHGNTSPET----FRKISQC
>QIJ55875.1_GP4_Porcine_reproductive_and_respiratory_syndrome_virus_4359-2017-CH
CLRHGNTTFQT----FRKARQC
>QIJ55800.1_GP4_Porcine_reproductive_and_respiratory_syndrome_virus_2206-2017-PT
CLRHGNTTSQT----FRKIRQC
>QIN91215.1_GP4_Porcine_reproductive_and_respiratory_syndrome_virus
CLRPHEVSTAQENISFGKPSQC
>QIJ55865.1_GP4_Porcine_reproductive_and_respiratory_syndrome_virus_4242-2017-CH
CLRHGHSAHEA----FRKIPQC
>QIJ55760.1_GP4_Porcine_reproductive_and_respiratory_syndrome_virus_187-2016-TN
CLRHGNTASEA----LRKTPQC
>QIJ55770.1_GP4_Porcine_reproductive_and_respiratory_syndrome_virus_337-2016-PT
CLRHGNTSTEA----FRKIPQC
>QIJ55820.1_GP4_Porcine_reproductive_and_respiratory_syndrome_virus_2634-2017-ML
CLRHGNTTSAA----FRKASQC
>QIJ55825.1_GP4_Porcine_reproductive_and_respiratory_syndrome_virus_2890-2017-CH
CLRHGHTTPKA----FRKIPQC
>QIJ55740.1_GP4_Porcine_reproductive_and_respiratory_syndrome_virus_0020-2018-TY
CLRHGNTTSQT----FRKIRQC
>QIJ55755.1_GP4_Porcine_reproductive_and_respiratory_syndrome_virus_080-2016-PT
CLGHSNTASEA----LRKIPQC
>QIJ55750.1_GP4_Porcine_reproductive_and_respiratory_syndrome_virus_0047-2018-CH
CLRHRDPSSKA----FRKIPQC
>QIJ55765.1_GP4_Porcine_reproductive_and_respiratory_syndrome_virus_0215-2018-CH
CLRHSYSSSET----LRKIPQC
>QIJ55775.1_GP4_Porcine_reproductive_and_respiratory_syndrome_virus_0363-2018-TY
CLRHGNTSPEA----LRKIPHC
>QIJ55795.1_GP4_Porcine_reproductive_and_respiratory_syndrome_virus_2181-2017-PT
CFGRGNTTSKA----ISKIRQC
>QIJ55835.1_GP4_Porcine_reproductive_and_respiratory_syndrome_virus_2994-2017-CH
CLRYGNTTSKA----FRKTPQC
>QIJ55790.1_GP4_Porcine_reproductive_and_respiratory_syndrome_virus_1146-2018-TC
CLRHGNTTSQA----FRKARQC
>QIJ55780.1_GP4_Porcine_reproductive_and_respiratory_syndrome_virus_431-2016-PT
CLRHGNTTSKA----FHKISQC
>QIJ55805.1_GP4_Porcine_reproductive_and_respiratory_syndrome_virus_2223-2017-KS
CLRHGNTTSAA----FRKAPQC
>QIJ55815.1_GP4_Porcine_reproductive_and_respiratory_syndrome_virus_2592-2017-PT
CLRHGNTPTKA----FRKIPQC
>QIJ55830.1_GP4_Porcine_reproductive_and_respiratory_syndrome_virus_2964-2017-YL
CLRHRDPSSKA----FRKIPQC
>QIJ55810.1_GP4_Porcine_reproductive_and_respiratory_syndrome_virus_2415-2018-YL
CLRHGNTTSET----FRKIRQC
>QIJ55785.1_GP4_Porcine_reproductive_and_respiratory_syndrome_virus_0728-2018-CH
CLGHGNTSNKA----SRKIPQC
>QIC50035.1_GP4_Porcine_reproductive_and_respiratory_syndrome_virus_LNDZD10-1806
CLRYSNPSSAA----FRKIPQC
>QIC50046.1_GP4_Porcine_reproductive_and_respiratory_syndrome_virus_HLJZD30-1902
CLRHNNPSSAA----FRKIPQC
>QIE30671.1_GP4_Porcine_reproductive_and_respiratory_syndrome_virus_SDWH27-1710
CLRHGDSSSST----IRKSSQC
>QIC50058.1_GP4_Porcine_reproductive_and_respiratory_syndrome_virus_HLHDZD32-1901
CLRYNNPPSAA----FRKIPQC
>QIJ55745.1_GP4_Porcine_reproductive_and_respiratory_syndrome_virus_0037-2018-TP
CLRHGNTASKA----FHKIPQC
>QFP41201.1_GP4_Porcine_reproductive_and_respiratory_syndrome_virus
CLRHSNPPLAT----FRKIPQC
>QIC50068.1_GP4_Porcine_reproductive_and_respiratory_syndrome_virus_HLJZD22-1812
CLRHSNPPSAA----FRKIPQC
>AYD75408.1_GP4_Porcine_reproductive_and_respiratory_syndrome_virus_2_IA14737-2016
CLRHSNPSPAA----FRKIPQC
>AKS03932.1_GP4_Porcine_reproductive_and_respiratory_syndrome_virus_WSV
CLRHRNSASEA----IRKIPQC
>AGA16691.1_GP4_Porcine_reproductive_and_respiratory_syndrome_virus_SDA2
CLRHGDSSSPT----IRKIPQC
>ANP23843.1_GP4_Porcine_reproductive_and_respiratory_syndrome_virus_ISU01
CLRHSNPSSAA----FRKIPQC
>ALZ45091.1_GP4_Porcine_reproductive_and_respiratory_syndrome_virus
CLRHGYSSIEA----IRKSRQC
>QHA24476.1_GP4_Porcine_reproductive_and_respiratory_syndrome_virus
CLRHGNPSSAT----FRKVPQC
>QHA24420.1_GP4_Porcine_reproductive_and_respiratory_syndrome_virus
CLRHGNTPPTA----FRKIPQC
```

>QHA24436.1_GP4_Porcine_reproductive_and_respiratory_syndrome_virus
CLRHGNTPPTA----FRKIPQC
>QHA24444.1_GP4_Porcine_reproductive_and_respiratory_syndrome_virus
CLRHGDTPPTA----FRKIPQC
>QHA24460.1_GP4_Porcine_reproductive_and_respiratory_syndrome_virus
CLRHGNPSSAT----FRKVPQC
>QFP41145.1_GP4_Porcine_reproductive_and_respiratory_syndrome_virus
CIRSHKVPGSPEGASVRKTPQC
>QFP41037.1_GP4_Porcine_reproductive_and_respiratory_syndrome_virus
CFQPHEVSAASTKAPLRKSSQC
>QFP41100.1_GP4_Porcine_reproductive_and_respiratory_syndrome_virus
CFQPHEVSAASTNVHLRKPSQC
>QFP41055.1_GP4_Porcine_reproductive_and_respiratory_syndrome_virus
CFQPHEGSTASTEAPLRKSSQC
>ADA85031.1_GP4_Porcine_reproductive_and_respiratory_syndrome_virus_KP
CLRHGDSSSPT----IRKSSQC
>QFP41028.1_GP4_Porcine_reproductive_and_respiratory_syndrome_virus
CFQPHEGSTASTEAPLRKPSQC
>QFP41127.1_GP4_Porcine_reproductive_and_respiratory_syndrome_virus
CFQPYEVSAASTEAPLRKSSQC
>QFP41073.1_GP4_Porcine_reproductive_and_respiratory_syndrome_virus
CIRSHKVPGSPEGASVRKTPQC
>QFP41109.1_GP4_Porcine_reproductive_and_respiratory_syndrome_virus
CFQPREDSTTSTKASLRKSSQC
>QFP41091.1_GP4_Porcine_reproductive_and_respiratory_syndrome_virus
CFQPHEDSKSSTEAPLRKSSQC
>QFP40983.1_GP4_Porcine_reproductive_and_respiratory_syndrome_virus
CIRSHKVPGSPEKAPVRKTSQC
>QFP41118.1_GP4_Porcine_reproductive_and_respiratory_syndrome_virus
CFQPHEDSTPSTEAPLRKSSQC
>QFP41082.1_GP4_Porcine_reproductive_and_respiratory_syndrome_virus
CFQPHEVSTASTEAPVRKSSQC
>QFP41064.1_GP4_Porcine_reproductive_and_respiratory_syndrome_virus
CIRSHKVPGSPEGASVRKTPQC
>QFP41001.1_GP4_Porcine_reproductive_and_respiratory_syndrome_virus
CFRSHETSAAQEKIVVRKASQC
>QFP41046.1_GP4_Porcine_reproductive_and_respiratory_syndrome_virus
CFRSHETSAAQGKI-VRKASQC
>QFP40938.1_GP4_Porcine_reproductive_and_respiratory_syndrome_virus
CFQPHEVSTASTETPLRKSSQC
>QFP41019.1_GP4_Porcine_reproductive_and_respiratory_syndrome_virus
CFQPHEGSTASTEAPLRKSSQC
>QFP40929.1_GP4_Porcine_reproductive_and_respiratory_syndrome_virus
CSQPSEVSATHPVTPFRKSSQC
>QFP41010.1_GP4_Porcine_reproductive_and_respiratory_syndrome_virus
CFQPHEGSTASTEAPLRKSSQC
>QFP40974.1_GP4_Porcine_reproductive_and_respiratory_syndrome_virus
CFQPHEVSAASTEAPLRKSSQC
>QFP40911.1_GP4_Porcine_reproductive_and_respiratory_syndrome_virus
CFRSHKVPGSPEKAPVRKTSQC
>QFP40956.1_GP4_Porcine_reproductive_and_respiratory_syndrome_virus
CTGPRGVPAAPGTTRSKGSPQC
>QFP40992.1_GP4_Porcine_reproductive_and_respiratory_syndrome_virus
CFRSHETSTAQKTIAVRKASQC
>QFR38100.1_GP4_Porcine_reproductive_and_respiratory_syndrome_virus
CLRHGDSSSPT----IRKSSQC
>QDH06641.1_GP4_Porcine_reproductive_and_respiratory_syndrome_virus
CLRPHESSGAQSST-YGKPSQC
>QED58280.1_GP4_Porcine_reproductive_and_respiratory_syndrome_virus_CH-WH-2019-1
CLRHGNPPPAA----FRKIPQC
>QBO24028.1_GP4_Porcine_reproductive_and_respiratory_syndrome_virus
CLRHSNPPSAA----FRKIPQC
>QCZ41176.1_GP4_Porcine_reproductive_and_respiratory_syndrome_virus
CLRHGDSSSST----IRKSSQC
>QDB06349.1_GP4_Porcine_reproductive_and_respiratory_syndrome_virus
CAQRAEAS--------RKPSQC
>QBA57441.1_GP4_Porcine_reproductive_and_respiratory_syndrome_virus
CLRHGDSSPET----FRKIPQC
>QFP40920.1_GP4_Porcine_reproductive_and_respiratory_syndrome_virus
CFQPHEVSATSVATSFKKSSQC
>QFP40965.1_GP4_Porcine_reproductive_and_respiratory_syndrome_virus
CFQPHEVSAASTEAPLRKSSQC
>AYO89549.1_GP4_Porcine_reproductive_and_respiratory_syndrome_virus_SDZC-1609
CLRHSYPSPAA----FRKVPQC
>AYO89559.1_GP4_Porcine_reproductive_and_respiratory_syndrome_virus_TJZH-1607
CLRHGNPPVAA----FRKVPQC
>AYO89539.1_GP4_Porcine_reproductive_and_respiratory_syndrome_virus_SDQZ-1609
CLRHGNPPPAA----FRKIPQC
>AYO89529.1_GP4_Porcine_reproductive_and_respiratory_syndrome_virus_SD99-1606
CLRHSNPSPAA----FRKVPQC
>AYO89509.1_GP4_Porcine_reproductive_and_respiratory_syndrome_virus_SD-1602
CLRHGNPSPAA----FRKIPQC
>AYO89479.1_GP4_Porcine_reproductive_and_respiratory_syndrome_virus_HNJYH-1606
CLRHSNPSSAA----FRKIPQC
>AYO89449.1_GP4_Porcine_reproductive_and_respiratory_syndrome_virus_CY2-1604
CLRHSYPSSAA----FRKVPQC
>AYO89469.1_GP4_Porcine_reproductive_and_respiratory_syndrome_virus_HBFL-1604
CLRHSNPSPAT----FRKIPQC
>AYO89439.1_GP4_Porcine_reproductive_and_respiratory_syndrome_virus_CY1-1604
CLRHSNPSSAA----FRKIPQC

>AYO89459.1_GP4_Porcine_reproductive_and_respiratory_syndrome_virus_HNJYF-1606
CLRHSNPSSAA----FRKIPKC
>AYO89499.1_GP4_Porcine_reproductive_and_respiratory_syndrome_virus_SDQD-1604
CLRHGNPSPAA----VRKIPRC
>AYO89489.1_GP4_Porcine_reproductive_and_respiratory_syndrome_virus_LNCH-1604
CLRHSNPSPAA----FRKIPQC
>ARH13271.1_GP4_Porcine_reproductive_and_respiratory_syndrome_virus
CSRSYEVSTTENAI-SRKSSQC
>AYE88567.1_GP4_Porcine_reproductive_and_respiratory_syndrome_virus
CLRHGDSSFPT----IRKSSQC
>AYE88575.1_GP4_Porcine_reproductive_and_respiratory_syndrome_virus
CLRHGNSSPKT----FRKISQC
>AYE88582.1_GP4_Porcine_reproductive_and_respiratory_syndrome_virus
CLRHGNPSSEA----FRKIPQC
>AXV43780.1_GP4_Porcine_reproductive_and_respiratory_syndrome_virus_EuroViet-02
CLRPHGVSTAQEATSFGKPSQC
>AXV43798.1_GP4_Porcine_reproductive_and_respiratory_syndrome_virus_EuroViet-03
CLRPHGVSTAQENIPFGKPSQC
>NP_047410.1_GP4_Porcine_reproductive_and_respiratory_syndrome_virus
CLRHRDSASEA----IRKIPQC
>AYO89519.1_GP4_Porcine_reproductive_and_respiratory_syndrome_virus_SD53-1603
CLRHSNPSPAA----FRRVPQC
>AXF36035.1_GP4_Porcine_reproductive_and_respiratory_syndrome_virus
CLRHSNPSSAA----FRKVPQC
>AXF38028.1_GP4_Porcine_reproductive_and_respiratory_syndrome_virus
CLRHSNPSSAT----FRKIPQC
>ANP23913.1_GP4_Porcine_reproductive_and_respiratory_syndrome_virus_ISU21
CLRHSNPSSAA----FRKIPQC
>AXF38012.1_GP4_Porcine_reproductive_and_respiratory_syndrome_virus
CLRHGDASSST----IHKIPQC
>AXF38004.1_GP4_Porcine_reproductive_and_respiratory_syndrome_virus
CLRHSNSSSAA----FRKIPQC
>AWV50475.1_GP4_Porcine_reproductive_and_respiratory_syndrome_virus_USA/IN_Purdue/14067/2017
CLRHSNPSPAT----FRKVPQC
>AXF38020.1_GP4_Porcine_reproductive_and_respiratory_syndrome_virus
CLRHGDSSSPT----IRKIPQC
>AVA30167.1_GP4_Porcine_reproductive_and_respiratory_syndrome_virus_GDYDZZZ
CLRHGNPSSQT----FRKIPQC
>AXF36023.1_GP4_Porcine_reproductive_and_respiratory_syndrome_virus
CLRHGNPSSAA----FRKVPQC
>AQV08434.1_GP4_Porcine_reproductive_and_respiratory_syndrome_virus
CLRYGNSPLKA----IRKIPQC
>ATU47361.1_GP4_Porcine_reproductive_and_respiratory_syndrome_virus
CLRHRDSSSPT----IRKSPQC
>ATU47337.1_GP4_Porcine_reproductive_and_respiratory_syndrome_virus
CLRHSNPSSAT----FRKVPQC
>ATU47369.1_GP4_Porcine_reproductive_and_respiratory_syndrome_virus
CLRHGDSPTPT----IRKSSQC
>ATU47353.1_GP4_Porcine_reproductive_and_respiratory_syndrome_virus
CLRHGDSSSPT----IRKSSQC
>ARO11138.1_GP4_Porcine_reproductive_and_respiratory_syndrome_virus_HENXX-9
CLRHGDSSSPT----IRKSSQC
>ABR37301.1_GP4_Porcine_reproductive_and_respiratory_syndrome_virus_JXwn06
CLRHGDSPSPT----IRKSSQC
>ASK40170.1_GP4_Porcine_reproductive_and_respiratory_syndrome_virus
CLRHGNPSSET----FRKIPQC
>ARO16049.1_GP4_Porcine_reproductive_and_respiratory_syndrome_virus_HENZMD-10
CAQLRGASTTHPKI-FRTSSQC
>ARH13167.1_GP4_Porcine_reproductive_and_respiratory_syndrome_virus
CLRHGDSSSST----IRKSSQC
>ARH13183.1_GP4_Porcine_reproductive_and_respiratory_syndrome_virus
CLRHGDSSSPT----IRKSSQC
>ARH13175.1_GP4_Porcine_reproductive_and_respiratory_syndrome_virus
CLRHSYPSSAA----FRKIPQC
>ARH13247.1_GP4_Porcine_reproductive_and_respiratory_syndrome_virus
CLRHSNPSPAA----FRKIPQC
>ARH13231.1_GP4_Porcine_reproductive_and_respiratory_syndrome_virus
CLRHGDSSFPT----IRKSSQC
>ARH13215.1_GP4_Porcine_reproductive_and_respiratory_syndrome_virus
CLRRSNPPPAA----FRKIPQC
>ARH13191.1_GP4_Porcine_reproductive_and_respiratory_syndrome_virus
CLRHSNPSPAA----FRKIPQC
>ARH13207.1_GP4_Porcine_reproductive_and_respiratory_syndrome_virus
CLRHGDSSSPT----IRKSSQC
>ARH13223.1_GP4_Porcine_reproductive_and_respiratory_syndrome_virus
CLRHGDSSSPT----IRKSSQC
>ARH13079.1_GP4_Porcine_reproductive_and_respiratory_syndrome_virus
CLRHSNPSSAA----FRKIPQC
>ARH13111.1_GP4_Porcine_reproductive_and_respiratory_syndrome_virus
CLRHSNPSPAA----FRKIPQC
>ARH13143.1_GP4_Porcine_reproductive_and_respiratory_syndrome_virus
CLRHSNPSSAT----FRKIPQC
>ARH13087.1_GP4_Porcine_reproductive_and_respiratory_syndrome_virus
CLRHGDSSSPT----IRKISQC
>ADV78531.1_ORF4_Porcine_reproductive_and_respiratory_syndrome_virus_JXwn06-81c
CLRHGDSPSPT----IRKSSQC
>ARH13071.1_GP4_Porcine_reproductive_and_respiratory_syndrome_virus
CLRHGDSSSPT----IRKSSQC
>ARH13119.1_GP4_Porcine_reproductive_and_respiratory_syndrome_virus
CLRHGDSSSPT----IRKSSQC

>ARH13048.1_GP4_Porcine_reproductive_and_respiratory_syndrome_virus
CLRHGDSSSPT----IRKSSQC
>ARA67917.1_GP4_Porcine_reproductive_and_respiratory_syndrome_virus_FZ16A
CLRHGDSSSST----IRKSSQC
>AQW40846.1_GP4_Porcine_reproductive_and_respiratory_syndrome_virus_NVSL97-7895
CLRHGDSSSQT----IRKSSQC
>AQW40729.1_GP4_Porcine_reproductive_and_respiratory_syndrome_virus_NVSL97-7895
CLRHGDSSSQT----IRKSSQC
>AQW40724.1_GP4_Porcine_reproductive_and_respiratory_syndrome_virus_NVSL97-7895
CLRHGDSSSQT----IRKSSQC
>AQW40684.1_GP4_Porcine_reproductive_and_respiratory_syndrome_virus_NVSL97-7895
CLRHGDSSSQT----IRKSSQC
>AQW40670.1_GP4_Porcine_reproductive_and_respiratory_syndrome_virus_NVSL97-7895
CLRHGDSSSQT----IRKSSQC
>AQW40704.1_GP4_Porcine_reproductive_and_respiratory_syndrome_virus_NVSL97-7895
CLRHGDSSSQT----IRKSSQC
>AQW40606.1_GP4_Porcine_reproductive_and_respiratory_syndrome_virus_NVSL97-7895
CLRHGDSSSQT----IRKSSQC
>AQW40616.1_GP4_Porcine_reproductive_and_respiratory_syndrome_virus_NVSL97-7895
CLRHGDSSSQT----IRKSSQC
>AQW40591.1_GP4_Porcine_reproductive_and_respiratory_syndrome_virus_NVSL97-7895
CLRHGDSSSQT----IRKSSQC
>AQW40561.1_GP4_Porcine_reproductive_and_respiratory_syndrome_virus_NVSL97-7895
CLRHGDSSSQT----IRKSSQC
>AQW40621.1_GP4_Porcine_reproductive_and_respiratory_syndrome_virus_NVSL97-7895
CLRHGDSSSQT----IRKSSQC
>AQW40626.1_GP4_Porcine_reproductive_and_respiratory_syndrome_virus_NVSL97-7895
CLRHGDSSSQT----TRKSSQC
>AQW40477.1_GP4_Porcine_reproductive_and_respiratory_syndrome_virus_NVSL97-7895
CLRHGDSSSQT----IRKSSQC
>AQW40492.1_GP4_Porcine_reproductive_and_respiratory_syndrome_virus_NVSL97-7895
CLRHGDSSSQT----IRKSSQC
>AQW40482.1_GP4_Porcine_reproductive_and_respiratory_syndrome_virus_NVSL97-7895
CLRHGDSSSQT----IRKSSQC
>AQW40522.1_GP4_Porcine_reproductive_and_respiratory_syndrome_virus_NVSL97-7895
CLRHGDSSSQT----IRKSSQC
>AQW40532.1_GP4_Porcine_reproductive_and_respiratory_syndrome_virus_NVSL97-7895
CLRHGDSSSQT----IRKSSQC
>AQW40467.1_GP4_Porcine_reproductive_and_respiratory_syndrome_virus_NVSL97-7895
CLRHGDSSSQT----IRKSSQC
>AQW40388.1_GP4_Porcine_reproductive_and_respiratory_syndrome_virus_NVSL97-7895
CLRHGDSSSQT----IRKSSQC
>AQW40472.1_GP4_Porcine_reproductive_and_respiratory_syndrome_virus_NVSL97-7895
CLRHGDSSSQT----IRKSSQC
>AQW40507.1_GP4_Porcine_reproductive_and_respiratory_syndrome_virus_NVSL97-7895
CLRHGDSSSQT----IRKSSQC
>AQW40334.1_GP4_Porcine_reproductive_and_respiratory_syndrome_virus_NVSL97-7895
CLRHGDSSSQT----IRKSSQC
>AQW40418.1_GP4_Porcine_reproductive_and_respiratory_syndrome_virus_NVSL97-7895
CLRHGDSSSQT----IRKSSQC
>AQW40413.1_GP4_Porcine_reproductive_and_respiratory_syndrome_virus_NVSL97-7895
CLRHGDSSSQT----IRKSSQC
>AQW40330.1_GP4_Porcine_reproductive_and_respiratory_syndrome_virus_NVSL97-7895
CLRHGDSSSQT----IRKSSQC
>AQW40245.1_GP4_Porcine_reproductive_and_respiratory_syndrome_virus_NVSL97-7895
CLRHGDSSSQT----IRKSSQC
>AQW40393.1_GP4_Porcine_reproductive_and_respiratory_syndrome_virus_NVSL97-7895
CLRHGDSSSQT----IRKSSQC
>AQW40325.1_GP4_Porcine_reproductive_and_respiratory_syndrome_virus_NVSL97-7895
CLRHGDSSSQT----IRKSSQC
>AQW40280.1_GP4_Porcine_reproductive_and_respiratory_syndrome_virus_NVSL97-7895
CLRHGDSSSQT----IRKSSQC
>AQW40190.1_GP4_Porcine_reproductive_and_respiratory_syndrome_virus_NVSL97-7895
CLRHGDSSSQT----IRKSSQC
>AQW40205.1_GP4_Porcine_reproductive_and_respiratory_syndrome_virus_NVSL97-7895
CLRHGDSSSQT----IRKSSQC
>AQW40320.1_GP4_Porcine_reproductive_and_respiratory_syndrome_virus_NVSL97-7895
CLRHGDSSSQT----IRKSSQC
>AQW40220.1_GP4_Porcine_reproductive_and_respiratory_syndrome_virus_NVSL97-7895
CLRHGDSSSQT----IRKSSQC
>AQW40140.1_GP4_Porcine_reproductive_and_respiratory_syndrome_virus_NVSL97-7895
CPRHGDSSSQT----IRKSSQC
>AQW40111.1_GP4_Porcine_reproductive_and_respiratory_syndrome_virus_NVSL97-7895
CLRHGDSSSQT----IRKSSQC
>AQW40106.1_GP4_Porcine_reproductive_and_respiratory_syndrome_virus_NVSL97-7895
CLRHGDSSSQT----IRKSSQC
>AQW40041.1_GP4_Porcine_reproductive_and_respiratory_syndrome_virus_NVSL97-7895
CLRHGDSSSQT----IRKSSQC
>AQW40081.1_GP4_Porcine_reproductive_and_respiratory_syndrome_virus_NVSL97-7895
CLRHGDSPSQT----IRKSSQC
>AQW39997.1_GP4_Porcine_reproductive_and_respiratory_syndrome_virus_NVSL97-7895
CLRHGDSSSQT----IRKSSQC
>AQW40012.1_GP4_Porcine_reproductive_and_respiratory_syndrome_virus_NVSL97-7895
CLRHGDSSSQT----IRKSSQC
>AQW39947.1_GP4_Porcine_reproductive_and_respiratory_syndrome_virus_NVSL97-7895
CLRHGDSSSQT----IRKSSQC
>AQW39952.1_GP4_Porcine_reproductive_and_respiratory_syndrome_virus_NVSL97-7895
CLRHGDSSSQT----IRKSSQC
>AQW39967.1_GP4_Porcine_reproductive_and_respiratory_syndrome_virus_NVSL97-7895
CLRHGDSSSQT----IRKSSQC

>AQW39912.1_GP4_Porcine_reproductive_and_respiratory_syndrome_virus_NVSL97-7895
CLRHGDSSSQT----IRKSSQC
>AQW39857.1_GP4_Porcine_reproductive_and_respiratory_syndrome_virus_NVSL97-7895
CLRHGDSSSQT----IRKSSQC
>AQW40185.1_GP4_Porcine_reproductive_and_respiratory_syndrome_virus_NVSL97-7895
CLRHGDSSSQT----IRKSSQC
>AQW39867.1_GP4_Porcine_reproductive_and_respiratory_syndrome_virus_NVSL97-7895
CLRHGDSSSQT----IRKSSQC
>AQW39917.1_GP4_Porcine_reproductive_and_respiratory_syndrome_virus_NVSL97-7895
CLRHGDSSSQT----IRKSSQC
>AQW39862.1_GP4_Porcine_reproductive_and_respiratory_syndrome_virus_NVSL97-7895
CLRHGDSSSQT----IRKSSQC
>AQW39852.1_GP4_Porcine_reproductive_and_respiratory_syndrome_virus_NVSL97-7895
CLRHGDSSSQT----IRKSSQC
>AQW40002.1_GP4_Porcine_reproductive_and_respiratory_syndrome_virus_NVSL97-7895
CLRHGDSSSQT----IRKSSQC
>AQW39887.1_GP4_Porcine_reproductive_and_respiratory_syndrome_virus_NVSL97-7895
CLRHGDSSSQT----IRKSSQC
>AQV12797.1_GP4_Porcine_reproductive_and_respiratory_syndrome_virus_GSWW/CHA_2015
CLRHGDSSSPT----IRKSSQC
>AQQ80265.1_GP4_Porcine_reproductive_and_respiratory_syndrome_virus
CLRHGDSSFPT----IRKSSQC
>AQQ80263.1_GP4_Porcine_reproductive_and_respiratory_syndrome_virus
CLRHGDSSFPT----IRKSSQC
>AQQ80261.1_GP4_Porcine_reproductive_and_respiratory_syndrome_virus
CLRHGDSSSPT----IRKSSQC
>AKU79228.1_GP4_Porcine_reproductive_and_respiratory_syndrome_virus_14LY02-FJ
CLRHGDSSSPT----IRKSSQC
>AQQ80262.1_GP4_Porcine_reproductive_and_respiratory_syndrome_virus
CLRHGDSSSPT----IRKSSQC
>AQQ80269.1_GP4_Porcine_reproductive_and_respiratory_syndrome_virus
CLRHGDSSSPT----IRKSSQC
>ALZ45161.1_GP4_Porcine_reproductive_and_respiratory_syndrome_virus
CLRHGDSSSQT----IRKSSQC
>AQQ80264.1_GP4_Porcine_reproductive_and_respiratory_syndrome_virus
CLRHGDSSSPT----IRKSSQC
>ALZ45152.1_GP4_Porcine_reproductive_and_respiratory_syndrome_virus
CLRHRDSASEA----IRKIPQC
>ALZ45126.1_GP4_Porcine_reproductive_and_respiratory_syndrome_virus
CLRHRNSAPEA----LRKIPQC
>ALZ45143.1_GP4_Porcine_reproductive_and_respiratory_syndrome_virus
CLRYGDSSSPT----IRKSSQC
>ALZ45135.1_GP4_Porcine_reproductive_and_respiratory_syndrome_virus
CLRHGDPSSPA----IRKSSQC
>ALZ45117.1_GP4_Porcine_reproductive_and_respiratory_syndrome_virus
CFQPHRVPATQNGAPFKKSSQC
>ALZ45099.1_GP4_Porcine_reproductive_and_respiratory_syndrome_virus
CLRHGDSPSQT----IRKSPPC
>ACJ06992.1_GP4_Porcine_reproductive_and_respiratory_syndrome_virus_KNU-07
CLQPHRGSTTQQSVPYKKQSQC
>ACH86029.1_GP4_Porcine_reproductive_and_respiratory_syndrome_virus_JSyx
CLRHGDSSSPT----IRKSSQC
>AOW71946.1_GP4_Porcine_reproductive_and_respiratory_syndrome_virus
CLRHGDSSSPT----IRKSSQC
>AOW71955.1_GP4_Porcine_reproductive_and_respiratory_syndrome_virus
CLRHSNPSSAA----FRKISQC
>APD14040.1_GP4_Porcine_reproductive_and_respiratory_syndrome_virus
CLRHGNPPFAA----FRKIPQC
>ADM64631.1_GP4_Porcine_reproductive_and_respiratory_syndrome_virus_HLJHL
CLRHGDSSSPT----IRKSSQC
>ADD13435.1_GP4_Porcine_reproductive_and_respiratory_syndrome_virus_07V063
CLRHHRVTAAQGRIYTRGPSQC
>AEK75360.1_GP4_Porcine_reproductive_and_respiratory_syndrome_virus
CLRHGDSSSPT----IRKRSQC
>ABW91085.1_GP4_Porcine_reproductive_and_respiratory_syndrome_virus_Henan-1
CLRHGDSSSPT----IRKSSQC
>ABU55014.1_GP4_Porcine_reproductive_and_respiratory_syndrome_virus_NX06
CLRHGDSSSPT----IRKSSQC
>ADB44070.1_GP4_Porcine_reproductive_and_respiratory_syndrome_virus
CLRHGDSSSPT----IRKSSQC
>ADA85039.1_GP4_Porcine_reproductive_and_respiratory_syndrome_virus_TP_P60
CLRHVDSSSPT----IRKISQC
>ADA85047.1_GP4_Porcine_reproductive_and_respiratory_syndrome_virus_TP_P90
CLRHGDSSSPT----IRKIPQC
>ADD10726.1_GP4_Porcine_reproductive_and_respiratory_syndrome_virus_AH0701
CLRHGDSSSPT----IRKSSQC
>ADA85055.1_GP4_Porcine_reproductive_and_respiratory_syndrome_virus_YN9
CLRHGDSSSPT----IRKIPQC
>ACN93864.1_GP4_Porcine_reproductive_and_respiratory_syndrome_virus_JXA1_P10
CLRHGDSSSPT----IRKISQC
>ACG60106.2_GP4_Porcine_reproductive_and_respiratory_syndrome_virus
CLRHGDSSSPT----IRKSSQC
>ACN93848.1_GP4_Porcine_reproductive_and_respiratory_syndrome_virus_JXA1_P70
CLRHGDSSSPT----IRKSPQC
>AAK20492.1_GP4_Porcine_reproductive_and_respiratory_syndrome_virus_16244B_2/18/97(Nebraska)pass.3
CLRHRDSASEA----IRKIPQC
>ACW82441.1_GP4_Porcine_reproductive_and_respiratory_syndrome_virus_SX-1
CLRHGDSSSPT----IRKSSQC
>AAK20520.1_GP4_Porcine_reproductive_and_respiratory_syndrome_virus_16244B_2/18/97(Nebraska)pass.3
CLRHRDSASEA----IRKIPQC

>ABV02056.1_GP4_Porcine_reproductive_and_respiratory_syndrome_virus_GD
CLRHGDSSSPT----IRKSSQC
>AAK20534.1_GP4_Porcine_reproductive_and_respiratory_syndrome_virus_16244B_2/18/97(Nebraska)pass.3
CLRHRDSASEA----IRKIPQC
>ANP24186.1_GP4_Porcine_reproductive_and_respiratory_syndrome_virus_SDSU26
CLGHGYSSSEA----VRKSRQC
>ANP24172.1_GP4_Porcine_reproductive_and_respiratory_syndrome_virus_SDSU58
CLRHGNSPPEA----VRKSRQC
>ACZ57978.1_GP4_Porcine_reproductive_and_respiratory_syndrome_virus
CLRHGDSSSPT----IRKIPQC
>ANP24123.1_GP4_Porcine_reproductive_and_respiratory_syndrome_virus_ISU90
CLRHGYSSSEA----FRKSRQC
>AAK20471.1_GP4_Porcine_reproductive_and_respiratory_syndrome_virus_16244B_2/18/97(Nebraska)pass.3
CLRHRDSASEA----IRKIPQC
>ANP24165.1_GP4_Porcine_reproductive_and_respiratory_syndrome_virus_SDSU60
CLRHNNPSSAA----FRKIPQC
>ANP24179.1_GP4_Porcine_reproductive_and_respiratory_syndrome_virus_SDSU62
CLGHGNSPPEA----VRKSRQC
>ANP24095.1_GP4_Porcine_reproductive_and_respiratory_syndrome_virus_ISU82
CLRHGHQTPET----IGKSRQC
>ANP24081.1_GP4_Porcine_reproductive_and_respiratory_syndrome_virus_ISU78
CLRHGDAPSAA----FRKIPQC
>ANP24193.1_GP4_Porcine_reproductive_and_respiratory_syndrome_virus_SDSU47
CLRHGYSSSEA----FRKSRQC
>ANP24032.1_GP4_Porcine_reproductive_and_respiratory_syndrome_virus_ISU67
CLRHSNPSSAA----FRKIPQC
>ANP24039.1_GP4_Porcine_reproductive_and_respiratory_syndrome_virus_ISU68
CLRHGDPPSEA----LRKSRQC
>ANP24046.1_GP4_Porcine_reproductive_and_respiratory_syndrome_virus_ISU69
CLRHSNPSFAA----FRKIPQC
>ANP24018.1_GP4_Porcine_reproductive_and_respiratory_syndrome_virus_ISU49
CLRHSNPPPAA----FRKIPQC
>ANP23976.1_GP4_Porcine_reproductive_and_respiratory_syndrome_virus_ISU32
CLRHGDSSSEA----TRKSRQC
>ANP23983.1_GP4_Porcine_reproductive_and_respiratory_syndrome_virus_ISU36
CLRHGNPSPAA----FRKVPQC
>ANP23969.1_GP4_Porcine_reproductive_and_respiratory_syndrome_virus_ISU30
CLRHSNPSPAA----FRKIPQC
>ANP23948.1_GP4_Porcine_reproductive_and_respiratory_syndrome_virus_ISU27
CLRHSNPSSAA----FRKIPQC
>ANP23857.1_GP4_Porcine_reproductive_and_respiratory_syndrome_virus_ISU03
CLRHSNPSSAA----FRKIPQC
>ANP23955.1_GP4_Porcine_reproductive_and_respiratory_syndrome_virus_ISU28
CLRHGYSSSEA----FRKSRQC
>ANP23899.1_GP4_Porcine_reproductive_and_respiratory_syndrome_virus_ISU17
CLRHNNPPSAA----FRKISQC
>ANP23829.1_GP4_Porcine_reproductive_and_respiratory_syndrome_virus_104194
CLRHGYSSSEA----IRKSRQC
>ANP23815.1_GP4_Porcine_reproductive_and_respiratory_syndrome_virus_101416
CLRYGNAPPAA----FRKIPQC
>ANP23808.1_GP4_Porcine_reproductive_and_respiratory_syndrome_virus_21675
CLRYGNAPPAA----FRKIPQC
>ANP23773.1_GP4_Porcine_reproductive_and_respiratory_syndrome_virus_14-78
CLRHGDSSSQT----IRKSSQC
>ANI06780.1_GP4_Porcine_reproductive_and_respiratory_syndrome_virus
CLRHSNPSSAA----FRKIPQC
>ALU09404.1_GP4_Porcine_reproductive_and_respiratory_syndrome_virus_GZgy15-1
CLRHGDSSSST----IRKSSQC
>ALU09387.1_GP4_Porcine_reproductive_and_respiratory_syndrome_virus_HNjz15
CLRHSIPSSAA----FRKIPQC
>ALU09380.1_GP4_Porcine_reproductive_and_respiratory_syndrome_virus_HNyc13
CLRHGDSSSPT----IRKSSQC
>ANP23766.1_GP4_Porcine_reproductive_and_respiratory_syndrome_virus_14-76
CLRHGDSSSQT----IRKSSQC
>ANP24074.1_GP4_Porcine_reproductive_and_respiratory_syndrome_virus_ISU73
CLRHGNSPSEA----TRKSRQC
>ALT10335.1_GP4_Porcine_reproductive_and_respiratory_syndrome_virus_HLJB1
CLRHGDSSSPT----IRKIPQC
>ANP24025.1_GP4_Porcine_reproductive_and_respiratory_syndrome_virus_ISU51
CLRHSNPSSAA----IRKIPQC
>ALU09396.1_GP4_Porcine_reproductive_and_respiratory_syndrome_virus_HNyc15
CLRHGNSPTKA----FRKIPQC
>ANP23738.1_GP4_Porcine_reproductive_and_respiratory_syndrome_virus_14-60
CLRHRDSASKA----IRKIPQC
>ALT10325.1_GP4_Porcine_reproductive_and_respiratory_syndrome_virus_HLJA1
CLRHGDSSSST----IRKIPQC
>ANP24088.1_GP4_Porcine_reproductive_and_respiratory_syndrome_virus_ISU81
CLRHGHQTPET----IGKSRQC
>ANP23822.1_GP4_Porcine_reproductive_and_respiratory_syndrome_virus_103837
CLRHGHSSSEA----IHKSRQC
>AGH70369.1_GP4_Porcine_reproductive_and_respiratory_syndrome_virus_NVDC-FJ
CARPHEAT--------RKPSQC
>AKU79220.1_GP4_Porcine_reproductive_and_respiratory_syndrome_virus_14LY01-FJ
CLRHGDSPSPT----IRKSSQC
>AGH70353.1_GP4_Porcine_reproductive_and_respiratory_syndrome_virus_NVDC-NM2
CARPHEAT--------RKPSQC
>AKZ66194.1_GP4_Porcine_reproductive_and_respiratory_syndrome_virus_JL580
CLRHGDSSSPT----IRKSSQC
>AKS03941.1_GP4_Porcine_reproductive_and_respiratory_syndrome_virus_HC120629
CLRHGNTSSKA----FRKTPQC

>AKS03923.1_GP4_Porcine_reproductive_and_respiratory_syndrome_virus_TY
CLRHRNSASEA----IRKIPQC
>AKS03914.1_GP4_Porcine_reproductive_and_respiratory_syndrome_virus_TD-2
CLRHGNTTSKA----FRKISQC
>AKS03869.1_GP4_Porcine_reproductive_and_respiratory_syndrome_virus_TD/TP
CLRYGNTTSKA----FRKIPQC
>AKS03896.1_GP4_Porcine_reproductive_and_respiratory_syndrome_virus_TD1
CLRHGNTSPET----FRKIPQC
>AKS03860.1_GP4_Porcine_reproductive_and_respiratory_syndrome_virus_Q94-136
CLRHGNTTSET----FRKIPQC
>AKS03887.1_GP4_Porcine_reproductive_and_respiratory_syndrome_virus_TY1
CLRHSNPPSEA----FRKIPQC
>AKS03851.1_GP4_Porcine_reproductive_and_respiratory_syndrome_virus_NT
CLRRSDPSSKA----FRKIPQC
>AKS03842.1_GP4_Porcine_reproductive_and_respiratory_syndrome_virus_M1
CLRHGDTTSKA----FRKIRQC
>AKS03878.1_GP4_Porcine_reproductive_and_respiratory_syndrome_virus_Tsai
CLRHGNPSSEA----IRKIPQC
>AKS03797.1_GP4_Porcine_reproductive_and_respiratory_syndrome_virus_660
CLRHGNTTSKA----FRKISQC
>AKS03833.1_GP4_Porcine_reproductive_and_respiratory_syndrome_virus_HL
CLRHGNTSSKT----FRKVPQC
>AKS03815.1_GP4_Porcine_reproductive_and_respiratory_syndrome_virus_CH
CLRHGNTSSKA----FRKVPQC
>AKS03824.1_GP4_Porcine_reproductive_and_respiratory_syndrome_virus_319
CLRHSNPASKA----IRKVPQC
>AKS03788.1_GP4_Porcine_reproductive_and_respiratory_syndrome_virus_310
CLRHGNTSSTA----FRKIPQC
>AKS03806.1_GP4_Porcine_reproductive_and_respiratory_syndrome_virus_803
CLRHGNTASET----IRKIPQC
>AKS03761.1_GP4_Porcine_reproductive_and_respiratory_syndrome_virus_JM
CLRHGNTTSKA----FRKIPQC
>AKS03905.1_GP4_Porcine_reproductive_and_respiratory_syndrome_virus_CH8V-J2
CLTHGNTTSEA----FRKIPQC
>AKS03779.1_GP4_Porcine_reproductive_and_respiratory_syndrome_virus_71-1
CLMHGNTTSTA----FRKISQC
>AKS03770.1_GP4_Porcine_reproductive_and_respiratory_syndrome_virus_PD119
CLRHRNSASEA----IRKIPQC
>AKS03716.1_GP4_Porcine_reproductive_and_respiratory_syndrome_virus_cw46
CLRHRNSASEA----IRKIPQC
>AKS03743.1_GP4_Porcine_reproductive_and_respiratory_syndrome_virus_HC120821-LL
CLRHGDTTSKA----FRKIRQC
>AKS03725.1_GP4_Porcine_reproductive_and_respiratory_syndrome_virus_HC120821-SH1
CLRHGNTAPEA----FRKIRQC
>AKS03734.1_GP4_Porcine_reproductive_and_respiratory_syndrome_virus_HC120821-SH2
CLRHGDATSQA----FRKIRQC
>AKS03752.1_GP4_Porcine_reproductive_and_respiratory_syndrome_virus_HC120904-CHYL
CLRHGDTTSKA----FRKVRQC
>AKS03680.1_GP4_Porcine_reproductive_and_respiratory_syndrome_virus_47-2
CLRHGNTTSTA----FRKIPQC
>AKS03689.1_GP4_Porcine_reproductive_and_respiratory_syndrome_virus_312
CLRHGNTSSTA----FRKIPQC
>AAC98533.1_envelope_protein_Porcine_reproductive_and_respiratory_syndrome_virus_MD001
CLRHGNPSSEA----IRKIPQC
>AKS03707.1_GP4_Porcine_reproductive_and_respiratory_syndrome_virus_338
CLRYGNTPHKA----ARKIPQC
>AKS03698.1_GP4_Porcine_reproductive_and_respiratory_syndrome_virus_1483
CLRHGNTTSKA----FRKIRQC
>AIP91955.1_GP4_Porcine_reproductive_and_respiratory_syndrome_virus
CLRHGDSSSQA----IRKSSQC
>AIZ09138.1_GP4_Porcine_reproductive_and_respiratory_syndrome_virus
CLRHGDSSSPT----IRKSSQC
>AIU94233.1_GP4_Porcine_reproductive_and_respiratory_syndrome_virus
CLRHGYSSSEA----FRKSRQC
>AIJ19283.1_GP4_Porcine_reproductive_and_respiratory_syndrome_virus
CLRHGDSSSPT----IRKIPQC
>AIZ09146.1_GP4_Porcine_reproductive_and_respiratory_syndrome_virus
CLRHGDSSSPT----IRKSSQC
>AIL31425.1_GP4_Porcine_reproductive_and_respiratory_syndrome_virus_LNEU12
CAQPREVSTTRSET-FGKSSQC
>AIJ19273.1_GP4_Porcine_reproductive_and_respiratory_syndrome_virus
CLRHGDSSSPT----IRKISQC
>AIJ19263.1_GP4_Porcine_reproductive_and_respiratory_syndrome_virus
CLRHGDSSTPT----IRKSSQC
>AIC75902.1_GP4_Porcine_reproductive_and_respiratory_syndrome_virus
CLRHGNSSFPT----IRKSSQC
>AIS76361.1_GP4_Porcine_reproductive_and_respiratory_syndrome_virus_NMG2014
CLRHGDSSSPT----IRKIPQC
>AIC75892.1_GP4_Porcine_reproductive_and_respiratory_syndrome_virus
CLRHGDSSTPT----IRKSSQC
>AHZ97944.1_GP4_Porcine_reproductive_and_respiratory_syndrome_virus
CLRHRDSASEA----IRKVPQC
>AHZ89312.1_GP4_Porcine_reproductive_and_respiratory_syndrome_virus
CLRHGDSSSPT----IRKSSQC
>AHZ96612.1_GP4_Porcine_reproductive_and_respiratory_syndrome_virus_HeNan-A9
CLRHGNSSSPT----IRKSSQC
>AHZ64926.1_GP4_Porcine_reproductive_and_respiratory_syndrome_virus_Henan-A7
CLRHGDSSSPT----IRKSSQC
>AGK45333.1_GP4_Porcine_reproductive_and_respiratory_syndrome_virus_755R
CLRHGHQAPEA----FRKIRQC

>AHZ64936.1_GP4_Porcine_reproductive_and_respiratory_syndrome_virus_Henan-A8
CLRHGDSSSPT----IRKSSQC
>AHZ64906.1_GP4_Porcine_reproductive_and_respiratory_syndrome_virus_Henan-A5
CLRHGDSSPPT----IRKSSQC
>AHZ64916.1_GP4_Porcine_reproductive_and_respiratory_syndrome_virus_Henan-A6
CLRHGDSSSPT----IRKSSQC
>AHZ64896.1_GP4_Porcine_reproductive_and_respiratory_syndrome_virus_Henan-A4
CLRHGESPSPT----IRKSSQC
>AHW58236.1_GP4_Porcine_reproductive_and_respiratory_syndrome_virus
CLRHGDSSSPA----IRKVSQC
>AGW31703.1_GP4_Porcine_reproductive_and_respiratory_syndrome_virus
CLRHGDSSSKT----FRKIPQC
>AGW31695.1_GP4_Porcine_reproductive_and_respiratory_syndrome_virus
CLRHSNPSSET----FRKIPQC
>AGW31687.1_GP4_Porcine_reproductive_and_respiratory_syndrome_virus
CLRHSNPSSET----FRKIPQC
>AHW29568.1_GP4_Porcine_reproductive_and_respiratory_syndrome_virus
CLRHGDPSSPT----IRKSSQC
>AGW31647.1_GP4_Porcine_reproductive_and_respiratory_syndrome_virus
CLRPHRVSTAQEAIPYGKSSQC
>AHM88228.1_GP4_Porcine_reproductive_and_respiratory_syndrome_virus_Henan-A3
CLRHGESPSPT----IRKSSQC
>AGW31655.1_GP4_Porcine_reproductive_and_respiratory_syndrome_virus
CVRSHRAS--------NKPSQC
>AGW31671.1_GP4_Porcine_reproductive_and_respiratory_syndrome_virus
CARPHGTL--------GKPSQC
>AGW31663.1_GP4_Porcine_reproductive_and_respiratory_syndrome_virus
CAQSHVAS--------SKPSQC
>AGW31679.1_GP4_Porcine_reproductive_and_respiratory_syndrome_virus
CLRHSNPSSET----FRKIPQC
>AHL83475.1_GP4_Porcine_reproductive_and_respiratory_syndrome_virus_HeNan-A2
CLRHGDASSST----IHKIPQC
>AGW23406.1_GP4_Porcine_reproductive_and_respiratory_syndrome_virus
CLRPHGVSTAQENISFGKPSQC
>AGQ55910.1_GP4_Porcine_reproductive_and_respiratory_syndrome_virus
CLRHGDSSSPT----IRKSSQC
>AGQ55886.1_GP4_Porcine_reproductive_and_respiratory_syndrome_virus
CLRHGDSSSPT----IRKSSQC
>AGH12419.1_GP4_Porcine_reproductive_and_respiratory_syndrome_virus_NVDC-NM1-2011
CARPRTASTTHSTI-FGTSSQC
>AGQ55902.1_GP4_Porcine_reproductive_and_respiratory_syndrome_virus
CLRHGDSSSPT----IRKSSQC
>AGQ55878.1_GP4_Porcine_reproductive_and_respiratory_syndrome_virus
CLRHGDSSSPT----IRKISQC
>AGQ55894.1_GP4_Porcine_reproductive_and_respiratory_syndrome_virus
CLRHGDSSSST----IRKSSQC
>ACV95343.1_GP4_Porcine_reproductive_and_respiratory_syndrome_virus_JXM100
CLRHGDSSSPT----IRKSSQC
>AFJ54168.1_GP4_Porcine_reproductive_and_respiratory_syndrome_virus_JXA1-P170
CLRHGDSSSPT----IRKSSQC
>AFU75327.1_GP4_Porcine_reproductive_and_respiratory_syndrome_virus_SD23983
CLRHRNSAPEA----LRKIPQC
>AGA16699.1_GP4_Porcine_reproductive_and_respiratory_syndrome_virus_SDA3
CLRHGDSSSPT----IRKIPQC
>AFK09063.1_GP4_Porcine_reproductive_and_respiratory_syndrome_virus_10-10FUJ-4
CLRHGDSSSPT----IRKSSQC
>ACV91361.1_GP4_Porcine_reproductive_and_respiratory_syndrome_virus_JXM40
CLRHGDSSSPT----IRKSSQC
>AFK09167.1_GP4_Porcine_reproductive_and_respiratory_syndrome_virus_10-10GX-5
CLRHGDSSSPT----IRKSSQC
>AFK09103.1_GP4_Porcine_reproductive_and_respiratory_syndrome_virus_10-10JL
CLRHGDSSSPT----IRKSSQC
>AFN88228.1_GP4_Porcine_reproductive_and_respiratory_syndrome_virus_VR2385
CLRHRNSASEA----IRKVPQC
>AFK09135.1_GP4_Porcine_reproductive_and_respiratory_syndrome_virus_10-10GX-1
CLRHGDSSSPT----IRKSSQC
>AFK09047.1_GP4_Porcine_reproductive_and_respiratory_syndrome_virus_10-10FUJ-2
CLRHGDSSSPT----IRKSSQC
>AFK08999.1_GP4_Porcine_reproductive_and_respiratory_syndrome_virus_10-10BJ-1
CLRHGDSPSPT----IRKSSQC
>ADD13447.1_GP4_Porcine_reproductive_and_respiratory_syndrome_virus_08V204
CLRPYRTNTTQG----KVPSQC
>ADG26793.1_GP4_Porcine_reproductive_and_respiratory_syndrome_virus_Shaanxi-2
CLRHGDSSSPT----IRESSQC
>AFJ92655.1_GP4_Porcine_reproductive_and_respiratory_syndrome_virus_NVDC-GD2-2011
CLRHGDSSSPT----IRKSSQC
>ADH94283.1_GP4_Porcine_reproductive_and_respiratory_syndrome_virus_FJ0604
CLRHGDSSSPT----IRKISQC
>ADD13441.1_GP4_Porcine_reproductive_and_respiratory_syndrome_virus_08V194
CPQTGASAS------FRKSPQC
>ABO87436.1_GP4_synthetic_construct
CLRHRDSASEA----IRKIPQC
>ACZ57962.1_GP4_Porcine_reproductive_and_respiratory_syndrome_virus
CLRHGDSSLPT----IRKSSQC
>ADG26787.1_GP4_Porcine_reproductive_and_respiratory_syndrome_virus_Shaanxi-1
CLRHGDSSPPT----VRKIPQC
>ACQ90714.1_GP4_Porcine_reproductive_and_respiratory_syndrome_virus_BJSD
CLRHGDSSSPT----IRKSSQC
>AAB93978.1_GP4_Porcine_reproductive_and_respiratory_syndrome_virus_IAF-Klop
CLRHGYSSSET----IRKIPQC

>CAA11093.1_gp4_Porcine_reproductive_and_respiratory_syndrome_virus
CLRHRDSASEA----IRKIPQC
>CAA11081.1_gp4_Porcine_reproductive_and_respiratory_syndrome_virus
CARPHGASEESQSVTFNKPSQC
>YP_009667151.1_hypothetical_protein_Lelystad_virus
CFRPHGVSAAQEKISFGKSSQC
>XVM46139.1_envelope_glycoprotein_GP4_Porcine_reproductive_and_respiratory_syndrome_virus_SC202403
CLRHGNPPLAA----FRKIPQC
>XVM46121.1_envelope_glycoprotein_GP4_Porcine_reproductive_and_respiratory_syndrome_virus_SC202401
CLRHRDSASEA----IRKIPQC
>QXE39836.1_ORF4_Porcine_reproductive_and_respiratory_syndrome_virus
CLRHNYPSSAA----FRKTPQC
>ACZ58649.1_glycosylated_membrane_protein_GP4_Porcine_reproductive_and_respiratory_syndrome_virus_WUH2
CLRHGDSSSPT----IRKSPQC
>XVM46130.1_envelope_glycoprotein_GP4_Porcine_reproductive_and_respiratory_syndrome_virus_SC202402
CLRHGNPPSAA----FRKVPQC
>XQJ32732.1_GP4_envelope_protein_Porcine_reproductive_and_respiratory_syndrome_virus
CLRHNYSTSAA----FRKIPQC
>CAA11099.1_gp4_Porcine_reproductive_and_respiratory_syndrome_virus
CLRHRDSASEA----IRKIPQC
>XQJ32724.1_GP4_envelope_protein_Porcine_reproductive_and_respiratory_syndrome_virus
CLRHSYPASAA----FRKVPQC
>XQJ32740.1_GP4_envelope_protein_Porcine_reproductive_and_respiratory_syndrome_virus
CLRHGDSPSST----IRKRPQC
>XMH29555.1_glycosylated_membrane_protein_GP4_Porcine_reproductive_and_respiratory_syndrome_virus_XM-2020
CLRHSNPSSAA----FRKISQC
>ABG75897.1_GP4_glycoprotein_Porcine_reproductive_and_respiratory_syndrome_virus_HB-1(3.9)
CLRHGDSSSPA----IRKSSQC
>XAJ10757.1_envelope_protein_GP4_Porcine_reproductive_and_respiratory_syndrome_virus
CLRHGNSPSSP----IRKSPQC
>XCA47556.1_envelope_protein_GP4_Porcine_reproductive_and_respiratory_syndrome_virus
CLRHNNPPPAA----FRKIPQC
>QGY98212.1_glycosylated_protein_4_Porcine_reproductive_and_respiratory_syndrome_virus
CLRHSNPSSAA----FRKIPQC
>XAJ10765.1_envelope_protein_GP4_Porcine_reproductive_and_respiratory_syndrome_virus
CLRHSNPSLAT----FRKIPQC
>XAJ10789.1_envelope_protein_GP4_Porcine_reproductive_and_respiratory_syndrome_virus
CLRHGNPSPAT----FRKIPQC
>XAJ10773.1_envelope_protein_GP4_Porcine_reproductive_and_respiratory_syndrome_virus
CLRHSNSSSPA----FRKVPQC
>USN23970.1_glycosylated_protein_4_Porcine_reproductive_and_respiratory_syndrome_virus
CLRHGHSSSEA----FRKSRQC
>XAJ10741.1_envelope_protein_GP4_Porcine_reproductive_and_respiratory_syndrome_virus
CLRHSNPSLAT----FRKIPQC
>QGY98502.1_glycosylated_protein_4_Porcine_reproductive_and_respiratory_syndrome_virus
CLRHGNPSPAA----FRKVPQC
>ATY35735.1_glycoprotein_4_Porcine_reproductive_and_respiratory_syndrome_virus_IN/2014/ISU-5
CLRHSDSSSEA----TRKSRQC
>WRU22339.1_envelope_glycoprotein_GP4_Porcine_reproductive_and_respiratory_syndrome_virus
CLRHGDASSPT----FRKIPQC
>WOF00751.1_GP4_protein_Porcine_reproductive_and_respiratory_syndrome_virus
CLRHSNPPLTA----FRKIPQC
>WLD47775.1_GP4_envelope_protein_Porcine_reproductive_and_respiratory_syndrome_virus_PRRSV-1/181187-2/2023
CLRPHESSGAQSST-YGKPSQC
>WKE35531.1_GP4_envelope_protein_Porcine_reproductive_and_respiratory_syndrome_virus
CLRHGDTSSSA----FRKIPQC
>WBU98356.1_envelope_protein_GP4_Porcine_reproductive_and_respiratory_syndrome_virus_GDHY0425
CLRRSDPSHPT----FRKVRQC
>XAJ10733.1_envelope_protein_GP4_Porcine_reproductive_and_respiratory_syndrome_virus
CLRHSNPSPAA----FRKIPQC
>WKE35522.1_GP4_envelope_protein_Porcine_reproductive_and_respiratory_syndrome_virus
CLRHGNATSSP----FRKIPQC
>WBU98364.1_envelope_protein_GP4_Porcine_reproductive_and_respiratory_syndrome_virus_GDYJ1224
CLRHGNASSST----IRKRSQC
>WBU98372.1_envelope_protein_GP4_Porcine_reproductive_and_respiratory_syndrome_virus_HN0713
CLRHGNPSPAA----FRKIPQC
>WKW83666.1_GP4_protein_Porcine_reproductive_and_respiratory_syndrome_virus
CLRHGNPSPAA----FRKIPQC
>WGV41568.1_glycosylated_envelope_protein_GP4_Porcine_reproductive_and_respiratory_syndrome_virus_PRRSV-CH-SDLY27-2022
CLRYSNPTPAA----FRKIPQC
>WBU98347.1_envelope_protein_GP4_Porcine_reproductive_and_respiratory_syndrome_virus_GDGZ0408
CLRHGNTSPKT----FRKIPQC
>UYC28834.1_GP4_protein_Porcine_reproductive_and_respiratory_syndrome_virus
CRRHGNSSSKA----FRKIPQC
>UXM19159.1_GP4_protein_Porcine_reproductive_and_respiratory_syndrome_virus
CLRYSNPSSAA----FRKVPQC
>UVW79518.1_GP4_envelope_protein_Porcine_reproductive_and_respiratory_syndrome_virus_CH/SCHY/2018
CLRHSNPSPAA----FRKIPQC
>UVW79534.1_GP4_envelope_protein_Porcine_reproductive_and_respiratory_syndrome_virus_CH/SCMS-3/2019
CLRHGDSSSPT----IRKSSQC
>UVW79502.1_GP4_envelope_protein_Porcine_reproductive_and_respiratory_syndrome_virus_CH/SCCD-2/2018
CLRHGNPSPAA----FRKIPQC
>UXM19168.1_GP4_protein_Porcine_reproductive_and_respiratory_syndrome_virus
CLRHGNSTSKA----FRKISQC
>UVW79566.1_GP4_envelope_protein_Porcine_reproductive_and_respiratory_syndrome_virus_CH/SCSN-1/2017
CLRHGDSSSPT----IRKSSQC
>UVW79590.1_GP4_envelope_protein_Porcine_reproductive_and_respiratory_syndrome_virus_CH/SCYB-2/2019
CLRYSNPSSAA----FRKIPQC
>UVW79582.1_GP4_envelope_protein_Porcine_reproductive_and_respiratory_syndrome_virus_CH/SCYB-1/2018

CLRHSNPSSAT----FRKVPQC
>UWL59108.1_GP4_protein_Porcine_reproductive_and_respiratory_syndrome_virus
CLRHGYSSSEA----FRKSRQC
>UVW79510.1_GP4_envelope_protein_Porcine_reproductive_and_respiratory_syndrome_virus_CH/SCGY-2/2020
CLTHRDSASEA----IRKIPQC
>UVW79550.1_GP4_envelope_protein_Porcine_reproductive_and_respiratory_syndrome_virus_CH/SCNC-2/2020
CLRHGNSSPKT----IRKIPQC
>UTT87573.1_GP4_protein_Porcine_reproductive_and_respiratory_syndrome_virus
CLRYGNSSSTT----FRKISQC
>QCY50788.1_glycosylated_membrane_protein_Porcine_reproductive_and_respiratory_syndrome_virus_SCya18
CLRHGNSSPKT----IRKIPQC
>UTT87547.1_GP4_protein_Porcine_reproductive_and_respiratory_syndrome_virus
CLRHGDSPSPT----IRKRSQC
>UTT87582.1_GP4_protein_Porcine_reproductive_and_respiratory_syndrome_virus
CLRHGNTSPKT----FRKIPQC
>UTT87609.1_GP4_protein_Porcine_reproductive_and_respiratory_syndrome_virus
CLRHGDSSSQA----FRKIPQC
>UTT87591.1_GP4_protein_Porcine_reproductive_and_respiratory_syndrome_virus
CLRHGDPSSPT----IRKSSQC
>UTT87538.1_GP4_protein_Porcine_reproductive_and_respiratory_syndrome_virus
CLRHGNSSPKT----IRKIPQC
>UTT87600.1_GP4_protein_Porcine_reproductive_and_respiratory_syndrome_virus
CLRHGDASVPT----IRKSPQC
>UTT87565.1_GP4_protein_Porcine_reproductive_and_respiratory_syndrome_virus
CLRHSDSSLAA----FRKIPQC
>UTT87556.1_GP4_protein_Porcine_reproductive_and_respiratory_syndrome_virus
CLRHGDSSSKT----FRKIPQC
>ALG62747.1_GP4_protein_Porcine_reproductive_and_respiratory_syndrome_virus
CLRHRDSTSKA----IRKIPQC
>URX38424.1_GP4_protein_Porcine_reproductive_and_respiratory_syndrome_virus
CLRHNYPSSAA----FRKIPQC
>QTW05959.1_ORF4_Porcine_reproductive_and_respiratory_syndrome_virus_2
CLRHNYPSSAA----FRKTPQC
>USO12773.1_glycoprotein_GP4_Porcine_reproductive_and_respiratory_syndrome_virus
CLRHGNSSSAA----FRKIPQC
>AFH96250.1_envelope_glycoprotein_GP4_Porcine_reproductive_and_respiratory_syndrome_virus_218-06
CFRLHGVPAAQKTNSFGKPSQC
>QGQ60124.1_GP4_protein_Porcine_reproductive_and_respiratory_syndrome_virus_2_USA/OklahomaSHM/2016
CLRHGHSSSEA----FRKSRQC
>AFH96244.1_envelope_glycoprotein_GP4_Porcine_reproductive_and_respiratory_syndrome_virus_231-05
CFRPHGVSAAQEKISFGKSSQC
>QIQ51126.1_GP4_protein_Porcine_reproductive_and_respiratory_syndrome_virus_NPUST-2789-3W-2
CFRPHGVSTAQENIPFGKPSQC
>ABU87651.1_GP4_envelope_protein_Porcine_reproductive_and_respiratory_syndrome_virus_QUAL1_Before
CLRHRDSASEA----IRKIPQC
>QCC26623.1_GP4_envelope_protein_Porcine_reproductive_and_respiratory_syndrome_virus_SWU/MS2/2018
CLRHSNSSSAT----FRKVPQC
>QCC26632.1_GP4_envelope_protein_Porcine_reproductive_and_respiratory_syndrome_virus_SWU/MS3/2018
CLRHSYSSSAA----FRKVPQC
>QCC26641.1_GP4_envelope_protein_Porcine_reproductive_and_respiratory_syndrome_virus_SWU/MY5/2018
CLRHSYPSSAA----FRKVPQC
>AXF35678.1_glycosylated_membrane_protein_GP4_Porcine_reproductive_and_respiratory_syndrome_virus_SCN17
CLRHGNPSSAA----FRKIPQC
>ACF49371.1_glycosylated_membrane_protein_GP4_Porcine_reproductive_and_respiratory_syndrome_virus_BJ
CLRHGDSSSPT----IRKSSQC
>ALD59926.1_glycosylated_membrane_protein_4_Porcine_reproductive_and_respiratory_syndrome_virus_NVDC-HeB1-2013
CLRHGDSSSST----IRKSSQC
>AKE49086.1_envelope_protein_GP4_Porcine_reproductive_and_respiratory_syndrome_virus
CLRHGDSSSPT----IRKSPQC
>ABU87643.1_GP4_envelope_protein_Porcine_reproductive_and_respiratory_syndrome_virus_MN30100
CLRHGDSSSQT----IRKSSQC
>ABW39366.1_glycosylated_membrane_protein_GP4_Porcine_reproductive_and_respiratory_syndrome_virus_WUH1
CLRHGDSSSPT----IRKSSQC
>AAF65937.1_ORF4_Porcine_reproductive_and_respiratory_syndrome_virus_SP
CLRHGDPSSAA----IRKSSQC
>ADC55001.1_glycosylated_membrane_protein_GP4_Porcine_reproductive_and_respiratory_syndrome_virus_GDQY2
CLRHGDSSSPT----IRKSSQC
>AEO92085.1_envelope_protein_GP4_Porcine_reproductive_and_respiratory_syndrome_virus_FS
CLRHGDSSSPT----IRKSSQC
>ACX33142.1_GP4_glycosylated_membrane_protein_Porcine_reproductive_and_respiratory_syndrome_virus_HN-HW
CLRHGDSSSPT----IRKISQC
>ACT33430.1_glycosylated_membrane_protein_GP4_Porcine_reproductive_and_respiratory_syndrome_virus_BJ0706
CLRHGDSSSPT----IRKSSQC
>ABL98050.1_GP4_envelope_protein_Porcine_reproductive_and_respiratory_syndrome_virus
CLRHRDSASEA----IRKIPQC
>ALZ45235.1_GP4_envelope_protein_Porcine_reproductive_and_respiratory_syndrome_virus_12-39404
CLRHGDPSTPA----IRKSSQC
>AKJ32571.1_envelope_glycoprotein_GP4_Porcine_reproductive_and_respiratory_syndrome_virus
CLRHRDSASEA----IRKIPQC
>AAK06817.1_GP4_protein_Porcine_reproductive_and_respiratory_syndrome_virus
CLRHGDPSFPA----IRKSSQC
>AKJ86983.1_glycosylated_membrane_protein_GP4_Porcine_reproductive_and_respiratory_syndrome_virus_HUN-2014
CLRHGDSSSPT----IRKSSQC
>AMM03239.1_glycosylated_membrane_protein_GP4_Porcine_reproductive_and_respiratory_syndrome_virus_SCwhn14DY
CLRHGDSSSPT----IRKSSQC
>ABS71989.1_GP4_envelope_protein_Porcine_reproductive_and_respiratory_syndrome_virus_Hn-1/06
CLRHGDSSSPT----IRKSSQC
>ALB08524.1_glycosylated_membrane_protein_GP4_Porcine_reproductive_and_respiratory_syndrome_virus
CLRHGDSSSPT----IRKSSQC

>ALB08497.1_glycosylated_membrane_protein_GP4_Porcine_reproductive_and_respiratory_syndrome_virus
CLRHGDSSSPT----IRKSSQC
>BAP16270.1_GP4_protein_Porcine_reproductive_and_respiratory_syndrome_virus_Yamagata10-7
CLRHSNPSPPA----FRKIPQC
>BAP16278.1_GP4_protein_Porcine_reproductive_and_respiratory_syndrome_virus_Aomori10-5
CLRHGNSSNQA----FRKIPQC
>AGJ51310.1_glycosylated_membrane_protein_GP4_Porcine_reproductive_and_respiratory_syndrome_virus
CLRHGDSSFPT----IRKSSQC
>ALB08506.1_glycosylated_membrane_protein_GP4_Porcine_reproductive_and_respiratory_syndrome_virus
CLRHGDSSSPT----IRKSSQC
>BAP16254.1_GP4_protein_Porcine_reproductive_and_respiratory_syndrome_virus_Nagasaki11-14
CLRHGNSPSAA----IRKSSQC
>AID16356.1_glycosylated_membrane_protein_GP4_Porcine_reproductive_and_respiratory_syndrome_virus
CLRHGDSSSPT----IRKSSQC
>BAP16262.1_GP4_protein_Porcine_reproductive_and_respiratory_syndrome_virus_Jam2
CLRHRDSASKA----IRKIPQC
>BAR72477.1_GP4_protein_Porcine_reproductive_and_respiratory_syndrome_virus_Jpn5-37
CLRHGHSPSET----IRKSRQC
>AJG42836.1_envelope_protein_GP4_Porcine_reproductive_and_respiratory_syndrome_virus
CLRHGDSSSPT----IRKSSQC
>ABU43170.1_GP4_envelope_protein_Porcine_reproductive_and_respiratory_syndrome_virus_Biss_After
CLRYGHSPPEA----TRKSRQC
>ABU43260.1_GP4_envelope_protein_Porcine_reproductive_and_respiratory_syndrome_virus_Hawkeye4_After
CLRHGYSSSEA----IRKSRQC
>ABU87659.1_GP4_envelope_protein_Porcine_reproductive_and_respiratory_syndrome_virus_QUAL2_After
CLRYGNSPHSA----ARKSSQC
>ABU43180.1_GP4_envelope_protein_Porcine_reproductive_and_respiratory_syndrome_virus_Biss_Before
CLRYGHSPSEA----TRKSRQC
>ABU43314.1_GP4_envelope_protein_Porcine_reproductive_and_respiratory_syndrome_virus_Lewis_Before
CLRHGHSPSEA----TRKSRQC
>ABU43278.1_GP4_envelope_protein_Porcine_reproductive_and_respiratory_syndrome_virus_Hawkeye7_After
CLRHGHSPSEA----TRKSRQC
>BAG49672.1_GP4_protein_Porcine_reproductive_and_respiratory_syndrome_virus_EDRD-1
CLRHGDSSPQT----IRKSRQC
>ABU43269.1_GP4_envelope_protein_Porcine_reproductive_and_respiratory_syndrome_virus_Hawkeye4_Before
CLRHGDSPSSA----IRKSPQC
>ABU43198.1_GP4_envelope_protein_Porcine_reproductive_and_respiratory_syndrome_virus_Flagship_Before
CLRHGYSSSEA----IRKSRQC
>ABU43323.1_GP4_envelope_protein_Porcine_reproductive_and_respiratory_syndrome_virus_MFF_After
CLRHGHSSSEA----TRKSRQC
>ABU43287.1_GP4_envelope_protein_Porcine_reproductive_and_respiratory_syndrome_virus_Hawkeye7_Before
CLRHGHSPSEA----TRKSRQC
>ABU43305.1_GP4_envelope_protein_Porcine_reproductive_and_respiratory_syndrome_virus_Lewis_After
CLRHGHSSSEA----TRKSRQC
>ABU43242.1_GP4_envelope_protein_Porcine_reproductive_and_respiratory_syndrome_virus_Hawkeye2_After
CLRYSNPSPAA----FRKIPQC
>AGW23842.1_glycosylated_protein_GP4_Porcine_reproductive_and_respiratory_syndrome_virus
CLRHGNPPHEA----FRKVSQC
>AGH14481.1_envelope_protein_GP4_Porcine_reproductive_and_respiratory_syndrome_virus
CLRHGNSTSKT----FRKIPQC
>ABU43296.1_GP4_envelope_protein_Porcine_reproductive_and_respiratory_syndrome_virus_ISU-P
CLRHRNSASEA----IRKIPQC
>AGI50448.1_envelope_protein_GP4_Porcine_reproductive_and_respiratory_syndrome_virus_11FS11-GD
CLRHGDSSSPA----IRKSSQC
>AGI50484.1_envelope_protein_GP4_Porcine_reproductive_and_respiratory_syndrome_virus_11GZ-GD
CLRHGDSSSPT----IRKSSQC
>AGI50376.1_envelope_protein_GP4_Porcine_reproductive_and_respiratory_syndrome_virus_10HN-GD
CLRHGDSSSST----IRKSSQC
>AGI50403.1_envelope_protein_GP4_Porcine_reproductive_and_respiratory_syndrome_virus_10FS1-GD
CLRHGNSSSPT----IRKSSQC
>AFZ78879.1_envelope_protein_GP4_Porcine_reproductive_and_respiratory_syndrome_virus_11SH-GD
CLRHGDSSSPT----IRKSSQC
>AFH96253.1_envelope_glycoprotein_GP4_Porcine_reproductive_and_respiratory_syndrome_virus_265-06
CRSP-TTQNES----FRKPPQC
>AGI50439.1_envelope_protein_GP4_Porcine_reproductive_and_respiratory_syndrome_virus_10ZQ-GD
CLRHGHSSSPT----IRKSSQC
>AFZ78888.1_envelope_protein_GP4_Porcine_reproductive_and_respiratory_syndrome_virus_11SH1-GD
CLRHGDSSSPT----IRKSSQC
>AFH96249.1_envelope_glycoprotein_GP4_Porcine_reproductive_and_respiratory_syndrome_virus_1133-05
CFRPHGVSAAQEKISFGKSSQC
>AFH96248.1_envelope_glycoprotein_GP4_Porcine_reproductive_and_respiratory_syndrome_virus_1132-05
CFRPHGVSAAQEKISFGKSSQC
>AFH96254.1_envelope_glycoprotein_GP4_Porcine_reproductive_and_respiratory_syndrome_virus_267-06
CRSPTTQNES-----FRKPPQC
>AFH96242.1_envelope_glycoprotein_GP4_Porcine_reproductive_and_respiratory_syndrome_virus_218-05
CRSPTTQNES-----FRKPPQC
>AFH96243.1_envelope_glycoprotein_GP4_Porcine_reproductive_and_respiratory_syndrome_virus_219-05
CLSPTPQSES-----FRKPPQC
>AFH96241.1_envelope_glycoprotein_GP4_Porcine_reproductive_and_respiratory_syndrome_virus_194-05
CLSPTTQKGN-----FRKPSQC
>ADO79966.1_envelope_glycoprotein_GP4_Porcine_reproductive_and_respiratory_syndrome_virus
CFRPHRVSAAQEKISFGKSSQC
>AFG30131.1_envelope_protein_GP4_Porcine_reproductive_and_respiratory_syndrome_virus_WUH4
CLRHGDSSSPT----IRKISQC
>AAA47104.1_ORF4_putative_Porcine_reproductive_and_respiratory_syndrome_virus
CFRPHGVSAAQEKISFGKSSQC
>ACD03123.1_envelope_protein_GP4_Porcine_reproductive_and_respiratory_syndrome_virus_XH-GD
SLRHGDSSSPT----IRKSSQC
>ADO79958.1_envelope_glycoprotein_GP4_Porcine_reproductive_and_respiratory_syndrome_virus
CFRPHGVSAAQKKISFGKSSQC

>AFH96240.1_envelope_glycoprotein_GP4_Porcine_reproductive_and_respiratory_syndrome_virus_1125-04
CSQLHRVSIAQGETFSNKPSQC
>ADO79946.1_envelope_glycoprotein_GP4_Porcine_reproductive_and_respiratory_syndrome_virus
CFRPHGVSAAQEKTSFGKSSQC
>ADO79942.1_envelope_glycoprotein_GP4_Porcine_reproductive_and_respiratory_syndrome_virus
CFRPHGVSAAQEKNSFGKSSQC
>ADO79954.1_envelope_glycoprotein_GP4_Porcine_reproductive_and_respiratory_syndrome_virus
CFRPHGVSAAQEKISFGKSSQC
>ADO79934.1_envelope_glycoprotein_GP4_Porcine_reproductive_and_respiratory_syndrome_virus
CFRPHEVSAAQEKISFGKSSQC
>ADO79938.1_envelope_glycoprotein_GP4_Porcine_reproductive_and_respiratory_syndrome_virus
CFRPHRVSAAQEKISFGKSSQC
>ACL97348.1_GP4_glycoprotein_Porcine_reproductive_and_respiratory_syndrome_virus_HeN-2
CLRHGDSPSPT----IRKSSQC
>ACJ71771.1_GP4_glycoprotein_Porcine_reproductive_and_respiratory_syndrome_virus
CLRHRNSASEA----IRKVPQC
>ADB04254.1_envelope_glycoprotein_GP4_Porcine_reproductive_and_respiratory_syndrome_virus
CLRHGDSSSPT----IRKSSQC
>ABY68566.1_glycosylated_minor_envelope_protein_GP4_Porcine_reproductive_and_respiratory_syndrome_virus_ST-
7
CLRHGDSSSPT----IRKSSQC
>ACF23009.1_glycosylated_minor_envelope_protein_GP4_Porcine_reproductive_and_respiratory_syndrome_virus_BD-
4
CLRHGDSSSPT----IRKSSQC
>ABO68997.1_envelope_glycoprotein_GP4_Porcine_reproductive_and_respiratory_syndrome_virus_HNyz
CLRHGDSSSPT----IRKISQC
>ABC86584.1_GP4_protein_Porcine_reproductive_and_respiratory_syndrome_virus
CLRHRDSASEA----IRKIPQC
>ABW35332.1_glycosylated_minor_envelope_protein_GP4_Porcine_reproductive_and_respiratory_syndrome_virus_GC-
2
CLRHGDSSSPT----IRKSSQC
>ABU94733.1_glycosylated_membrane_protein_GP4_Porcine_reproductive_and_respiratory_syndrome_virus_SHH
CLRHGDSSSPT----IRKSSQC
>ABB49058.1_GP4_glycoprotein_Porcine_reproductive_and_respiratory_syndrome_virus_FJ04A
CLRHRDSASEA----IRKIPQC
>ABF19770.1_envelope_glycoprotein_GP4_Porcine_reproductive_and_respiratory_syndrome_virus
CLRHGNSTSEA----FRKIPQC
>AAS78216.1_envelope_glycoprotein_GP4_Porcine_reproductive_and_respiratory_syndrome_virus
CLRHGDSSSQT----IRKSSQC
>ABO69002.1_envelope_glycoprotein_GP4_Porcine_reproductive_and_respiratory_syndrome_virus_HNsf
CLRHGDSSFPT----IRKIPQC
>AAX18235.1_GP4_envelope_glycoprotein_Porcine_reproductive_and_respiratory_syndrome_virus_FJ-1
CLRHSNTSPKT----FRKTPQC
>AAS78204.1_envelope_glycoprotein_GP4_Porcine_reproductive_and_respiratory_syndrome_virus
CLRHRNSASEA----TRKVPQC
>AAQ02937.1_GP4_glycoprotein_Porcine_reproductive_and_respiratory_syndrome_virus
CLRHRNPTPEA----FRKVPQC
>AAQ02943.1_GP4_glycoprotein_Porcine_reproductive_and_respiratory_syndrome_virus
CLRHRNPTPEA----FRKVPQC
>AAQ02925.1_GP4_glycoprotein_Porcine_reproductive_and_respiratory_syndrome_virus
CLRHRYSASEA----IRKVPQC
>AAQ02949.1_GP4_glycoprotein_Porcine_reproductive_and_respiratory_syndrome_virus
CLRHRNPTPEA----FRKVPQC
>AAQ02955.1_GP4_glycoprotein_Porcine_reproductive_and_respiratory_syndrome_virus
CLRHGDSSSPA----IRKSSQC
>AAQ02979.1_GP4_glycoprotein_Porcine_reproductive_and_respiratory_syndrome_virus
CLRHGDSSSQT----IRKSSQC
>AAS78210.1_envelope_glycoprotein_GP4_Porcine_reproductive_and_respiratory_syndrome_virus
CLRHGDSSPKT----IRKSSQC
>AAT01121.1_envelope_protein_GP4_Porcine_reproductive_and_respiratory_syndrome_virus
CLRHGDSSSPA----IRKSSQC
>AAR33012.1_glycoprotein_GP4_Porcine_reproductive_and_respiratory_syndrome_virus
CSRPHEASATQGQAPSRKSSQC
>AAD32104.1_envelope_protein_GP4_Porcine_reproductive_and_respiratory_syndrome_virus_MD-001_tw91
CLRHGNPSSEA----IRKIPQC
>AAR32998.1_glycoprotein_GP4_Porcine_reproductive_and_respiratory_syndrome_virus
CLRPYGASATEEKTSLRKSSQC

# PRRSV-2

>WOG33852.1_GP4_Porcine_reproductive_and_respiratory_syndrome_virus_2
LRHGNPPSQAIRKIPQ
>WOG33841.1_GP4_Porcine_reproductive_and_respiratory_syndrome_virus_2
LRHGNPPSQAIRKIPQ
>WMZ16716.1_GP4_Porcine_reproductive_and_respiratory_syndrome_virus_2
LRHGDSSSPTIRKSSQ
>WKV32960.1_GP4_Porcine_reproductive_and_respiratory_syndrome_virus_2
LRHGNPPSQAIRKIPQ
>UUS63670.1_GP4_Porcine_reproductive_and_respiratory_syndrome_virus_2
LRHSNPSSAAFRKISQ
>UJD73308.1_GP4_Porcine_reproductive_and_respiratory_syndrome_virus_2
LRHRDSASEAIRKIPQ
>UJD73298.1_GP4_Porcine_reproductive_and_respiratory_syndrome_virus_2
LRYRDSASEAIRKIPQ
>UJD73288.1_GP4_Porcine_reproductive_and_respiratory_syndrome_virus_2
LRHRDSASEAIRKIPQ
>UJD73278.1_GP4_Porcine_reproductive_and_respiratory_syndrome_virus_2
LRHRDSASEAIRKIPQ

>UJD73268.1_GP4_Porcine_reproductive_and_respiratory_syndrome_virus_2
LRHGNSPYPAIRKSSQ
>UJD73258.1_GP4_Porcine_reproductive_and_respiratory_syndrome_virus_2
LRHGNSPSPAIRKSSQ
>UJD73248.1_GP4_Porcine_reproductive_and_respiratory_syndrome_virus_2
LRHGNSPPPAIRKSSQ
>UJD73238.1_GP4_Porcine_reproductive_and_respiratory_syndrome_virus_2
LRHGNPSSAAFRKIPQ
>UJD73228.1_GP4_Porcine_reproductive_and_respiratory_syndrome_virus_2
LRHSNPSSTAFRKIPQ
>UJD73218.1_GP4_Porcine_reproductive_and_respiratory_syndrome_virus_2
LRHSNPPPAAFRKIPQ
>UJD73208.1_GP4_Porcine_reproductive_and_respiratory_syndrome_virus_2
LRHSNPPPATFRKIPQ
>UJD73198.1_GP4_Porcine_reproductive_and_respiratory_syndrome_virus_2
LRYSNPAPAAFRKVPQ
>UJD73188.1_GP4_Porcine_reproductive_and_respiratory_syndrome_virus_2
LRHSNPPPATFRKIPQ
>UJD73178.1_GP4_Porcine_reproductive_and_respiratory_syndrome_virus_2
LRHSNPPLATIHKVPQ
>UJD73168.1_GP4_Porcine_reproductive_and_respiratory_syndrome_virus_2
LRHSNPSSAAFRKIPQ
>UJD73158.1_GP4_Porcine_reproductive_and_respiratory_syndrome_virus_2
LRHSNPSPAAFRKVPQ
>UJD73148.1_GP4_Porcine_reproductive_and_respiratory_syndrome_virus_2
LRHGNPPFAAFRKVPQ
>UJD73138.1_GP4_Porcine_reproductive_and_respiratory_syndrome_virus_2
LRHGNPPSAAFRKVPQ
>UJD73128.1_GP4_Porcine_reproductive_and_respiratory_syndrome_virus_2
LRHSNPSSAAIRKVRQ
>UJD73118.1_GP4_Porcine_reproductive_and_respiratory_syndrome_virus_2
LRHSNPSSAAIRKVRQ
>UJD73108.1_GP4_Porcine_reproductive_and_respiratory_syndrome_virus_2
LRHSNPSPAAFRKVPQ
>UJD73098.1_GP4_Porcine_reproductive_and_respiratory_syndrome_virus_2
LRHSNPSPAAFRKVPQ
>UJD73088.1_GP4_Porcine_reproductive_and_respiratory_syndrome_virus_2
LRHSNPSPAAFRRVPQ
>UJD73078.1_GP4_Porcine_reproductive_and_respiratory_syndrome_virus_2
LRHSNPSPAAFRKVPQ
>UJD73068.1_GP4_Porcine_reproductive_and_respiratory_syndrome_virus_2
LRHSNPSPAAFRKVSQ
>UJD73058.1_GP4_Porcine_reproductive_and_respiratory_syndrome_virus_2
LRHSNPSPTAFRKVPQ
>UJD73048.1_GP4_Porcine_reproductive_and_respiratory_syndrome_virus_2
LRHSNPSPAAFRRVPQ
>UJD73038.1_GP4_Porcine_reproductive_and_respiratory_syndrome_virus_2
LRHGDSSSPTIRKSSQ
>UJD73028.1_GP4_Porcine_reproductive_and_respiratory_syndrome_virus_2
LRHGDSPYPTIRKSSQ
>UJD73018.1_GP4_Porcine_reproductive_and_respiratory_syndrome_virus_2
LRHGDSSSPTIRKSSQ
>UJD73008.1_GP4_Porcine_reproductive_and_respiratory_syndrome_virus_2
LRHGDSSSSTIRKSSQ
>UJD72998.1_GP4_Porcine_reproductive_and_respiratory_syndrome_virus_2
LRHGNSSSPTIRKSSQ
>UJD72988.1_GP4_Porcine_reproductive_and_respiratory_syndrome_virus_2
LRHGDSSFPTIRKSSQ
>UEE94920.1_GP4_Porcine_reproductive_and_respiratory_syndrome_virus_2
LRHGYAPSEAFRKSRQ
>QPB67503.1_GP4_Porcine_reproductive_and_respiratory_syndrome_virus_2
LRHGDSSSPTIRKSSQ
>QPB67495.1_GP4_Porcine_reproductive_and_respiratory_syndrome_virus_2
LRHGDSSSPTIRKSSQ
>QPB67487.1_GP4_Porcine_reproductive_and_respiratory_syndrome_virus_2
LRHGDSSFPTIRKSSQ
>QPB67479.1_GP4_Porcine_reproductive_and_respiratory_syndrome_virus_2
LRHGDSSSPTIRKSSQ
>QPB67471.1_GP4_Porcine_reproductive_and_respiratory_syndrome_virus_2
LRHGDSSFPTIRKIPQ
>QPB67463.1_GP4_Porcine_reproductive_and_respiratory_syndrome_virus_2
LRHGDSSSPTIRKSSQ
>QPB67455.1_GP4_Porcine_reproductive_and_respiratory_syndrome_virus_2
LRHGDSSSPTIRKSSQ
>QPB67447.1_GP4_Porcine_reproductive_and_respiratory_syndrome_virus_2
LRHGDSSFPTIRKSSQ
>QPB67439.1_GP4_Porcine_reproductive_and_respiratory_syndrome_virus_2
LRHGDSSSPTIRKSSQ
>QPB67431.1_GP4_Porcine_reproductive_and_respiratory_syndrome_virus_2
LRHGDSSSPTIRKSSQ
>QPB67423.1_GP4_Porcine_reproductive_and_respiratory_syndrome_virus_2
LRHGDSSSPTIRKSSQ
>QPB67415.1_GP4_Porcine_reproductive_and_respiratory_syndrome_virus_2
LRHGDSSSPTIRKSSQ
>QPB67407.1_GP4_Porcine_reproductive_and_respiratory_syndrome_virus_2
RRHGDSSSPTIRKSSQ
>QPB67399.1_GP4_Porcine_reproductive_and_respiratory_syndrome_virus_2
LRHGDSSSPTIRKSSQ
>QPB67391.1_GP4_Porcine_reproductive_and_respiratory_syndrome_virus_2
LRHGDSSSPTIRKSSQ

>QPB67383.1_GP4_Porcine_reproductive_and_respiratory_syndrome_virus_2
LRHGDSSSPTIRKSSQ
>QPB67375.1_GP4_Porcine_reproductive_and_respiratory_syndrome_virus_2
LRHGDSSSXTIRKSSQ
>QPB67367.1_GP4_Porcine_reproductive_and_respiratory_syndrome_virus_2
LRHGDSSSPTIRKSSQ
>QPB67359.1_GP4_Porcine_reproductive_and_respiratory_syndrome_virus_2
LRHGDSSFPTIRKSSQ
>QPB67351.1_GP4_Porcine_reproductive_and_respiratory_syndrome_virus_2
LRHGDSSSPTIRKSSQ
>QQX23413.1_GP4_Porcine_reproductive_and_respiratory_syndrome_virus_2
LRHGDSSSPTIRKSSQ
>QIC53176.1_GP4_Porcine_reproductive_and_respiratory_syndrome_virus_2
LRHSNPPSATFRKIPQ
>QIC53166.1_GP4_Porcine_reproductive_and_respiratory_syndrome_virus_2
LRHGDSSSPTIRKSSQ
>QIC53156.1_GP4_Porcine_reproductive_and_respiratory_syndrome_virus_2
LRHSNPSSTTFRKIPQ
>QIC53146.1_GP4_Porcine_reproductive_and_respiratory_syndrome_virus_2
LRHGDSSPPTIRKSSQ
>QIC53136.1_GP4_Porcine_reproductive_and_respiratory_syndrome_virus_2
LRHSNPSPAAFRKVPQ
>QIC53126.1_GP4_Porcine_reproductive_and_respiratory_syndrome_virus_2
LRHGDSSPSTIRKSSQ
>QGT31812.1_GP4_Porcine_reproductive_and_respiratory_syndrome_virus_2
LRHNNPSSAAFRKVPQ
>QGT31802.1_GP4_Porcine_reproductive_and_respiratory_syndrome_virus_2
LRHNNPTSAAFRKIPQ
>QDN53985.1_GP4_Porcine_reproductive_and_respiratory_syndrome_virus_2
LRHGDSSSPTIRKSSQ
>QDN53973.1_GP4_Porcine_reproductive_and_respiratory_syndrome_virus_2
LRHGDSSSPTIRKSSQ
>QDN53961.1_GP4_Porcine_reproductive_and_respiratory_syndrome_virus_2
LRHGDSSSPAIRKSSQ
>QDL52628.1_GP4_Porcine_reproductive_and_respiratory_syndrome_virus_2
LRHGDSSSPTIRKSSQ
>QDL52616.1_GP4_Porcine_reproductive_and_respiratory_syndrome_virus_2
LRHGDSSSPTIRKSSQ
>QCY51165.1_GP4_Porcine_reproductive_and_respiratory_syndrome_virus_2
LRHGDSSSPTIRKSSQ
>QCY51157.1_GP4_Porcine_reproductive_and_respiratory_syndrome_virus_2
LRHGDSSSETIRKSRQ
>AYD75408.1_GP4_Porcine_reproductive_and_respiratory_syndrome_virus_2
LRHSNPSPAAFRKIPQ
>WRW24808.1_glycoprotein_4_Porcine_reproductive_and_respiratory_syndrome_virus_2
LRHGDPSSAAIRKSSQ
>WRW24807.1_glycoprotein_4_Porcine_reproductive_and_respiratory_syndrome_virus_2
LRHRDSASEAIRKIPQ
>WRW24806.1_glycoprotein_4_Porcine_reproductive_and_respiratory_syndrome_virus_2
LRHRDSASTAFRKVPQ
>WRW24805.1_glycoprotein_4_Porcine_reproductive_and_respiratory_syndrome_virus_2
LRHRDSASEAIRKIPQ
>XOB74749.1_envelope_protein_GP4_Porcine_reproductive_and_respiratory_syndrome_virus_2
LRHGDSPSPTIRKSSQ
>XOB74741.1_envelope_protein_GP4_Porcine_reproductive_and_respiratory_syndrome_virus_2
LRHSNPSPATFRKVPQ
>XOB74733.1_envelope_protein_GP4_Porcine_reproductive_and_respiratory_syndrome_virus_2
LRHSNPSSAAFRKVPQ
>XOB74725.1_envelope_protein_GP4_Porcine_reproductive_and_respiratory_syndrome_virus_2
LRHGDSSFPAIRKSSQ
>XOB74717.1_envelope_protein_GP4_Porcine_reproductive_and_respiratory_syndrome_virus_2
LRHSNPSSAAFRKVSQ
>XJW05945.1_GP4_envelope_protein_Porcine_reproductive_and_respiratory_syndrome_virus_2
LRHGYSSSAALRKIPQ
>XJW05937.1_GP4_envelope_protein_Porcine_reproductive_and_respiratory_syndrome_virus_2
LRHNYSSSAAFRKIPQ
>WZL52762.1_GP4_envelope_protein_Porcine_reproductive_and_respiratory_syndrome_virus_2
LRYSNPSSAAFRKIPQ
>WZL52753.1_GP4_envelope_protein_Porcine_reproductive_and_respiratory_syndrome_virus_2
LRHGDSSSPTIRKSSQ
>WZL52745.1_GP4_envelope_protein_Porcine_reproductive_and_respiratory_syndrome_virus_2
LRHRDSASEAIRKIPQ
>WZL52737.1_GP4_envelope_protein_Porcine_reproductive_and_respiratory_syndrome_virus_2
LRHDYPTSAAFRKIPQ
>WOZ07421.1_GP4_envelope_protein_Porcine_reproductive_and_respiratory_syndrome_virus_2
LRHGDSSFPTIRKSSQ
>WOZ07411.1_GP4_envelope_protein_Porcine_reproductive_and_respiratory_syndrome_virus_2
LRHGDSSFPTIRKSSQ
>WOZ07401.1_GP4_envelope_protein_Porcine_reproductive_and_respiratory_syndrome_virus_2
LRHGDSSSSTIRKSSQ
>WOZ07391.1_GP4_envelope_protein_Porcine_reproductive_and_respiratory_syndrome_virus_2
LRHGDSSSQTIRKSSQ
>WOZ07381.1_GP4_envelope_protein_Porcine_reproductive_and_respiratory_syndrome_virus_2
LRHRDSASEAFRKVPQ
>WOZ07371.1_GP4_envelope_protein_Porcine_reproductive_and_respiratory_syndrome_virus_2
LRHRDSASEAIRKIPQ
>WOZ07361.1_GP4_envelope_protein_Porcine_reproductive_and_respiratory_syndrome_virus_2
LRHRNSASEAIRKIPQ
>WOZ07351.1_GP4_envelope_protein_Porcine_reproductive_and_respiratory_syndrome_virus_2
LGHDNSPPKTPRKSRQ

>WOZ07341.1_GP4_envelope_protein_Porcine_reproductive_and_respiratory_syndrome_virus_2
LRHGDSSFPTIRKSSQ
>WOZ07331.1_GP4_envelope_protein_Porcine_reproductive_and_respiratory_syndrome_virus_2
LRHGYSPTEAIRKSRQ
>WOZ07321.1_GP4_envelope_protein_Porcine_reproductive_and_respiratory_syndrome_virus_2
LRHNNPSSAAFRKIPQ
>WOZ07311.1_GP4_envelope_protein_Porcine_reproductive_and_respiratory_syndrome_virus_2
LRHGYSPIEAIRKSRQ
>WOZ07301.1_GP4_envelope_protein_Porcine_reproductive_and_respiratory_syndrome_virus_2
LRHGHSSSEATRKSRQ
>WOV65762.1_GP4_envelope_protein_Porcine_reproductive_and_respiratory_syndrome_virus_2
LRHSDSSSEATRKSRQ
>WOV65752.1_GP4_envelope_protein_Porcine_reproductive_and_respiratory_syndrome_virus_2
LRHGHSSSEAFRKSRQ
>WOV65742.1_GP4_envelope_protein_Porcine_reproductive_and_respiratory_syndrome_virus_2
LRHNYPSSAAFRKIPQ
>WOV65732.1_GP4_envelope_protein_Porcine_reproductive_and_respiratory_syndrome_virus_2
LRHGNPSPAAFRKVPQ
>WOV65722.1_GP4_envelope_protein_Porcine_reproductive_and_respiratory_syndrome_virus_2
LRHNYPSSAAFRKTPQ
>WOD45403.1_GP4_envelope_protein_Porcine_reproductive_and_respiratory_syndrome_virus_2
LRHRDSAFEAIRKIPQ
>WOD45396.1_GP4_envelope_protein_Porcine_reproductive_and_respiratory_syndrome_virus_2
LRHRDSAFEAIRKIPQ
>WOD45389.1_GP4_envelope_protein_Porcine_reproductive_and_respiratory_syndrome_virus_2
LRHRDSASEAIRKIPQ
>WOD45382.1_GP4_envelope_protein_Porcine_reproductive_and_respiratory_syndrome_virus_2
LRHRDSASEAIRKIPQ
>WOD45375.1_GP4_envelope_protein_Porcine_reproductive_and_respiratory_syndrome_virus_2
LRHRDSAPEAIRKIPQ
>WOD45368.1_GP4_envelope_protein_Porcine_reproductive_and_respiratory_syndrome_virus_2
LRHRDSAPEAIRKIPQ
>UTS56166.1_GP4_envelope_protein_Porcine_reproductive_and_respiratory_syndrome_virus_2
LRHSNPSSAAFRKIPQ
>UTS56156.1_GP4_envelope_protein_Porcine_reproductive_and_respiratory_syndrome_virus_2
LRHRDSASEAIRKIPQ
>UTS56146.1_GP4_envelope_protein_Porcine_reproductive_and_respiratory_syndrome_virus_2
LRHSNPSSAAFRKIPQ
>UTS56136.1_GP4_envelope_protein_Porcine_reproductive_and_respiratory_syndrome_virus_2
LRHGNASSAAFRKIPQ
>UTS56126.1_GP4_envelope_protein_Porcine_reproductive_and_respiratory_syndrome_virus_2
LRHRDSASEAIRKIPQ
>UTS56116.1_GP4_envelope_protein_Porcine_reproductive_and_respiratory_syndrome_virus_2
LRHGNPSSAAFRKVPQ
>UTS56106.1_GP4_envelope_protein_Porcine_reproductive_and_respiratory_syndrome_virus_2
LRHRDSASEAIRKIPQ
>UTS56096.1_GP4_envelope_protein_Porcine_reproductive_and_respiratory_syndrome_virus_2
LRHGNPSSAAFRKVPQ
>UTS56086.1_GP4_envelope_protein_Porcine_reproductive_and_respiratory_syndrome_virus_2
LRHGNPSSAAFRKVPQ
>UTS56076.1_GP4_envelope_protein_Porcine_reproductive_and_respiratory_syndrome_virus_2
LRHRDSASEAIRKIPQ
>UTS56066.1_GP4_envelope_protein_Porcine_reproductive_and_respiratory_syndrome_virus_2
LRHNNPSSAAFRKIPQ
>UTS56056.1_GP4_envelope_protein_Porcine_reproductive_and_respiratory_syndrome_virus_2
LRHRDSASEAIRKIPQ
>UTS56046.1_GP4_envelope_protein_Porcine_reproductive_and_respiratory_syndrome_virus_2
LRHNNPSSAALRKIPQ
>UTS56036.1_GP4_envelope_protein_Porcine_reproductive_and_respiratory_syndrome_virus_2
LRHRDSASEAIRKIPQ
>QGQ60124.1_GP4_protein_Porcine_reproductive_and_respiratory_syndrome_virus_2
LRHGHSSSEAFRKSRQ
>YP_009505552.1_GP4_envelope_protein_Porcine_reproductive_and_respiratory_syndrome_virus_2
LRHRDSASEAIRKIPQ
>AAD12128.1_GP4_envelope_protein_Porcine_reproductive_and_respiratory_syndrome_virus_2
LRHRDSASEAIRKIPQ
>XWS90769.1_glycoprotein_GP4_Porcine_reproductive_and_respiratory_syndrome_virus_2
LRHSNPPIAAFRKIPQ
>XAH04594.1_glycosylated_protein_4_Porcine_reproductive_and_respiratory_syndrome_virus_2
LRHGNPSPAAFRKIPQ
>QSG71701.1_glycosylated_membrane_protein_Porcine_reproductive_and_respiratory_syndrome_virus_2
LRHGDSSSPTIRKNSQ
>QSG71693.1_glycosylated_membrane_protein_Porcine_reproductive_and_respiratory_syndrome_virus_2
LRHGDSSSPTIRKSSQ
>XQW41305.1_glycosylated_protein_4_Porcine_reproductive_and_respiratory_syndrome_virus_2
LRHSNPSLAAFRKVPQ
>XQW41296.1_glycosylated_protein_4_Porcine_reproductive_and_respiratory_syndrome_virus_2
LRHGYPSSAALRKIPQ
>XQW41287.1_glycosylated_protein_4_Porcine_reproductive_and_respiratory_syndrome_virus_2
LRHGYSSSAALRKIPQ
>XQW41278.1_glycosylated_protein_4_Porcine_reproductive_and_respiratory_syndrome_virus_2
LRHGYSSSAALRKIPQ
>XPH62496.2_glycoprotein_4_Porcine_reproductive_and_respiratory_syndrome_virus_2
LGHGHPSSEAVRKIRQ
>XPH62626.1_glycoprotein_4_Porcine_reproductive_and_respiratory_syndrome_virus_2
LRHGYSSSEAVRKSRQ
>XPH62616.1_glycoprotein_4_Porcine_reproductive_and_respiratory_syndrome_virus_2
LRHGYSSSEAVRKSRQ
>XPH62606.1_glycoprotein_4_Porcine_reproductive_and_respiratory_syndrome_virus_2
LRHGHPPFEAFRKIRQ

>XPH62596.1_glycoprotein_4_Porcine_reproductive_and_respiratory_syndrome_virus_2
LRHGHPPFEAFRKIRQ
>XPH62586.1_glycoprotein_4_Porcine_reproductive_and_respiratory_syndrome_virus_2
LRYNNPSSAALRKIPQ
>XPH62576.1_glycoprotein_4_Porcine_reproductive_and_respiratory_syndrome_virus_2
LRHGNPSVAAFRKIPQ
>XPH62566.1_glycoprotein_4_Porcine_reproductive_and_respiratory_syndrome_virus_2
LRYNNPSSAALRKIPQ
>XPH62556.1_glycoprotein_4_Porcine_reproductive_and_respiratory_syndrome_virus_2
LRHGNPPVAAFRKIPQ
>XPH62546.1_glycoprotein_4_Porcine_reproductive_and_respiratory_syndrome_virus_2
LRHGNPSVAAFRKIPQ
>XPH62536.1_glycoprotein_4_Porcine_reproductive_and_respiratory_syndrome_virus_2
LRHGNPSVAAFRKIPQ
>XPH62526.1_glycoprotein_4_Porcine_reproductive_and_respiratory_syndrome_virus_2
LRHGNPPVAAFRKIPQ
>XPH62516.1_glycoprotein_4_Porcine_reproductive_and_respiratory_syndrome_virus_2
LRHGNPSVAAFRKIPQ
>XPH62506.1_glycoprotein_4_Porcine_reproductive_and_respiratory_syndrome_virus_2
LRHGHSSSEAFRKSRQ
>XPH62486.1_glycoprotein_4_Porcine_reproductive_and_respiratory_syndrome_virus_2
LRHGHPSSEAVRKSRQ
>XPH62476.1_glycoprotein_4_Porcine_reproductive_and_respiratory_syndrome_virus_2
LRHGHSPSEAFRKSRQ
>XPH62466.1_glycoprotein_4_Porcine_reproductive_and_respiratory_syndrome_virus_2
LRHGYSSSEAVRKSRQ
>XPH62456.1_glycoprotein_4_Porcine_reproductive_and_respiratory_syndrome_virus_2
LRHGYSPFEAFRKSRQ
>XPH62446.1_glycoprotein_4_Porcine_reproductive_and_respiratory_syndrome_virus_2
LRHGHSSPEAFRKSRQ
>XPH62702.1_glycoprotein_4_Porcine_reproductive_and_respiratory_syndrome_virus_2
LRHGNPSVAAFRKIPQ
>XPH62692.1_glycoprotein_4_Porcine_reproductive_and_respiratory_syndrome_virus_2
LRHGNPSVAAFRKIPQ
>XPH62688.1_glycoprotein_4_Porcine_reproductive_and_respiratory_syndrome_virus_2
LRHGHSPPEALRKSRQ
>XPH62678.1_glycoprotein_4_Porcine_reproductive_and_respiratory_syndrome_virus_2
LRHGNPSVAAFRKIPQ
>XPH62664.1_glycoprotein_4_Porcine_reproductive_and_respiratory_syndrome_virus_2
LRHGYSSSEAVRKSRQ
>XPH62654.1_glycoprotein_4_Porcine_reproductive_and_respiratory_syndrome_virus_2
LGHGYSSSEAVRKSRQ
>XPH62648.1_glycoprotein_4_Porcine_reproductive_and_respiratory_syndrome_virus_2
LRHGHSPLEALRKSRQ
>XPH62638.1_glycoprotein_4_Porcine_reproductive_and_respiratory_syndrome_virus_2
LRHGHSAPEAFRKSRQ
>XLC13386.1_ORF4_Porcine_reproductive_and_respiratory_syndrome_virus_2
LRHGDSSGEAIRKSRQ
>WCU30814.1_ORF4_Porcine_reproductive_and_respiratory_syndrome_virus_2
LRHRDSASKAIRKIPQ
>UOA04056.1_envelope_glycoprotein_4_Porcine_reproductive_and_respiratory_syndrome_virus_2
LRHGNTPSPTFRKIPQ
>UOA04047.1_envelope_glycoprotein_4_Porcine_reproductive_and_respiratory_syndrome_virus_2
LRHRDSTSKAIRKIPQ
>UOA04038.1_envelope_glycoprotein_4_Porcine_reproductive_and_respiratory_syndrome_virus_2
LGHSNPSHEAFRKVPQ
>UOA04029.1_envelope_glycoprotein_4_Porcine_reproductive_and_respiratory_syndrome_virus_2
LRHRDSTSKAIRKVPQ
>UOA04020.1_envelope_glycoprotein_4_Porcine_reproductive_and_respiratory_syndrome_virus_2
LRHRDSASEAIRKIPQ
>UOA04011.1_envelope_glycoprotein_4_Porcine_reproductive_and_respiratory_syndrome_virus_2
LRHGDSTSKTFRKIPQ
>UOA04002.1_envelope_glycoprotein_4_Porcine_reproductive_and_respiratory_syndrome_virus_2
LRHGNPSPAPFRKIPQ
>UOA03993.1_envelope_glycoprotein_4_Porcine_reproductive_and_respiratory_syndrome_virus_2
LRHRDSASEAIRKIPQ
>UOA03984.1_envelope_glycoprotein_4_Porcine_reproductive_and_respiratory_syndrome_virus_2
LRHGDTSSSAFRKIPQ
>UOA03975.1_envelope_glycoprotein_4_Porcine_reproductive_and_respiratory_syndrome_virus_2
LRHGNPSSASFRKIPQ
>UOA03966.1_envelope_glycoprotein_4_Porcine_reproductive_and_respiratory_syndrome_virus_2
LRHGDTSSPSFRKIPQ
>UOA03957.1_envelope_glycoprotein_4_Porcine_reproductive_and_respiratory_syndrome_virus_2
LRHGHSATKAFRKVPQ
>UOA03948.1_envelope_glycoprotein_4_Porcine_reproductive_and_respiratory_syndrome_virus_2
LRHGNPSSEAARKSRQ
>UOA03939.1_envelope_glycoprotein_4_Porcine_reproductive_and_respiratory_syndrome_virus_2
LRHGNPPPEAVRKSRQ
>QTW05959.1_ORF4_Porcine_reproductive_and_respiratory_syndrome_virus_2
LRHNYPSSAAFRKTPQ
>QKS68475.1_glycosylated_protein_4_Porcine_reproductive_and_respiratory_syndrome_virus_2
LRHSNPPPATFRKIPQ
>QKS68467.1_glycosylated_protein_4_Porcine_reproductive_and_respiratory_syndrome_virus_2
LRHRDSASEAIRKIPQ
>QKS68459.1_glycosylated_protein_4_Porcine_reproductive_and_respiratory_syndrome_virus_2
LRHGDSSSPTIRKSSQ
>QKS68451.1_glycosylated_protein_4_Porcine_reproductive_and_respiratory_syndrome_virus_2
LRHSNPPPATFRKIPQ
>QKS68443.1_glycosylated_protein_4_Porcine_reproductive_and_respiratory_syndrome_virus_2
LRHSNPSPAAFRKVPQ

>QKS68435.1_glycosylated_protein_4_Porcine_reproductive_and_respiratory_syndrome_virus_2
LRHGDSPYPTIRKSSQ
>QKS68427.1_glycosylated_protein_4_Porcine_reproductive_and_respiratory_syndrome_virus_2
LRHGNSPSPAIRKSSQ
>QGD14185.1_glycoprotein_4_Porcine_reproductive_and_respiratory_syndrome_virus_2
LRHGDSSFPTIRKSSQ
>QGD14174.1_glycoprotein_4_Porcine_reproductive_and_respiratory_syndrome_virus_2
LRHGDSSFPTIRKSSQ
>QBH74652.1_envelope_glycoprotein_4_Porcine_reproductive_and_respiratory_syndrome_virus_2
LRHGNSSSKTFRKIPQ
>QBH74642.1_envelope_glycoprotein_4_Porcine_reproductive_and_respiratory_syndrome_virus_2
LRHGNPSPASFRKIPH
>QBH74632.1_envelope_glycoprotein_4_Porcine_reproductive_and_respiratory_syndrome_virus_2
LRHGDSSHQAFRKIPQ
>QBH74622.1_envelope_glycoprotein_4_Porcine_reproductive_and_respiratory_syndrome_virus_2
LRHGDTPGPAFRKVPQ
